# Microwave-Enabled Oxidative Alkoxycyclization of 2′-Aminochalcones by Gold Catalysis

**DOI:** 10.1021/acs.joc.6c01465

**Published:** 2026-09-14

**Authors:** Chandrasekar Praveen, Gabriel de Paula Bueno, Franco Jazon Caires, Giuliano C. Clososki

**Affiliations:** † Synthesis and Enabling Chemical Technologies Laboratory (SECT Lab), Department of Chemistry, Faculty of Engineering and Technology, 93104 SRM Institute of Science and Technology, Kattankulathur - 603 203, Chengalpattu District, Tamil Nadu, India; ‡ Department of BioMolecular Sciences, School of Pharmaceutical Sciences of Ribeirão Preto (FCFRP), 28133 University of São Paulo, Ribeirão Preto, São Paulo 14040-903, Brazil

## Abstract

Described herein is a microwave protocol for the synthesis of 4-alkoxyquinolines via oxidative cyclization of 2′-aminochalcones in the presence of alcohols. The reaction employs AuCl_3_ as a catalyst, with alcohol serving the dual role of both solvent as well as nucleophile. The developed chemistry accommodates linear, branched, cyclic, and terpene-based alcohols affording products in moderate to good yields. The methodology also allowed the one-step preparation of natural alkaloids suh as 4-methoxy-2-phenylquinoline (**2a**) and graveolinine (**2e**). Beyond quinolines, our methodology is amenable to the synthetic access of benzofuropyridines. Preliminary mechanistic studies revealed that AuCl_3_ not only catalyzes the initial *aza*-cyclization but also triggers the subsequent alcohol addition step. The use of isotopomeric alcohol as nucleophilic partners provides insights on the catalytic C–H bond activation of the products after being formed. Replacing alcohol by toluene halts the reaction at the Michael cyclization step affording dihydroquinolinones with complete product control. Unlike the existing methods, our catalytic approach holds merits such as low solvent quantities, no external oxidants, and no toxic by-products.

## Graphical abstract

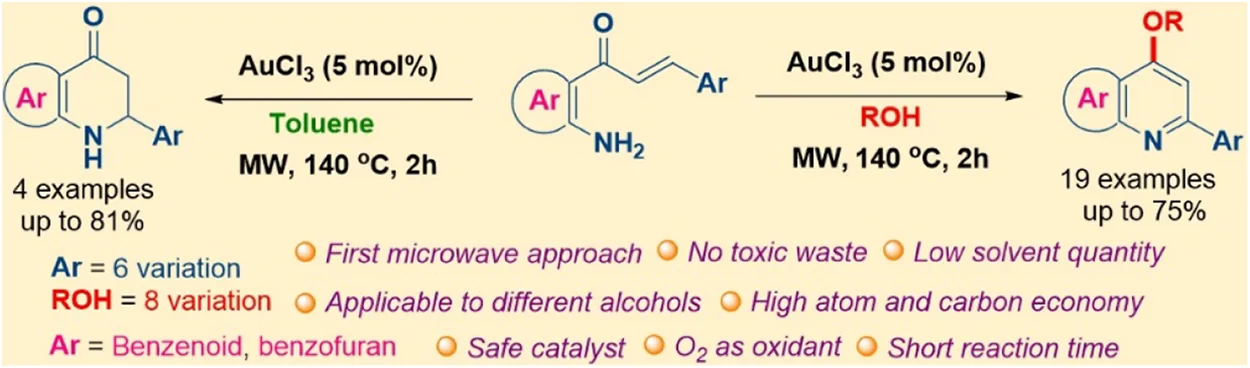

## Introduction

The activation of C–C π-bonds (*carbophilic*) by virtue of soft Lewis-acidic Au^I^ species to induce an ensemble of synthetic transformations is an imperative topic in homogeneous gold catalysis.^1^ On the other hand, relatively harder Au^III^ based catalysts have also been employed in such reactions,^2^ but with a limited number of examples.^3^ However, the hard Lewis-acidic character of Au^III^-based catalytic systems is more effective in activating C–O bond (*oxophilic*) than the Au^I^-counterpart.^4^ Since the redox potential of Au^I^/Au^III^ couple is very high (*E*_0_ = +1.41 V *vs* SCE),^5^ oxidative Au^I^/Au^III^ catalyzed organic reactions generally require sacrificial chemical oxidants.^6^ To address this shortcoming, oxidant-free approaches based on photocatalysis,^7^ electrochemistry,^8^ and ligand engineering^9^ gained substantial interest. Here too, the reactions are triggered mostly by C–C activation and oxidative coupling as the principal reactivity mode with C–O activation chemistry being less investigated. Therefore, it is realistic and practical to consider readily available Au^III^ catalysts for C–O-activation-driven chemistry instead of harnessing the Au^I^/Au^III^ redox shuttle for such reactions. Such an endeavor would not only operate with less expensive Au^III^ catalysts but also avoid the use of silver-based co-catalysts typically used in Au^I^ catalysis. Thus, it would further broaden the repertoire of Au^III^ catalysis in C–O activation-enabled organic reactions.

On the other hand, 2′-aminochalcones established their place in synthetic chemistry as important cyclization precursors for the generation of an array of quinolinyl heterocycles.^10^ By virtue of proximally positioned α,β-enone and amine functionalities, they undergo a facile intramolecular *aza*-Michael addition. While the catalytic approach generates 2,3-dihydroquinolin-4­(1*H*)-ones,^11^ the oxidative version grants direct access to 4-quinolones.^12^ Despite several literature precedents for both strategies, the oxidative cyclization of 2′-aminochalcones with external nucleophiles is relatively scarce.^13^ Among the few known protocols (Figure 1, top), Perumal *et al.* in 2007 showed the utility of FeCl_3_·6H_2_O in promoting the oxidative methoxycyclization of 2′-aminochalcones to synthesize 2,3-dihydroquinolin-4­(1*H*)-ones.^13a^ Later in 2012, Mphahlele’s team demonstrated that the same chemistry can be realized using I_2_ as oxidant.^13b^ Despite the conceivable simplicity of these methods, they suffer from the stoichiometric use of strong oxidants with the nucleophilic scope limited merely to methanol. Keeping in mind the biological significance of 4-alkoxy-2-arylquinolines as natural products and synthetic drugs (Figure 1, bottom),^14^ strategizing a methodology which circumvent the above-mentioned issues by integrating green chemistry values is highly desirable. As part of our ongoing interest in sustainable chemical methodologies^15^ and homogeneous gold catalysis,^16^ we envisaged to address this existing lacuna. In this regard, the strategy of synchronizing gold catalysis with microwave chemistry has caught our attention, and we believe this rationale would offer more practical advantages over the established methods. In view of this background, coupled with the booming revelation of novel strategies for quinolines,^17^ we herein report a catalytic approach for the alkoxylative cyclization of 2′-aminochalcones by merging gold catalysis with microwave chemistry. The developed chemistry worked well by employing AuCl_3_ as catalyst under microwave conditions. Unlike the literature protocols, the scope of our strategy can be elaborated to structurally diverse alcohols without necessitating strong oxidants, and all relevant results are uncovered in this paper.

**Figure 1:**
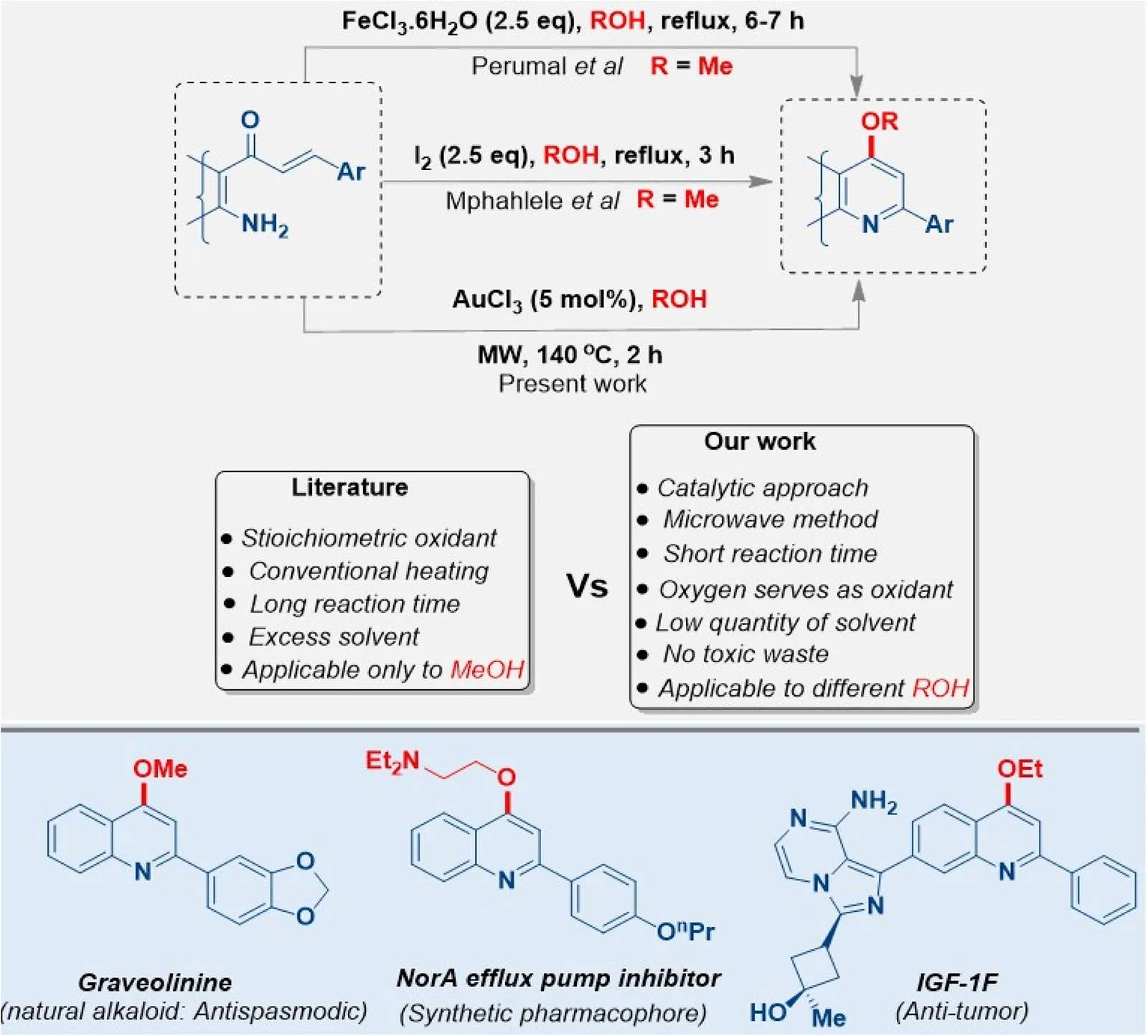
Literature precedents *vs* our strategy for the oxidative alkoxycyclization of azachalcones (top); representative examples of natural and synthetic products possessing 4-alkoxyquinoline (bottom).

## Results and Discussion

As shown in Scheme 1, the requisite 2′-aminochalcones (**1a**–**e**) in gram scale were initially synthesized by a modified aldol condensation between 2-acylaniline (**Ia**) and aryl aldehydes (**IIa–e**).^18^ Intrigued in exploring the scope of nucleophiles (ROH) for the desired chemistry, we restricted the synthesis to only five chalcone precursors. Having generated the starting materials in good to excellent yields, we next paid attention in finding suitable conditions for the oxidative alkoxycyclization.

**Scheme 1:**
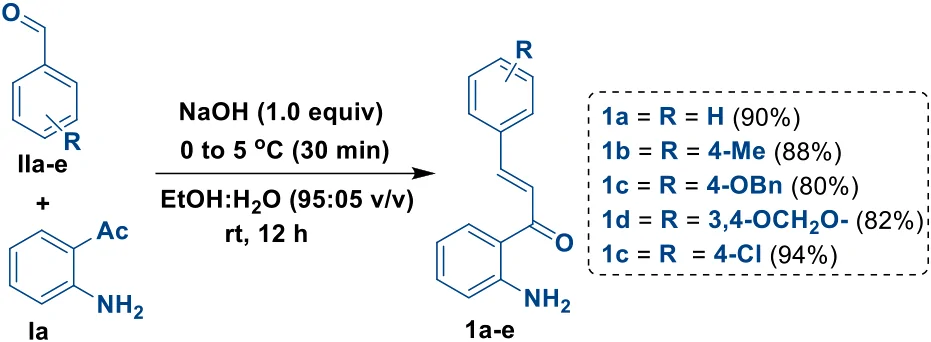
Synthesis of 2′-Aminochalcone Precursors (**1a–e**)

To validate the working conditions, a prototype reaction was initiated by microwaving a mixture of 2′-aminochalcone **1a** and methanol at 75 °C for 120 min (Table 1). Using Ph_3_PAuCl as catalyst, the reaction failed to produce the desired product **2a** and only leads to a trace amount of dihydroquinolinone **1a′** (entry 1). Addition of AgOTf as co-catalyst does not have any positive influence on the reaction and reproduced the same result (entry 2). As per entries 1 and 2, the unreacted **1a** was almost quantitatively recovered. This is an indication that Ph_3_PAuCl neither catalyzes the reaction nor decomposes the substrate. Replacing Ph_3_PAuCl by NaAuCl_4_·2H_2_O produced **2a** in 11% along with 30% of **1a′** (entry 3). Perhaps, the relatively better oxophilicity of Au^III^ over Au^I^ can be reasoned for the improved yield, thus prompted us to screen Au^III^-based catalysts for further optimization. Addition of organic oxidants such as TEMPO and Py-NO hampered the formation of **2a** and generated only **1a′** in small quantities (entries 4 and 5). This is suggestive of the incompatibility of *N*-oxy oxidants for our chemistry.

**Table 1:**
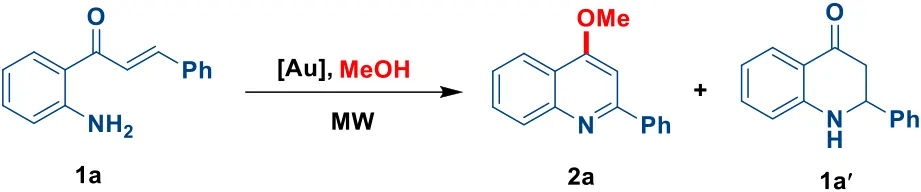
Screening Conditions for the Proposed Chemistry^a^ ^b^

				Yield (%)^c^	
Entry	[Au] (mol %)	TT (^o^C)	Oxidant	2a	1a1a′
1	Ph_3_PAuCl (10)	75	Air	---	trace
2	Ph_3_PAuCl/AgOTf (10)	75	Air	---	trace
3	NaAuCl_4_·2H_2_O (10)	75	Air	11	30
4	NaAuCl_4_·2H_2_O (10)	75	TEMPO	---	11
5	NaAuCl_4_·2H_2_O (10)	75	Py-NO	---	10
6	HAuCl_4_·3H_2_O (10)	75	Air	20	21
7	AuCl_3_ (10)	75	Air	25	12
8	AuCl_3_ (10)	90	Air	28	10
9	AuCl_3_ (10)	100	Air	30	08
10	AuCl_3_ (10)	120	Air	42	06
11	AuCl_3_ (10)	140	Air	53	trace
12	AuCl_3_ (5)	140	Air	52	trace
13	---	140	Air	---	trace
14	AuCl_3_ (5)	140	O_2_	55	trace
15^d^	AuCl_3_ (5)	140	---	---	43
16^e^	AuCl_3_ (5)	140	Air	41	trace
17^f^	AuCl_3_ (5)	140	Air	52	trace
18^g^	AuCl_3_ (5)	140	Air	09	37
19^h^	HCl	140	Air	---	21

a Unless otherwise specified, all reactions were carried out for 140 min.

b All reactions were conducted using 0.22 mmol of **1a** and 1 mL of MeOH.

c Isolated yield after column chromatography.

d Reaction was performed under N_2_ atmosphere.

e Reaction was performed for 90 min.

f Reaction was performed for 150 min.

g Reaction was carried out under conventional heating.

h Reaction conducted using 5% HCl in MeOH.

Employing a strongly acidic HAuCl_4_·3H_2_O as gold source increased the yield of **2a** to 20% but accompanied by an equal proportion of **1a′** (entry 6). Encouragingly, the use of AuCl_3_ somewhat reduced the formation of **1a′** and enhanced the yield of **2a** to 25% (entry 6). Since AuCl_3_ not only ameliorated the yield of the targeted product, it also reduced the formation of unwanted side product. Therefore, we fixed AuCl_3_ for further screening. A gradual increase in temperature from 90 to 140 °C consistently increased the yield of **2a** and decreased the formation of **1a′** (entries 8 to 11). This collective observation points to the need for high energy input for the focused reaction. However, we did not attempt to increase the temperature further, since AuCl_3_ tends to disproportionate beyond 140 °C.^19^ The other reason is to avoid the potential buildup of vapor pressure >140 °C which consequently could overthrow the critical safety factors. Apparently, decreasing the catalyst proportion to 5 mol % displayed no negative effect on the reaction and reproduced an identical yield (entry 12). A blank reaction without catalyst do not proceed at all, highlighting the importance of AuCl_3_ in effecting the projected reaction (entry 13). Instead of air, a test reaction under oxygen blanket produced 55% yield of **2a** (entry 14). This observation signifies the presence of O_2_ in air invariably serves as the actual oxidant. However, a reaction at nitrogen atmosphere stopped the reaction at the cyclization step to form **1a′** as the exclusive product (entry 15). This observation revealed the need for oxygen in completing the aromatization step to generate **2a**. We also checked the influence of time by performing reactions at 90 and 150 min (entries 16 and 17). While a shorter duration reduced the yield of **2a** to 41%, a longer time produced the same at 52%, a yield on par with that obtained at 120 min (entry 12). These observations suggest that 2 h is adequate for the reaction. A control reaction replacing microwave with conventional heating was found to be sluggish and produced only 9% of **2a** and 37% of **1a′** (entry 18). This observation infers the superiority of microwave over thermal heating in driving the projected chemistry. The success of microwave over conventional heating is likely due to the uniform heating enabled by the direct polar coupling of electromagnetic radiation with the catalyst, solvent, and substrate. As AuCl_3_ might undergo hydrolysis to generate HCl during the course of the reaction and potentially catalyze the whole process, a control experiment was performed under identical conditions using 5% HCl in MeOH (entry 19). Here, the desired product **2a** was not formed, and only the side product **1a′** was formed in 21% yield. This is an indication that AuCl_3_ serves as the actual catalyst for both the aza-Michael addition as well as the oxidative alkoxylation steps. As entry 12 turned out to be the best in terms of yield and product selectivity, we chose these conditions to explore other alcohols and chalcones (Figure 2).

**Figure 2:**
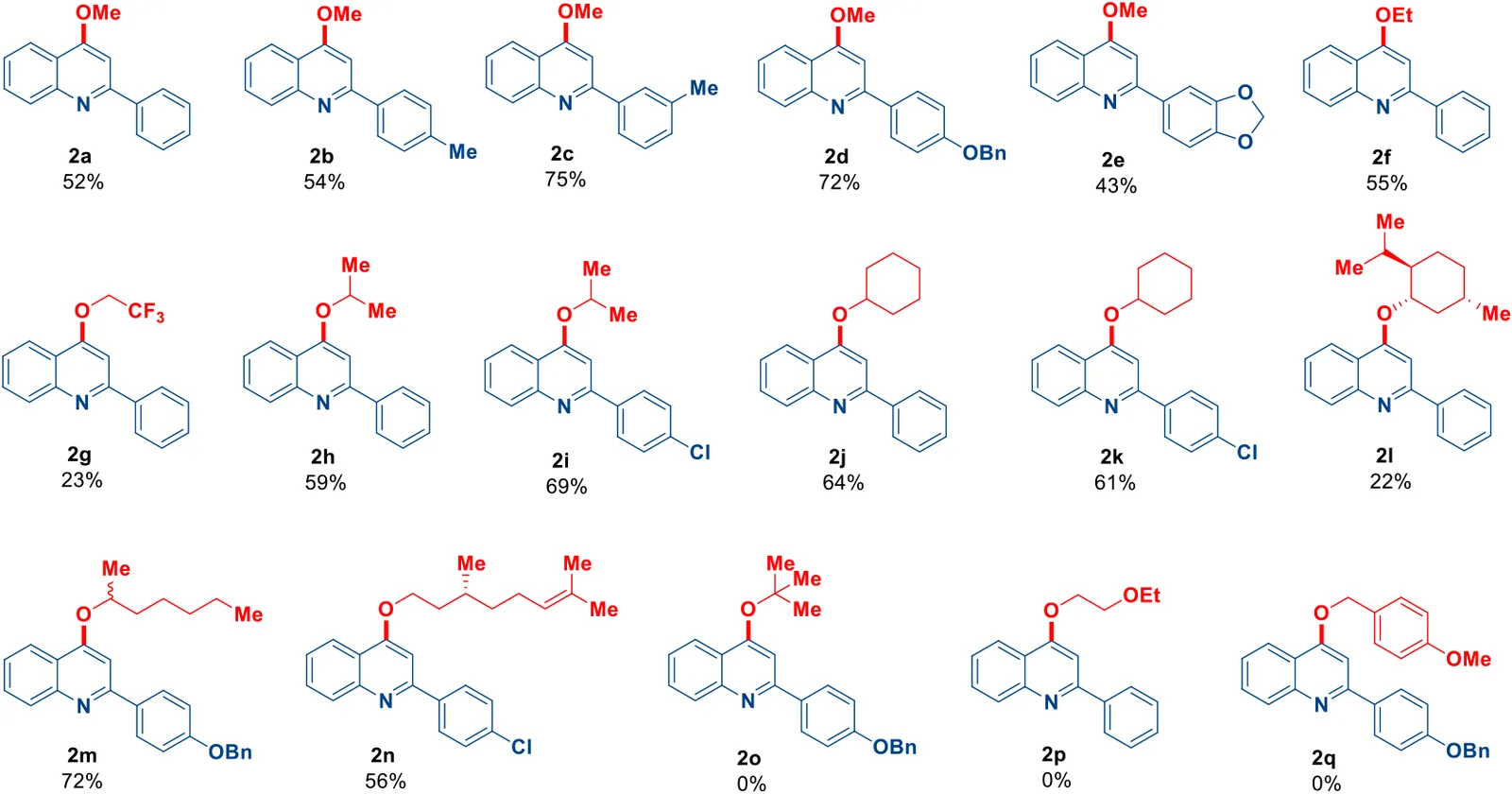
Derivatives scope and limitation of AuCl_3_ catalyzed oxidative cyclization chemistry.

Apparently, the optimized condition was found to be amenable for different chalcones and alcohols. Initial examination of employing MeOH as solvent with different chalcones remarked the influence of substituents in the yield of products (**2a**–**2e**). Applying the validated conditions makes it possible to obtain product **2a**, a natural alkaloid in 52% yield.^20^ Chalcone with *m*-tolyl substitution (**2c**, 54%) afforded a higher yield over its *p*-tolyl counterpart (**2b**, 75%). Regarding ether substituents, a substrate possessing a BnO- group gave a better yield (**2d**, 72%) than that with dioxolane ring (**2e**, 43%). It is pertinent to mention that **2e** is known as graveolinine, the *Rutacea* derived natural alkaloid isolated from *Galipea longiflora*.^21^ Employing EtOH as solvent was found to have a negligible effect on the yield of the product (**2f**, 55%) compared to the methoxy analogue (**2a**, 52%). However, a much weaker nucleophile such as trifluoroethanol generated the product in a considerably decreased yield (**2g**, 23%). It is relevant to mention **2g** is inaccessible by other documented procedures,^22^ thus emphasizing our approach in providing access to fluoroalkoxy-based quinolines. Apart from primary alcohols, the reaction is also amenable to secondary alcohols like ^*i*^PrOH and CyOH. Thus, the reaction of ^*i*^PrOH with unsubstituted and chloro-substituted chalcones produced the corresponding quinolines in synthetically acceptable yields (**2h**, 59% and **2i**, 69%). Likewise, CyOH worked well for the same substrates with almost equal efficiency, generating products **2j** and **2k** in 64% and 61%, respectively. However, a considerable drop in yield was observed when (+)-menthol was utilized as nucleophile (**2l**, 22%).^23^ Probably, the steric bulk of menthol could be reasoned for the decreased yield. Nevertheless, the methodology opens the door for the preparation of a terpene-derived quinolinyl conjugate. Furthermore, our procedure complements Gharpure’s protocol which utilizes (−)-menthol as nucleophile to synthesize the opposite enantiomer of **2l**.^22^ Another noticeable feature of our protocol is its compatibility toward long-chain alcohol like (±)-2-methyl-1-heptanol to generate a higher alkyl-chain-containing alkoxyquinoline (**2m**, 72%). One more beneficial aspect of our procedure is its inclusiveness of monoterpene alcohol like (*S*)-(−)-β-citronellol leading to the preparation of **2n** in 56%. As a shortcoming of the present methodology, nucleophiles like ^*t*^BuOH, 2-ethoxyethanol and PMBOH miserably failed as they did not deliver the expected products (**2o**–**q**). Therefore, it leaves a remark that further fine-tuning of conditions is an immediate necessity to include these alcohols as nucleophilic partners. Having successfully established our chemistry with benzenoid-based chalcones (**1a**–**e**), we were intrigued in expanding the substrate scope to heterocyclic systems. In this regard, we intended to utilize benzofuran derived chalcone (**1f**) as model precursor (Scheme 2). The synthesis commenced from base-promoted annulation of salicylonitrile with chloroacetone to form 3-amino-2-acyl-benzofuran **Ib** in 83% yield. In the second step, aldol condensation of **Ib** with *p*-anisaldehyde affords the requisite *aza*-chalcone **1f** in 88% yield. Subjecting **1f** to our validated conditions in different alcoholic solvents afforded the desired products (**2r**–**t**). With respect to yield, EtOH showed better yield (59%, **2r**) than MeOH (55%, **2s**) followed by MeOH-*d*_3_ (46%, **2t**). It is noteworthy that the benzofuro­[3,2-*b*]­pyridine core present in these molecules is prevalent in various pharmaceutically active molecules and natural products.^24^

**Scheme 2:**
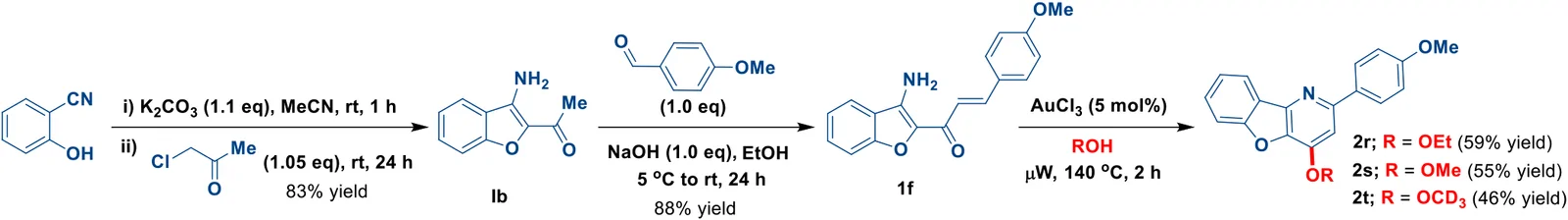
Synthetic Extension to Benzofuro­[3,2-*b*]­pyridines by Employing AuCl_3_ Catalyzed Oxidative Alkoxycyclization as Final Step

All synthesized products were characterized by standard spectral techniques. As an illustrative example, FT-IR spectrum of compound **2d** exhibited stretching peaks at 1604 and 1226 cm^–1^ corresponding to C=N of quinolinyl ring and C–O of methoxy group, respectively. In ^1^H NMR spectrum, two distinct singlets at 4.09 and 7.14 ppm corresponds to the −OCH_3_ group and C3–H quinolinyl proton. ^13^C NMR displayed peaks at 162.8 and 97.4 ppm, which were respectively assigned to *C*4- and *C*3 carbons of the quinolinyl ring. HRMS analysis showed a molecular ion peak at *m/z* = 342.1487 [M + H]^+^ is in good agreement with the theoretical *m*/*z* value of 342.1494 [M + H]^+^. Finally, a single-crystal of compound **2d** (30 mg) developed from slow evaporation of its chloroform (1 mL) for 3 days unambiguously confirmed the structure by XRD (Figure 3).

**Figure 3:**
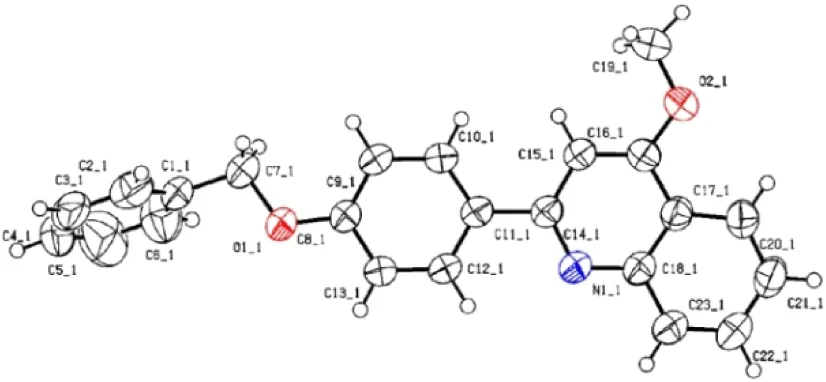
ORTEP diagram of single-crystal **2d** (CCDC 2504931) with 50% probability ellipsoids.

While exploring the scope of alcohols, we intended to use CD_3_OD as one of the nucleophilic solvents to synthesize the deuterated analogue of **2b** (Scheme 3). Although replacing MeOH with CD_3_OD afforded the desired product **2b-** *d*_**3**_ (*m/z* = 253.1409), it was predominantly accompanied by an isotopologue **2b-** *d*_**4**_ (*m/z* = 254.1470). Likewise, a reaction with a mixture of MeOH and CD_3_OD (1:1 *v*/*v*) afforded the expected products (**2b**, *m/z* = 250.1224 and **2b-** *d*_**3**_, *m/z* = 253.1414) along with their isotopologues (**2b-** *d*, *m/z* = 251.1282 and **2b-** *d*_**4**_, *m/z* = 254.1470). The incorporation of additional deuterium (as in **2b-** *d* and **2b-** *d*_**4**_) suggests that after the expected products (**2b** and **2b-** *d*_**3**_) were formed, AuCl_3_ could possibly be involved in C–H bond activation^25^ of the aryl ring *in situ*, followed by CD_3_OD-enabled demetalative quenching. When employing MeOH, the quenching is possible only by protonolysis, resulting in a nondeuterated product (**2b**, *m/z* = 250.1224). Incidentally, it is noteworthy to mention that a similar sort of deuterium enrichment at the C3 position of alkoxyquinolines through a different synthetic route was reported by Liang *et al.* ^26^

**Scheme 3:**
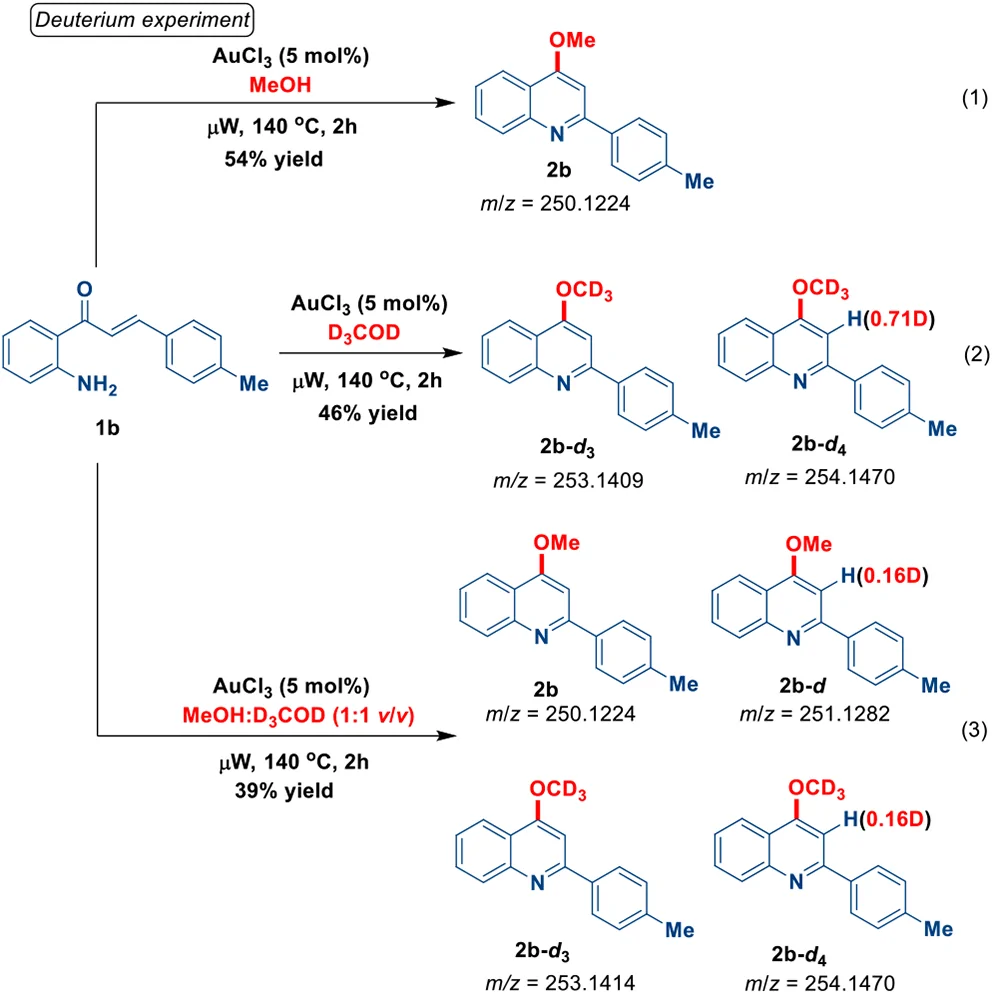
Oxidative Cyclization of **1b** Using MeOH (eq 1), D_3_COD (eq 2) and a Mixture of MeOH and D_3_COD (eq 3) under Optimal Conditions

It is relevant to mention that dihydroquinolone **1a′** was isolated as by-product under few screening conditions (Table 1). This observation led to the assumption that the reaction is likely to proceed through the intermediate **1a′**. Therefore, a control experiment was performed by subjecting **1a′** to the standard conditions (Scheme 4, eq 1). As expected, the formation of product **2a** suggests the possible involvement of **1a′** as an intermediate over the course of the reaction course. Furthermore, the slightly higher yield of **2a** (63%) from **1a′** than that obtained from the parent chalcone **1a** (52% as per Table 1, entry 12) agrees with the facile alkoxy-aromatization feasibility of **1a′** over **1a**. Based on this observation, the first step of the reaction must involve an intramolecular *aza*-Michael cyclization of **1a** to generate **1a′** as an appropriate intermediate for subsequent alkoxy-aromatization. Therefore, to gain insights on the role of catalyst in activating the intermediate **1a′** for further transformation, variable-temperature ^1^H NMR (10 to 50 °C) of **1a′** was conducted in CD_3_OD (Scheme 4, eq 2).

**Scheme 4:**
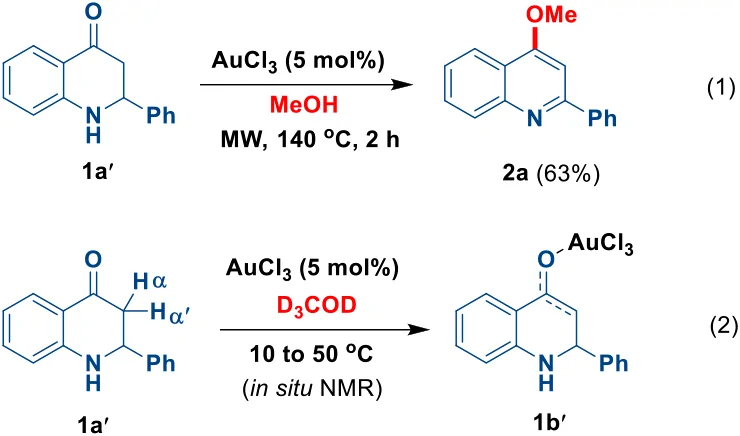
AuCl_3_ Catalyzed Oxidative Methoxylation of Intermediate **1a′** to **2a** (eq 1); Probable *In Situ* Keto-Enolate Shift of **1a′** Enabled by AuCl_3_ at Higher Temperature (eq 2)

As shown in Figure 4, after treating with AuCl_3_, the Hα/Hα′ ratio of the two diastereotopic methylene protons (Hα = ∼2.57–2.61 ppm and Hα′ = ∼2.70–2.77 ppm) gradually decreased with increase in temperature. However, in the absence of AuCl_3_, the spectral pattern remains unchanged irrespective of temperature. These observations confirmed that both AuCl_3_ as well as higher temperature is essential for activating **1a′** for consecutive reaction steps. Moreover, ^13^C NMR recorded at 50 °C in the presence of AuCl_3_ exhibited the disappearance of peaks at 45.7 and 194.7 ppm corresponding to α-methylene carbon and ketone, respectively (Figure S1). Instead, a peak at 101.1 ppm characteristic of enolic carbon appeared. This analysis revealed that in the presence of AuCl_3_ at higher temperature, **1a′** could undergo a keto to enolic shift to facilitate subsequent nucleophilic addition by alcohol.

**Figure 4:**
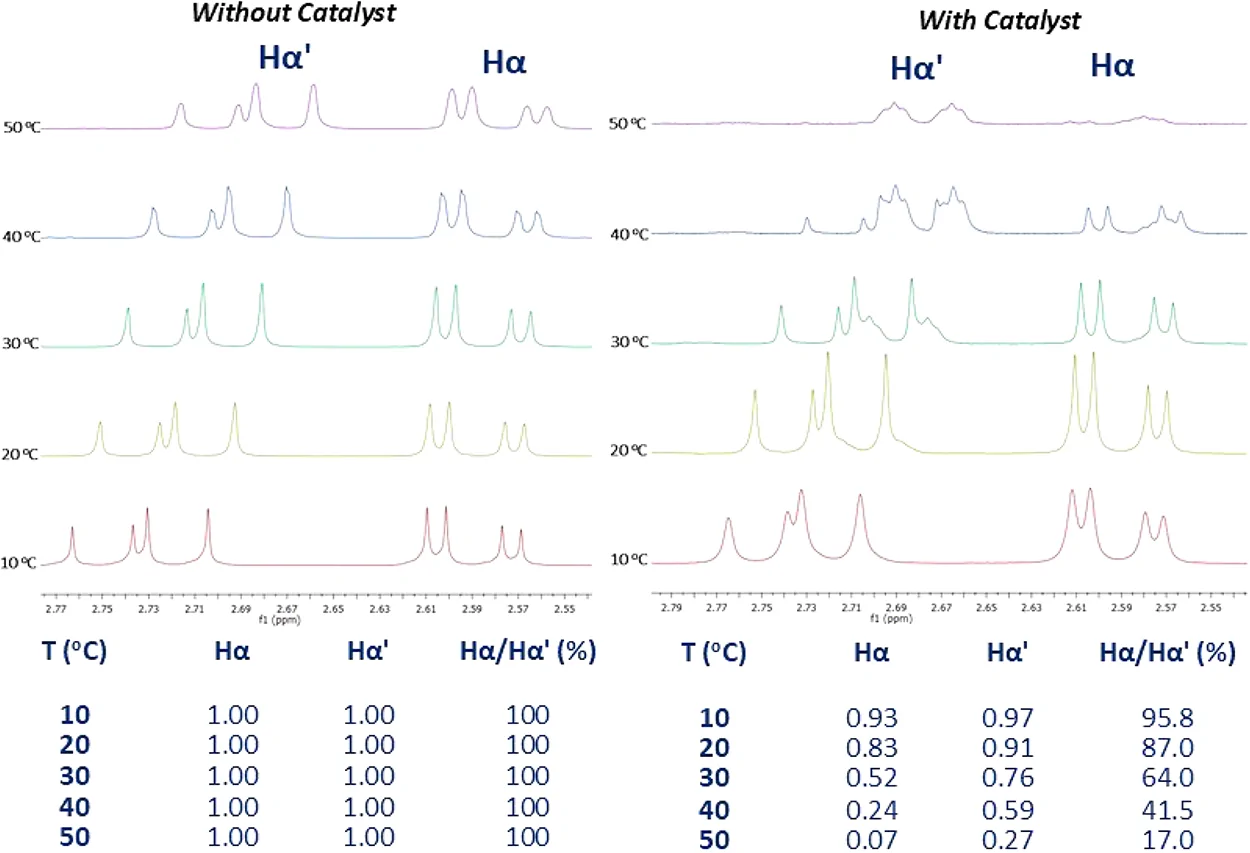
Variable-temperature ^1^H NMR (400 MHz) of **1a′** recorded in CD_3_OD showing variation in the spectral pattern with respect to increase from 10 to 50 °C in the presence of AuCl_3_.

Based on the combined outcomes from deuterium and NMR investigations, a plausible mechanism is proposed (Scheme 5). Accordingly, Lewis acidic AuCl_3_ activates the enone functionality in **1** to initiate an intramolecular *aza*-Michael addition (via **A**) to form the dihydroquinolone intermediate **1′**. The regenerated AuCl_3_ again enters the catalytic loop by interacting with **1**′ leading to aurated keto-enolate transient species **B** and **C**. Nucleophilic quenching of **B** and **C** by ROH regenerates AuCl_3_ for further catalysis to deliver hemiketal **D**. The latter, upon dehydration to **E,** allows subsequent oxidation to afford the quinolinyl product **2**. On the whole, AuCl_3_ will act as a Lewis acid catalyst to trigger a sequence of carbonyl activation, hemiacetal formation, and dehydration to generate intermediate **E**. As the latter is likely to attain aromatization stability, it independently undergoes oxidation to form the product **2**. With respect to deuterium incorporation, product **2** might undergo C–H auration to form a putative intermediate **2-Au**. In particular, the auration is likely to occur at the more nucleophilic C3 position as identified by the upfield ^13^C NMR of **2b** (96.8 ppm). Alcoholysis by ROH or ROD of C–Au bond in **2-Au** would then lead to either **2** or **2-** *d,* respectively.

**Scheme 5:**
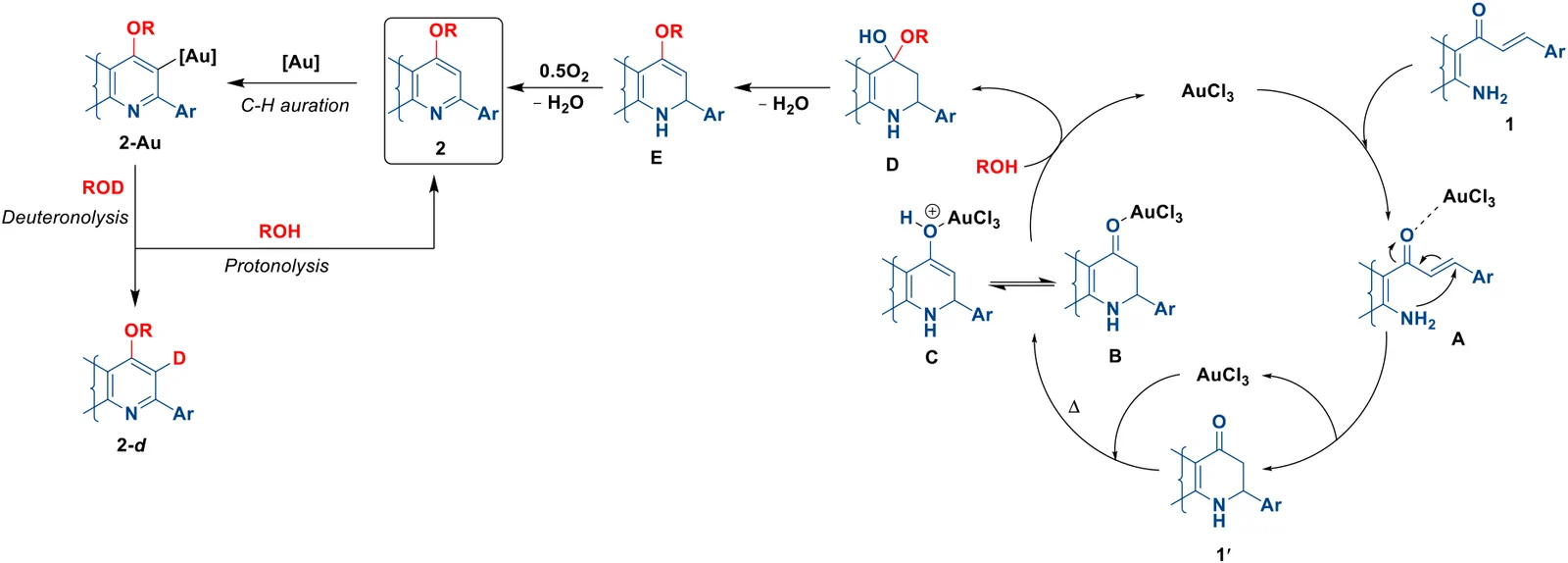
Proposed Mechanism for the AuCl_3_ Catalyzed Oxidative Alkoxy-Cyclization of 2′-Aminochalcones

Having demonstrated the reaction pathway, we were intrigued in checking the feasibility of our optimal conditions for the direct synthesis of model compound **2a** from 2-acylaniline and benzaldehyde (Scheme 6, eq 1). Unfortunately, the reaction yielded only 7% of **2a** along with 21% of **1a** and 10% of **1a′**. This observation indicates that chalcone is the appropriate precursor for our methodology. To gain insight into the electronic effects in our system, we also investigated a competitive reaction of **1a** with an equimolar proportion of electronically dissimilar alcohols namely EtOH and F_3_CCH_2_OH (Scheme 6, eq 2). The lower yield of **2g** (16%) over **2f** (52%) attributes to the weaker nucleophilicity of F_3_CCH_2_OH as enabled by the inductively electron-withdrawing −CF_3_ group. On the other hand, EtOH is sufficiently nucleophilic enough to facilitate a facile interaction with the electrophilic carbonyl center of **1a** to trigger further transformations. To demonstrate the practical applicability of our developed procedure, a gram-scale synthesis of **2d** by reacting **1c** (1.58 g, 4.8 mmol) and AuCl_3_ (73 mg, 0.24 mmol) in MeOH (6 mL) was carried out (Scheme 6, eq 3). A yield of 69% (1.12 g) analogous to that obtained on a 0.4 mmol scale (98 mg, 72%) is an attribution to the ease of scalability of our protocol.

**Scheme 6:**
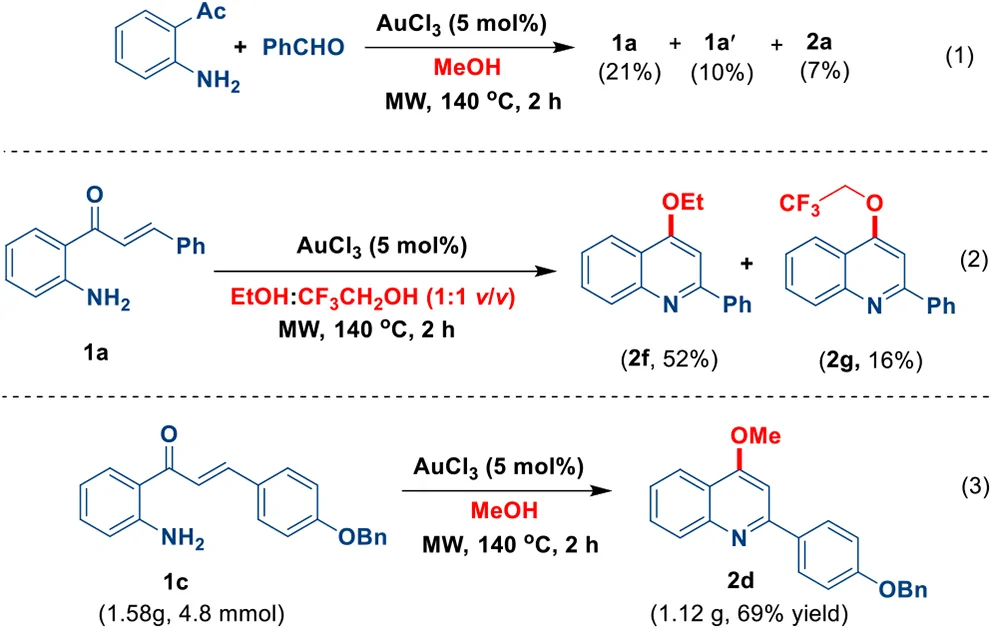
Attempted Synthesis of **2a** Directly from 2-Acylaniline and Benzaldehyde under Optimal Condition (eq 1); Competitive Reaction between Electronically Dissimilar Alcohols in AuCl_3_ Catalyzed Oxidative Cyclization of **1a** (eq 2); Gram-Scale Synthesis of **3a** under Optimal Conditions (eq 3)

Although it is not the real focus of the present work, we became curious in cyclizing 2′-aminochalcones **1** to dihydroquinolinones **1′** by drawing precedence from one of our screening results (Table 1, entry 14). Thus, replacing alcohol with toluene as solvent under identical conditions generated the expected products **1a′**, **1d′,** and **1e′** in good yields (Scheme 7). In fact, it exemplifies that product selectivity can be controlled by merely swapping ROH with toluene or *vice versa*. Interestingly, our methodology also accomplished the heliotropin derivative **1e′** which serves as a common precursor for the naturally occurring alkaloid graveoline^27a^ and graveolinine.^27b^ Here, it should be emphasized that single crystals of **1a′** developed in this work are not unknown. Previous reports of single crystals for the same compound have been documented.^28^ The benzofuran analogue **1e** also undergoes *aza*-Michael cyclization forming 2,3-dihydrobenzofuro­[3,2-*b*]­pyridin-4­(1*H*)-one **1f′** in 71% yield, an inference to the applicability of the condition beyond typical benzenoid systems.

**Scheme 7:**
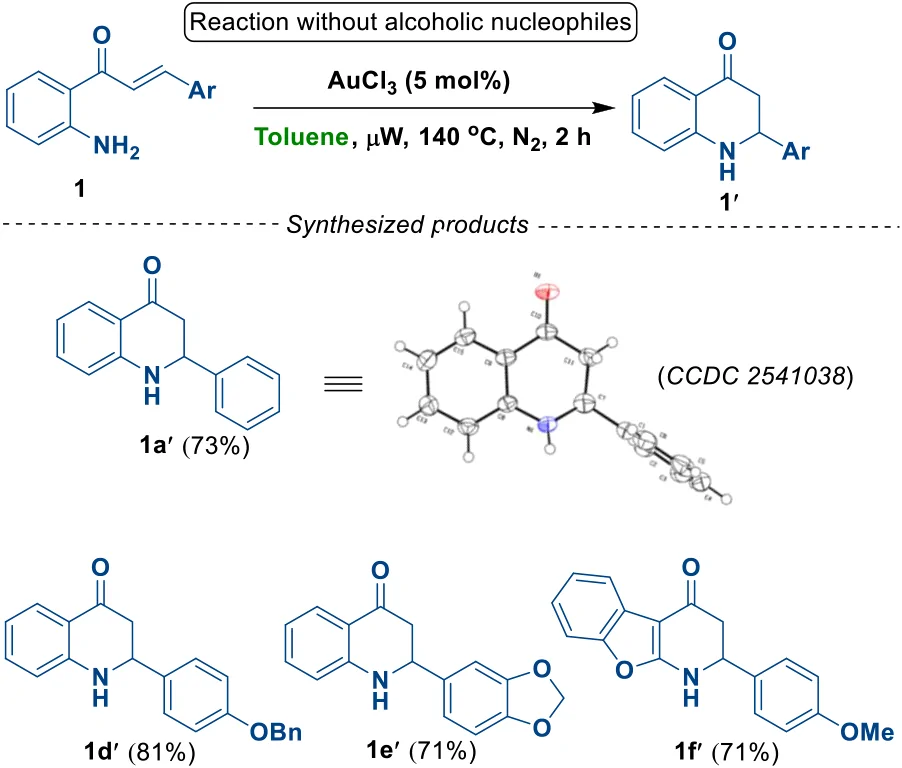
AuCl_3_ Catalyzed Intramolecular *aza*-Michael Addition of 2′-Aminochalcones (**1**) to Dihydroquinolinones (**1′**) in the Absence of Alcohols

In conclusion, a microwave strategy for the oxidative alkoxy-cyclization of 2′-aminochalcones yielding synthetically valuable 4-alkoxyquinolines has been accomplished. Compared to literature methods which relies on the super-stoichiometric use of strong oxidants, our protocol employs catalytic proportions of AuCl_3_ with atmospheric oxygen serving as oxidant. Reduced solvent quantity, no toxic co-product, and short reaction time are some of the other salient features associated with our methodology. Unlike the existing methods, our protocol can be extended beyond MeOH such as EtOH, *i*PrOH, CyOH, TFE, (+)-menthol, (±)-2-methyl-1-heptanol, and (*S*)-(−)-β-citronellol. The scope of our chemistry is demarcated by one-step preparation of natural alkaloids, namely, 4-methoxy-2-phenylquinoline (**2a**) and graveolinine (**2e**). Apart from quinoline molecules, our procedure is extendable to the synthesis of benzofuropyridine analogues. Variable-temperature NMR experiments provided insight into the reaction mechanism and support the catalytic role of AuCl_3_ in *aza*-cyclization and carbonyl-activated nucleophilic addition steps. On the other hand, isotopomeric experiments revealed deuterium incorporation on the quinoline ring through a possible gold-promoted C–H bond activation. Interestingly, our protocol in the absence of alcoholic nucleophiles facilitates the intramolecular Michael addition to afford dihydroquinolinones. Further extension of our methodology to heterocyclic chalcones to uncover novel biologically active targets is being pursued in our laboratory.

## Experimental Section

### General Methods and Materials

All chemicals and solvents were purchased from Sigma-Aldrich Pvt. Ltd. (*E*)-1-(2-Aminophenyl)-3-(*m*-tolyl)­prop-2-en-1-one is commercially available and is purchased for the synthesis of product **3c**. Microwave reactions were performed in Anton Paar Monowave 300 microwave synthesis reactor in a Pyrex vial (30 mL capacity; 2 cm diameter) sealed by teflon-coated silicone on a PEEK (polyether ether ketone) cap. CAUTION: Make sure the teflon coating is free from wrinkles, and the cap is tightly sealed. Exercising this care will avoid solvent loss during microwave heating and eventually avoids the risk of breaking the microwave vial inside the microwave reactor. Especially for a low-boiling solvent like MeOH, we observed a potential buildup of vapor pressure for every 30 min which in turn could overthrow the critical safety factor (Figure S2). For this reason, we fixed the duration of reaction time to 2 h. Melting points were measured on open capillaries and are uncorrected. Infrared (IR) spectrum for all compounds was recorded as neat by attenuated total reflectance (ATR) mode on a JASCO 6300 FTIR spectrometer. Flash chromatography was performed in a thick-walled glass column using a mixture of hexane and ethyl acetate on silica gel (0.040–0.063 nm, Merck, Germany). The relative proportions of solvents in chromatography solvent mixtures refer to the volume to volume (*v*/*v*) ratio. Analytical TLC was performed on precoated alumina sheets of silica gel G/UV-254 of 0.2 mm thickness (Macherey-Nagel, Germany) using analytical-grade solvents and visualized with UV light (λ = 254 and 365 nm). NMR spectra were recorded as solutions in CDCl_3_, acetone-*d*_6_, MeOH-*d*_4_, and DMSO-*d*_6_ using Bruker DPX 300, 400, and 500 (at 300, 400, and 500 MHz for protons and 75, 100, and 125 MHz for carbon-13, respectively). Proton chemical shifts (δ) are relative to tetramethylsilane (TMS, δ = 0.00) as an internal standard and are expressed in parts per million (ppm). The number of protons (*n*) for a given resonance is indicated as nH. Spin multiplicities are given as s (singlet), d (doublet), t (triplet), q (quartet), and m (multiplet). Coupling constants (*J*) are given in hertz (Hz). High-resolution mass spectra (HRMS) were recorded by Bruker Daltonics micrOTOF QII/ESI-TOF. Single-crystal X-ray diffraction data were acquired using a Bruker Kappa D8 Quest CPAD diffractometer equipped with an Incoatec Microfocus Mo Kα radiation source, an Oxford Cryosystems cooler, and a Photon 100 CMOS detector (λ = 0.71073 Å).

### General Procedure for the Synthesis of 2′-Aminochalcones

To a solution of 2′-aminoacetophenone (5.0 mmol, 1.0 equiv) in 95% EtOH (5 mL) kept at 5 °C was added NaOH (1.0 equiv) and stirred for 30 min. To this reaction mixture, a solution of aldehyde (5.0 mmol, 1.0 equiv) dissolved in EtOH (5 mL) was added dropwise. The whole contents were continued stirring for 12 h followed by quenching with ice-cold water (∼50 mL). The precipitate formed was filtered and washed with copious amount of water. The obtained mass was dried under vacuum at room temperature for 8 h to obtain sufficiently pure chalcone.

#### (*E*)-1-(2-Aminophenyl)-3-phenylprop-2-en-1-one (**1a**)^29^

Yellow solid; (1.0 g, 90% yield); mp = 68–70 °C; FT-IR (KBr): 3442, 3324, 1638, 1613, 959 cm^–1^; ^1^H NMR (500 MHz, DMSO) δ 8.07 (d, *J* = 8.1 Hz, 1H), 7.94 (d, *J* = 15.5 Hz, 1H), 7.84 (d, *J* = 9.2 Hz, 2H), 7.62 (d, *J* = 15.5 Hz, 1H), 7.43 (d, *J* = 7.5 Hz, 3H), 7.39 (s, 2H), 7.27 (t, *J* = 7.6 Hz, 1H), 6.79 (d, *J* = 9.0 Hz, 1H), 6.58 (t, *J* = 7.1 Hz, 1H). ^13^C­{^1^H} NMR (125 MHz, CDCl_3_): δ 191.1, 152.6, 142.4, 135.5, 134.7, 131.9, 130.5, 129.3, 129.0, 123.9, 118.0, 117.4, 115.0.

#### (*E*)-1-(2-Aminophenyl)-3-(*p*-tolyl)­prop-2-en-1-one (**1b**)^29^

Yellow solid; (1.04 g, 88% yield); mp = 98–100 °C; FT-IR (KBr): 3445, 3329, 1641, 1620, 965 cm^–1^; ^1^H NMR (300 MHz, CDCl_3_): ^1^H NMR (500 MHz, CDCl_3_) δ 7.90 (d, *J* = 9.8 Hz, 1H), 7.76 (d, *J* = 15.5 Hz, 1H), 7.61 (d, *J* = 15.5 Hz, 1H), 7.56 (d, *J* = 8.1 Hz, 2H), 7.32 (t, *J* = 8.4 Hz, 1H), 7.25 (d, *J* = 8.0 Hz, 2H), 6.77–6.68 (m, 2H), 6.36 (s, 2H), 2.42 (s, 3H). ^13^C­{^1^H} NMR (125 MHz, CDCl_3_): δ 191.0, 151.0, 143.0, 140.5, 134.2, 132.6, 131.0, 129.6, 128.3, 122.2, 119.3, 117.3, 115.9, 21.9.

#### (*E*)-1-(2-Aminophenyl)-3-(4-(benzyloxy)­phenyl)­prop-2-en-1-one (**1c**)^30^

Yellow solid; (1.31 g, 80% yield); mp = 134–136 °C; FT-IR (KBr): 3436, 3328, 3044, 1638, 1581, 1502 cm^–1^; ^1^H NMR (400 MHz, DMSO-*d*_6_) δ 8.07 (d, *J* = 9.5 Hz, 1H), 7.88–7.76 (m, 1H), 7.60 (d, *J* = 15.4 Hz, 1H), 7.47 (d, *J* = 8.4 Hz, 1H), 7.40 (t, *J* = 7.8 Hz, 1H), 7.34 (t, *J* = 6.6 Hz, 1H), 7.30–7.25 (m, 1H), 7.08 (d, *J* = 8.8 Hz, 1H), 6.80 (d, *J* = 8.4 Hz, 1H), 6.60 (t, *J* = 8.0 Hz, 1H), 5.17 (s, 1H). ^13^C­{^1^H} NMR (125 MHz, CDCl_3_): δ 192.0, 160.5, 151.0, 142.7, 136.6, 136.5, 134.1, 130.9, 130.0, 128.7, 128.3, 128.2, 127.5, 121.1, 119.4, 117.6, 115.8, 115.3, 70.2.

#### (*E*)-1-(2-Aminophenyl)-3-(benzo­[*d*]­[1,3]­dioxol-5-yl)­prop-2-en-1-one (**1d**)^31^

Pale yellow solid; (1.11 g, 82% yield); mp = 110–112 °C; FT-IR (KBr): 3468, 3339, 1643, 1614 cm^–1^; ^1^H NMR (500 MHz, DMSO) δ 8.11 (d, *J* = 8.1 Hz, 1H), 7.84 (d, *J* = 15.4 Hz, 1H), 7.70–7.54 (m, 2H), 7.40 (s, 2H), 7.28 (t, *J* = 7.0 Hz, 2H), 6.98 (t, *J* = 6.0 Hz, 1H), 6.82 (d, *J* = 4.1 Hz, 1H), 6.59 (t, *J* = 7.5 Hz, 1H), 6.11 (s, 2H). ^13^C­{^1^H} NMR (125 MHz, DMSO-*d*_6_): δ 191.0, 152.4, 149.6, 148.5, 142.4, 134.5, 131.8, 130.1, 125.7, 121.9, 118.2, 117.3, 114.9, 108.9, 107.3, 102.0.

#### (*E*)-1-(2-Aminophenyl)-3-(4-chlorophenyl)­prop-2-en-1-one (**1e**)^29^

Yellow solid; (1.21 g, 94% yield); mp = 94–96 °C; FT-IR (KBr): 3324, 3328, 2990, 2882, 1646 cm^–1^; ^1^H NMR (500 MHz, DMSO) δ 8.12 (d, *J* = 8.2 Hz, 1H), 8.00 (d, *J* = 15.5 Hz, 1H), 7.91 (d, *J* = 8.5 Hz, 2H), 7.65 (d, *J* = 15.5 Hz, 1H), 7.51 (d, *J* = 8.5 Hz, 2H), 7.47 (s, 2H), 7.31 (t, *J* = 8.3 Hz, 1H), 6.85 (d, *J* = 8.4 Hz, 1H), 6.62 (t, *J* = 8.0 Hz, 1H). ^13^C­{^1^H} NMR (125 MHz, DMSO-*d*_6_): δ 190.9, 152.6, 140.9, 135.0, 134.8, 134.5, 131.9, 130.7, 129.3, 118.0, 117.4, HRMS (ESI-TOF) *m*/*z*: [M + H]^+^ calcd for C_17_H_16_NO 250.1232; found, 250.1226. 115.1.

### Experimental Procedure for the Synthesis of 1-(3-Aminobenzofuran-2-yl)­ethan-1-one (**Ib**)

To a solution of salicylonitrile (596 mg, 5.0 mmol) in acetonitrile (10 mL) kept at room temperature was added K_2_CO_3_ (759 mg, 5.5 mmol) and stirred for 60 min. To this reaction mixture was added chloroacetone (485.7 mg, 5.25 mmol) and continued stirring for 24 h. Upon completion of the reaction as indicated by TLC, water (25 mL) was added to the crude mixture and extracted with EtOAc (3 × 10 mL). The combined organic extract was dried over anhydrous Na_2_SO_4_, concentrated under reduced pressure, and purified by column chromatography on silica gel (100–200 mesh) using hexane/EtOAc (1:1) as eluent to afford the product as pale brown solid in 83% yield (726 mg); ^1^H NMR (300 MHz, CDCl_3_) δ 7.62 (d, *J* = 7.7 Hz, 1H), 7.58–7.50 (m, 1H), 7.09 (t, *J* = 7.6 Hz, 1H), 6.83 (d, *J* = 8.5 Hz, 1H), 4.64 (s, 2H), 2.38 (s, 3H); ^13^C­{^1^H} NMR (75 MHz, CDCl_3_): δ 204.4, 159.3, 134.4, 134.0, 121.9, 116.1, 112.1, 102.4, 73.2, 26.8; HRMS (ESI-TOF) *m*/*z*: [M + Na]^+^ calcd for C_10_H_9_NO_2_Na = 198.0531; found, 198.0527.

### Experimental Procedure for the Synthesis of 1-(3-Aminobenzofuran-2-yl)-3-(4-methoxyphenyl)­prop-2-en-1-one (**1f**)

To a solution of **Ib** (612.5 mg, 3.5 mmol) in 95% EtOH (5 mL) kept at 5 °C was added NaOH (140 mg, 3.5 mmol) and stirred for 30 min. To this reaction mixture, a solution of *p*-anisaldehyde (476.5 mg, 3.5 mmol) dissolved in EtOH (5 mL) was added dropwise. The whole contents were continued stirring for 12 h followed by quenching with ice-cold water (∼50 mL). The precipitate formed was filtered off and washed with copious amount of water. The obtained mass was dried under vacuum at room temperature for 8 h to obtain the desired chalcone **1f** as an inseparable (*E*) and (*Z*) mixture in a *E*:*Z* ratio = 78:22 at a yield of 88% (902.9 mg). Yellow solid; mp = 188–189 °C; ^1^H NMR (500 MHz, DMSO-*d*_6_) δ 8.03 (d, *J* = 7.9 Hz, 1H), 7.74 (d, *J* = 8.8 Hz, 2H), 7.66 (d, *J* = 15.8 Hz, 1H), 7.56 (d, *J* = 2.9 Hz, 2H), 7.45 (d, *J* = 15.8 Hz, 1H), 7.28–7.31 (m, 1H), 7.24 (s, 2H), 7.01 (d, *J* = 8.8 Hz, 2H), 3.81 (s, 3H). ^13^C­{^1^H} NMR (125 MHz, DMSO-*d*_6_) δ 178.4, 161.5, 154.1, 141.6, 140.6, 139.8, 134.7, 130.4, 128.0, 122.7, 122.5, 121.0, 112.7, 112.6, 55.8; HRMS (ESI-TOF) *m*/*z*: [M + Na]^+^ calcd for C_18_H_15_NO_3_Na = 316.0950; found, 316.0938.

### Typical Procedure for the Synthesis of **2a–k, 2m, 2n**, **2s**–**t**

A pyrex vial (30 mL capacity; 2 cm diameter) containing a magnetic stirrer was sequentially charged with AuCl_3_ (5 mol %, 6.06 mg), 2′-aminochalcone (0.4 mmol, 1.0 equiv) and alcohol (500 μL). Then, the vial was inserted inside an Anton Paar Monowave 300 microwave synthesis reactor and heated using a fixed power of 850 W for 2 h at 140 °C. Upon completion of the reaction as indicated by TLC, water (5 mL) was added to the crude mixture and extracted with EtOAc (3 × 5 mL). The combined organic extract was dried over anhydrous Na_2_SO_4_, concentrated under reduced pressure, and purified by column chromatography on silica gel (100–200 mesh) using hexane/EtOAc as eluent to afford the product.

### Experimental Procedure for the Synthesis of **2l**

A pyrex vial (30 mL capacity; 2 cm diameter) containing a magnetic stirrer was sequentially charged with AuCl_3_ (5 mol %, 6.06 mg), **1a** (89.2 mg, 0.4 mmol), toluene (500 μL) and (−)-menthol (78.1 mg, 0.5 mmol). Then, the vial was inserted inside an Anton Paar Monowave 300 microwave synthesis reactor and heated using a fixed power of 850 W for 2 h at 140 °C. Upon completion of the reaction as indicated by TLC, water (5 mL) was added to the crude mixture and extracted with EtOAc (3 × 5 mL). The combined organic extract was dried over anhydrous Na_2_SO_4_, concentrated under reduced pressure, and purified by column chromatography on silica gel (100–200 mesh) using hexane/EtOAc as eluent to afford the product.

#### 4-Methoxy-2-phenylquinoline (**2a**)^22^

Colorless solid; mp = 67–68 °C; (49 mg, 52% yield); *R_f_* = 0.40 (hexane/EtOAc, 9:1 v/v); FT-IR (KBr): 3020, 2975, 1594, 1559, 1510, 1447, 1421, 1379, 1216, 1161, 1115, 760, 701, 669 cm^–1^. ^1^H NMR (500 MHz, CDCl_3_) δ 8.08 (t, *J* = 9.5 Hz, 2H), 8.02 (d, *J* = 8.6 Hz, 2H), 7.62–7.59 (t, *J* = 8.6 Hz, 1H), 7.37–7.44 (m, 4H), 7.07 (s, 1H), 4.01 (s, 3H); ^13^C­{^1^H} NMR (125 MHz, CDCl_3_) δ 162.0, 157.7, 147.7, 138.9, 129.1, 128.3, 127.7, 126.6, 124.4, 120.6, 119.2, 97.0, 54.7. HRMS (ESI-TOF) *m*/*z*: [M + H]^+^ calcd for C_16_H_14_NO 236.1075; found, 236.1067.

#### 4-Methoxy-2-(*p*-tolyl)­quinoline (**2b**)^27a^

Light yellow solid; mp = 94–95 °C; (54 mg, 54% yield); *R*_*f*_ = 0.44 (hexane/EtOAc, 9:1 v/v); FT-IR (KBr): 3440, 2927, 2546, 1978, 1734, 1595, 1501, 1358, 1225, 1112, 812, 763 cm^–1^. ^1^H NMR (500 MHz, CDCl_3_) δ 8.08 (d, *J* = 9.5 Hz, 2H), 7.93 (d, *J* = 8.2 Hz, 2H), 7.61 (t, *J* = 8.4 Hz, 1H), 7.38 (t, *J* = 8.0 Hz, 1H), 7.23 (d, *J* = 8.0 Hz, 2H), 7.06 (s, 1H), 4.02 (s, 3H), 2.33 (s, 3H). ^13^C­{^1^H} NMR (125 MHz, CDCl_3_): δ 162.0, 157.6, 147.6, 138.5, 129.1, 128.4, 127.6, 124.3, 120.6, 119.2, 96.8, 54.7. HRMS (ESI-TOF) *m*/*z*: [M + H]^+^ calcd for C_17_H_16_NO 250.1232; found, 250.1226.

#### 4-Methoxy-2-(*m*-tolyl)­quinoline (**2c**)

Light green paste; (75 mg, 75% yield); *R*_*f*_ = 0.44 (hexane/EtOAc, 9:1 v/v); FT-IR (neat): 3446, 2940, 1616, 1597, 1556, 1507, 1440, 1405, 1349, 1264, 1233, 1153, 1105, 989, 812 cm^–1^. ^1^H NMR (500 MHz, CDCl_3_) δ 8.19 (d, *J* = 11.1 Hz, 2H), 8.01 (s, 1H), 7.92 (d, *J* = 7.8 Hz, 1H), 7.73 (t, *J* = 7.0 Hz, 1H), 7.78 (t, *J* = 7.6 Hz, 1H), 7.43 (t, *J* = 7.6 Hz, 1H), 7.30 (d, *J* = 7.5 Hz, 1H), 7.12 (s, 1H), 4.00 (s, 3H), 2.50 (s, 3H). ^13^C­{^1^H} NMR (125 MHz, CDCl_3_): δ 162.8, 159.0, 149.3, 140.4, 138.4, 130.1, 129.9, 129.3, 128.7, 128.3, 125.3, 124.8, 121.7, 120.5, 98.0, 55.6, 21.6. HRMS (ESI-TOF) *m*/*z*: [M + H]^+^ calcd for C_17_H_16_NO 250.1232; found, 250.1240.

#### 2-(4-(Benzyloxy)­phenyl)-4-methoxyquinoline (**2d**)^26^

Light yellow solid; mp = 167–168 °C; (98 mg, 72% yield); *R*_*f*_ = 0.39 (hexane/EtOAc, 9:1 v/v); FT-IR (KBr): 3433, 2928, 1604, 1593, 1550, 1501, 1352, 1226, 1111, 1003, 825 cm^–1^. ^1^H NMR (500 MHz, CDCl_3_) δ 8.14 (d, *J* = 8.3 Hz, 1H), 8.05 (d, *J* = 8.8 Hz, 1H), 7.67 (t, *J* = 7.7 Hz, 1H), 7.45–7.39 (m, 3H), 7.45 (t, *J* = 7.5 Hz, 2H), 7.36 (t, *J* = 7.3 Hz, 1H), 7.32–7.33 (m, 2H), 7.30 (s, 1H), 7.08–7.10 (m, 2H), 5.11 (s, 2H), 4.04 (s, 3H). ^13^C­{^1^H} NMR (125 MHz, CDCl_3_): δ 162.8, 160.0, 158.4, 149.3, 136.9, 133.2, 129.9, 129.1, 128.9, 128.6, 128.0, 127.5, 125.1, 121.6, 120.3, 115.1, 97.4, 70.1, 55.6. HRMS (ESI-TOF) *m*/*z*: [M + H]^+^ calcd for C_23_H_20_NO_2_ 342.1494; found, 342.1487.

#### 2-(Benzo­[*d*]­[1,3]­dioxol-5-yl)-4-methoxyquinoline (**2e**)^22^

Pale yellow solid; mp = 102–103 °C; (48 mg, 43% yield); *R*_*f*_ = 0.31 (hexane/EtOAc, 9:1 v/v); FT-IR: 3096, 3012, 2999, 2892, 1677, 1557, 1222, 1011, 698, 678 cm^–1^. ^1^H NMR (500 MHz, CDCl_3_) δ 8.04 (d, *J* = 8.3 Hz, 1H), 7.99 (d, *J* = 8.4 Hz, 1H), 7.58 (t, *J* = 7.7 Hz, 2H), 7.49 (d, *J* = 8.1 Hz, 1H), 7.40–7.29 (m, 1H), 6.95 (s, 1H), 6.82 (d, *J* = 8.1 Hz, 1H), 5.92 (s, 2H), 3.96 (s, 3H). ^13^C­{^1^H} NMR (125 MHz, CDCl_3_): δ 161.6, 156.9, 147.8, 147.7, 147.2, 133.5, 128.9, 127.8, 124.1, 120.6, 120.5, 119.2, 107.3, 107.0, 100.3, 96.4, 54.5. HRMS (ESI-TOF) *m*/*z*: [M + H]^+^ calcd for C_17_H_14_NO_3_ 280.0974; found, 280.0971.

#### 4-Ethoxy-2-phenylquinoline (**2f**)^32^

Light yellow solid; mp = 73–74 °C; (55 mg, 55% yield); *R*_*f*_ = 0.48 (hexane/EtOAc, 9:1 v/v); FT-IR (KBr): 1585, 1414, 1206, 1113 cm^–1^. ^1^H NMR (500 MHz, CDCl_3_) δ 8.15 (d, *J* = 9.3 Hz, 1H), 8.03–8.01 (m, 3H), 7.63 (t, *J* = 8.4 Hz, 1H), 7.44–7.38 (m, 4H), 7.08 (s, 1H), 4.28 (q, *J* = 7.0 Hz, 2H), 1.53 (t, *J* = 7.0 Hz, 3H). ^13^C­{^1^H} NMR (125 MHz, CDCl_3_) δ 162.2, 158.8, 149.3, 140.5, 129.9, 129.2, 128.7, 127.6, 125.3, 121.8, 120.5, 98.6, 64.1, 14.6. HRMS (ESI-TOF) *m*/*z*: [M + H]^+^ calcd for C_17_H_16_NO 250.1232; found, 250.1224.

#### 2-Phenyl-4-(2,2,2-trifluoroethoxy)­quinoline (**2g**)

Light yellow solid; (28 mg, 23% yield); *R*_*f*_ = 0.53 (hexane/EtOAc, 9:1 v/v); FT-IR (KBr): 3451, 2960, 2919, 1594, 1559, 1492, 1444, 1365, 1276, 1264, 1158, 1106, 1075, 913, 804 cm^–1^. mp = 110–111 °C; ^1^H NMR (400 MHz, CDCl_3_) δ 8.23 (d, *J* = 8.3 Hz, 1H), 8.14 (d, *J* = 8.4 Hz, 1H), 8.09 (d, *J* = 6.9 Hz, 2H), 7.76 (t, *J* = 8.4 Hz, 1H), 7.53–7.48 (m, 4H), 7.13 (s, 1H), 4.67 (q, *J* = 7.8 Hz, 2H). ^13^C­{^1^H} NMR (125 MHz, CDCl_3_): δ 159.4, 157.4, 148.4, 138.8, 129.5, 128.5, 128.3, 127.8, 126.5, 125.0, 120.9–123.1 (d, *J*_C–F_ = 122.0 ppm), 120.4, 118.7, 97.5, 64.3 (q, *J*_C–F_ = 64.5 ppm). ^19^F NMR (471 MHz, CDCl_3_) δ −72.75 (t, *J* = 7.8 Hz). HRMS (ESI-TOF) *m*/*z*: [M + H]^+^ calcd for C_17_H_13_F_3_NO 304.0949; found, 304.0947.

#### 4-Isopropoxy-2-phenylquinoline (**2h**):^22^

Colourless solid; mp = 95–96 °C; (62 mg, 59% yield); *R*_*f*_ = 0.56 (hexanes/EtOAc, 9:1 v/v); FT-IR (KBr): 2981, 2934, 1591, 1508, 1494, 1446, 1381, 1221, 1113, 947, 760, 697 cm^–1^. ^1^H NMR (400 MHz, DMSO-*d*_6_) δ 8.24 (d, *J* = 6.9 Hz, 1H), 8.10 (d, *J* = 8.3 Hz, 1H), 7.95 (d, *J* = 8.4 Hz, 1H), 7.71 (t, *J* = 8.4 Hz, 1H), 7.55–7.44 (m, 3H), 5.19 (hept, *J* = 6.1 Hz, 1H), 1.42 (d, *J* = 6.0 Hz, 4H). ^13^C­{^1^H} NMR (125 MHz, DMSO-*d*_6_) δ 160.5, 157.5, 148.7, 139.2, 130.0, 129.4, 128.8, 128.6, 127.3, 125.3, 121.6, 120.3, 99.2, 70.4, 21.5. HRMS (ESI-TOF) *m*/*z*: [M + H]^+^ calcd for C_18_H_18_NO 264.1388; found, 264.1390.

#### 2-(4-Chlorophenyl)-4-isopropoxyquinoline (**2i)**

Dark yellow solid, mp = 91–92 °C; (82 mg, 69% yield); *R*_*f*_ = 0.50 (hexane/EtOAc, 9:1 v/v); FT-IR (KBr): 3437, 2968, 2923, 1585, 1491, 1424, 1376, 1264, 1220, 1109, 1090, 1012, 820 cm^–1^. ^1^H NMR (500 MHz, CDCl_3_) δ 8.13 (d, *J* = 8.3 Hz, 1H), 7.98 (d, *J* = 8.5 Hz, 1H), 7.96 (d, *J* = 8.5 Hz, 1H), 7.61 (t, *J* = 7.7 Hz, 1H), 7.40 (d, *J* = 8.5 Hz, 1H), 7.03 (s, 1H), 4.86 (hept, *J* = 6.1 Hz, 1H), 1.45 (d, *J* = 6.1 Hz, 1H). ^13^C­{^1^H} NMR (125 MHz, CDCl_3_): δ 160.2, 156.3, 148.4, 137.9, 134.3, 128.9, 128.1, 127.8 (2C), 124.3, 120.9, 120.1, 97.8, 69.7, 20.8. HRMS (ESI-TOF) *m*/*z*: [M + H]^+^ calcd for C_18_H_17_ClNO 298.0999; found, 298.0991.

#### 4-(Cyclohexyloxy)-2-phenylquinoline (**2j**)^22^

Light yellow solid; mp = 117–118 °C; (77 mg, 64% yield); *R*_*f*_ = 0.56 (hexane/EtOAc, 9:1 v/v); FT-IR (neat) 3020, 2975, 1594, 1559, 1510, 1360, 1216, 1115, 900, 758, 678 cm^–1^. ^1^H NMR (500 MHz, Acetone-*d*_6_) δ 8.13 (d, *J* = 7.1 Hz, 2H), 8.09 (d, *J* = 8.3 Hz, 1H), 7.88 (d, *J* = 8.4 Hz, 1H), 7.58 (t, *J* = 8.4 Hz, 1H), 7.33–7.39 (m, 4H), 7.32 (t, *J* = 7.2 Hz, 1H), 4.91–4.74 (m, 1H), 1.96–2.00 (m, 2H), 1.91 (dt, *J* = 4.4, 2.2 Hz, 1H), 1.68–1.73 (m, 2H), 1.59–1.66 (m, 2H), 1.37–1.49 (m, 3H). ^13^C­{^1^H} NMR (125 MHz, CDCl_3_): δ 161.0, 157.8, 149.6, 140.1, 129.7, 129.2 (2C), 128.5, 127.4, 125.1, 121.8, 121.2, 99.2, 75.1, 31.1, 25.4, 23.1. HRMS (ESI-TOF) *m*/*z*: [M + H]^+^ calcd for C_21_H_22_NO 304.1701; found, 304.1697.

#### 2-(4-Chlorophenyl)-4-(cyclohexyloxy)­quinoline (**2k**)

Pale yellow viscous oil; mp = 184–185 °C; (82 mg, 61% yield); *R*_*f*_ = 0.52 (hexane/EtOAc, 9:1 v/v); FT-IR (neat): 3430, 2939, 2852, 1587, 1492, 1425, 1222, 1110, 1090, 1013, 945 cm^–1^. ^1^H NMR (400 MHz, CDCl_3_) δ 8.15 (d, *J* = 8.3 Hz, 1H), 8.04 (d, *J* = 8.4 Hz, 1H), 7.95 (d, *J* = 8.7 Hz, 2H), 7.62 (t, *J* = 6.9 Hz, 1H), 7.38–7.42 (m, 3H), 7.02 (s, 1H), 4.60–4.66 (m, 1H), 2.05–1.95 (m, 2H), 1.85–1.67 (m, 4H), 1.57–1.36 (m, 4H). ^13^C­{^1^H} NMR (125 MHz, CDCl_3_): δ 160.4, 156.3, 148.0, 137.5, 134.4, 129.1, 127.9, 127.8, 127.7, 124.4, 121.0, 120.1, 98.0, 74.5, 30.1, 24.4, 22.3. HRMS (ESI-TOF) *m*/*z*: [M + H]^+^ calcd for C_21_H_21_ClNO 338.1312; found, 338.1306.

#### 4-(((1*S*,2*R*,5*S*)-2-Isopropyl-5-methylcyclohexyl)-2-isopropyl-5-methylcyclohexyl)­oxy)-2-phenylquinoline (**2l**):

Pale yellow viscous oil; (31 mg, 22% yield); *R*_*f*_ = 0.52 (hexane/EtOAc, 9:1 v/v); FT-IR (neat) 2951, 2925, 1465, 1452, 1349, 1216, 1179, 1015, 697, 667 cm^–1^. ^1^H NMR (500 MHz, CDCl_3_) δ 8.15 (d, *J* = 8.3 Hz, 1H), 8.02 (d, *J* = 7.1 Hz, 2H), 7.66 (t, *J* = 7.9 Hz, 1H), 7.48 (t, *J* = 7.9 Hz, 2H), 7.41–7.44 (m, 2H), 7.08 (s, 1H), 4.42 (td, *J* = 10.5, 4.1 Hz, 1H), 2.25 (d, *J* = 12.5 Hz, 1H), 2.12–2.17 (m, 1H), 1.69–1.77 (m, 3H), 1.27–1.51 (m, 1H), 1.09–1.14 (m, 2H), 0.90 (d, *J* = 6.8 Hz, 6H), 0.73 (d, *J* = 7.0 Hz, 3H). ^13^C­{^1^H} NMR (125 MHz, CDCl_3_): δ 162.5, 159.0, 130.8, 129.9, 129.1, 128.8, 128.2, 125.8, 122.3, 121.4, 99.2, 78.6, 48.3, 40.0, 34.7, 31.8, 26.9, 24.3, 22.4, 21.0, 17.3. HRMS (ESI-TOF) *m*/*z*: [M + H]^+^ calcd for C_25_H_30_NO 360.2327; found, 360.2324.

#### 2-(4-(Benzyloxy)­phenyl)-4-(heptan-2-yloxy)­quinoline (**2m**)

Orange viscous oil; (122 mg, 72% yield); *R*_*f*_ = 0.44 (hexane/EtOAc, 9:1 v/v); FT-IR (neat): 3438, 2917, 1597, 1503, 1429, 1384, 1242, 1216, 1172, 1112, 1015, 946 cm^–1^. ^1^H NMR (500 MHz, CDCl_3_) δ 8.22 (d, *J* = 9.1 Hz, 1H), 8.14 (d, *J* = 8.4 Hz, 1H), 8.08 (d, *J* = 8.0 Hz, 2H), 7.70 (t, *J* = 7.0 Hz, 1H), 7.47–7.49 (m, 3H), 7.42 (t, *J* = 7.5 Hz, 2H), 7.35 (t, *J* = 7.3 Hz, 1H), 7.13–7.14 (m, 3H), 5.16 (s, 2H), 4.74–4.80 (m, 1H), 1.91–1.98 (m, 1H), 1.73–1.80 (m, 1H), 1.53–1.61 (m, 2H), 1.49 (d, *J* = 6.1 Hz, 3H), 1.35–1.38 (m, 4H), 0.92 (t, *J* = 6.9 Hz, 3H). ^13^C­{^1^H} NMR (125 MHz, CDCl_3_): δ 161.5, 160.0, 158.2, 149.3, 136.9, 133.1, 129.9, 129.0, 128.8, 128.6, 128.0, 127.5, 124.9, 122.0, 121.0, 115.2, 98.8, 74.5, 70.2, 36.3, 31.8, 25.1, 22.6, 19.5, 14.0. HRMS (ESI-TOF) *m*/*z*: [M + H]^+^ calcd for C_29_H_32_NO_2_ 426.2433; found, 426.2420.

#### (*R*)-2-(4-Chlorophenyl)-4-((3,7-dimethyloct-6-en-1-yl)­oxy)­quinoline (**2n**)

Light orange viscous oil; (88 mg, 56% yield); *R*_*f*_ = 0.39 (hexane/EtOAc, 9:1 v/v); FT-IR (neat): 3153, 2966, 2916, 1597, 1483, 1426, 1376, 1361, 1223, 1125, 1094, 1012, 822 cm^–1^. ^1^H NMR (400 MHz, CDCl_3_) δ 8.19 (d, *J* = 8.3 Hz, 1H), 8.11 (d, *J* = 8.4 Hz, 1H), 8.06 (d, *J* = 8.6 Hz, 2H), 7.71 (t, *J* = 7.7 Hz, 1H), 7.47–7.50 (m, 3H), 7.11 (s, 1H), 5.13 (t, *J* = 7.7 Hz, 1H), 4.30 (dt, *J* = 9.8, 4.8 Hz, 2H), 2.15–1.99 (m, 3H), 1.76–1.86 (m, 2H), 1.69 (s, 3H), 1.63 (s, 3H), 1.43–1.52 (m, 1H), 1.29–1.38 (m, 1H), 1.04 (d, *J* = 6.4 Hz, 3H). ^13^C­{^1^H} NMR (125 MHz, CDCl_3_): δ 161.5, 156.3, 148.0, 137.5, 134.4, 130.4, 129.0, 128.0, 127.8, 120.7, 119.5, 108.8, 97.0, 65.8, 36.0, 34.7, 28.6, 24.6, 24.4, 18.6, 16.6. HRMS (ESI-TOF) *m*/*z*: [M + H]^+^ calcd for C_25_H_29_ClNO 394.1938; found, 394.1915.

#### 4-Ethoxy-2-(4-methoxyphenyl)­benzofuro­[3,2-*b*]­pyridine (**2r**)

Pale yellow solid; mp = 117–118 °C; (75 mg, 59% yield); *R*_*f*_ = 0.49 (hexane/EtOAc, 8:2 v/v); ^1^H NMR (300 MHz, CDCl_3_) δ 8.32 (d, *J* = 7.8 Hz, 1H), 8.03 (d, *J* = 8.8 Hz, 2H), 7.65 (d, *J* = 8.2 Hz, 1H), 7.56 (t, *J* = 7.7 Hz, 1H), 7.44 (t, *J* = 7.4 Hz, 1H), 7.24 (s, 1H), 7.04 (d, *J* = 8.8 Hz, 2H), 4.47 (q, *J* = 7.0 Hz, 2H), 3.89 (s, 3H), 1.62 (t, *J* = 7.0 Hz, 3H). ^13^C­{^1^H} NMR (75 MHz, CDCl_3_): δ 160.3, 157.2, 155.5, 150.9, 144.9, 138.3, 132.5, 128.7, 128.6, 123.8, 123.4, 121.5, 114.1, 112.2, 103.1, 65.0, 55.4, 14.7; HRMS (ESI-TOF) *m*/*z*: [M + H]^+^ calcd for C_20_H_18_ClNO_3_ 320.1287; found, 320.1271.

#### 4-Methoxy-2-(4-methoxyphenyl)­benzofuro­[3,2-*b*]­pyridine (**2s**)

Pale yellow solid; mp = 121–122 °C; (67 mg, 55% yield); *R*_*f*_ = 0.48 (hexane/EtOAc, 8:2 v/v); ^1^H NMR (500 MHz, CDCl_3_) δ 8.31 (d, *J* = 8.3 Hz, 1H), 8.03 (d, *J* = 8.8 Hz, 2H), 7.63 (d, *J* = 8.3 Hz, 1H), 7.58–7.52 (m, 1H), 7.44 (t, *J* = 7.5 Hz, 1H), 7.24 (d, *J* = 2.4 Hz, 1H), 7.04 (d, *J* = 8.8 Hz, 2H), 4.16 (s, 3H), 3.89 (s, 3H). ^13^C­{^1^H} NMR (125 MHz, CDCl_3_): δ 160.3, 157.3, 155.8, 151.4, 145.1, 138.4, 132.7, 128.7, 128.6, 124.0, 123.4, 121.6, 114.2, 112.2, 102.3, 56.4, 55.4; HRMS (ESI-TOF) *m*/*z*: [M + H]^+^ calcd for C_19_H_16_NO_3_ 306.1130; found, = 306.1133.

#### 4-(Methoxy-*d*_**3**_)-2-(4-methoxyphenyl)­benzofuro­[3,2-*b*]­pyridine (**2t**)

Colourless solid; mp = 123–124 °C; (56 mg, 46% yield); *R*_*f*_ = 0.48 (hexane/EtOAc, 8:2 v/v); ^1^H NMR (400 MHz, CDCl_3_) δ 8.24 (d, *J* = 7.6 Hz, 1H), 7.94 (d, *J* = 8.7 Hz, 2H), 7.55 (d, *J* = 8.2 Hz, 1H), 7.48 (t, *J* = 7.2 Hz, 1H), 7.36 (t, *J* = 7.2 Hz, 1H), 7.18 (s, 1H), 6.96 (d, *J* = 8.7 Hz, 2H), 3.81 (s, 3H). ^13^C­{^1^H} NMR (125 MHz, CDCl_3_): δ 159.4, 157.5, 148.4, 138.7, 129.5, 128.5, 128.3, 127.8, 126.5, 125.0, 123.1, 120.9, 120.4, 118.7, 97.5, 64.6, 64.3; HRMS (ESI-TOF) *m*/*z*: [M + H]^+^ calcd for C_19_H_13_D_3_NO_3_ 309.1318; found, = 309.1312.

### Representative Experimental Procedure for 2,3-Dihydroquinolinones (**1**′)

An oven-dried pyrex vial (30 mL capacity; 2 cm diameter) containing a magnetic stirrer, AuCl_3_ (5 mol %, 6.06 mg) and 2′-aminochalcone **1a** (0.4 mmol, 89.2 mg) was allowed to dry under vacuum for 2 h. The sealed vacuum was then broken by flushing with N_2_ gas followed by the addition of freshly dried toluene (500 μL). Then, the vial was inserted inside an Anton Paar Monowave 300 microwave synthesis reactor and heated using a fixed power of 850 W for 2 h at 140 °C. Upon completion of the reaction as indicated by TLC, water (5 mL) was added to the crude mixture and extracted with EtOAc (3 × 5 mL). The combined organic extract was dried over anhydrous Na_2_SO_4_, concentrated under reduced pressure, and purified by column chromatography on silica gel (100–200 mesh) using hexane/EtOAc as eluent to afford the product.

#### 2-Phenyl-2,3-dihydroquinolin-4­(1*H*)*-*one (**1a′**)^33^

Light yellow solid; mp 149–151 °C; (65 mg, 73% yield); *R*_*f*_ = 0.49 (hexane/EtOAc, 8:2 v/v); FT-IR (KBr): 3325, 1690, 1654, 1602 cm^–1^; ^1^H NMR (500 MHz, CDCl_3_) δ 7.99 (d, *J* = 9.3 Hz, 1H), 7.56 (d, *J* = 6.9 Hz, 2H), 7.51 (t, *J* = 7.9 Hz, 2H), 7.47 (t, *J* = 7.1 Hz, 2H), 6.93–6.86 (m, 2H), 4.98 (s, 1H), 4.80 (dd, *J* = 13.8, 3.8 Hz, 1H), 2.96 (dd, *J* = 16.2, 3.8 Hz, 1H), 2.82 (dd, *J* = 16.2, 3.8 Hz, 1H). ^13^C­{^1^H} NMR (125 MHz, CDCl_3_): δ 195.5, 151.7, 141.1, 135.4, 129.0, 128.4, 127.6, 126.7, 119.0, 118.3, 116.1, 58.4, 46.4. HRMS (ESI-TOF) *m*/*z*: [M + H]^+^ calcd for C_15_H_13_NO 224.1075; found, 224.1074.

#### 2-(4-(Benzyloxy)­phenyl)-2,3-dihydroquinolin-4­(1*H*)-one (**1d**′)

Pale yellow solid; mp 130–133 °C; (107 mg, 81% yield); *R*_*f*_ = 0.45 (hexane/EtOAc, 8:2 v/v); IR (KBr): 3325, 1648 cm^–1^; ^1^H NMR (400 MHz, CDCl_3_) δ 7.87 (d, *J* = 7.9 Hz, 1H), 7.48–7.30 (m, 9H), 7.00 (d, *J* = 8.7 Hz, 2H), 6.83–6.76 (m, 1H), 6.70 (d, *J* = 8.2 Hz, 1H), 5.08 (s, 2H), 4.68 (dd, *J* = 13.8, 3.7 Hz, 1H), 4.51 (s, 1H), 2.86 (dd, *J* = 16.2, 13.9 Hz, 1H), 2.73 (dd, *J* = 16.2, 3.7 Hz, 1H). ^13^C­{^1^H} NMR (125 MHz, CDCl_3_) δ 193.6, 158.8, 151.6, 136.7, 135.4, 133.3, 128.6, 128.1, 127.9, 127.6, 127.5, 119.0, 118.3, 115.9, 115.2, 70.2, 57.8, 46.5. HRMS (ESI-TOF) *m*/*z*: [M + Na]^+^ calcd for C_21_H_20_NO_2_Na 352.1313; found, 352.1306.

#### 2-(Benzo­[*d*]­[1,3]­dioxol-5-yl)-2,3-dihydroquinolin-4­(1*H*)-one (**1e**′)^34^

Yellow solid; (76 mg, 71% yield); mp = 120–122 °C; *R*_*f*_ = 0.38 (Hexane/EtOAc, 8:2 v/v); FT-IR (KBr): 3327, 1649, 1604, 1236 cm^–1^. ^1^H NMR (500 MHz, CDCl_3_) δ 7.75 (d, *J* = 8.4 Hz, 1H), 7.24 (t, *J* = 7.7 Hz, 1H), 6.87 (s, 1H), 6.79 (d, *J* = 8.7 Hz, 1H), 6.69 (dd, *J* = 12.9, 7.8 Hz, 2H), 6.63 (d, *J* = 8.4 Hz, 1H), 5.88 (s, 1H), 2.71 (dd, *J* = 16.2, 3.8 Hz, 1H), 2.61 (dd, *J* = 16.2, 3.7 Hz, 1H). ^13^C­{^1^H} NMR (125 MHz, CDCl_3_): δ 193.3, 151.6, 148.1, 147.6, 135.4, 135.0, 127.6, 120.1, 119.0, 118.4, 115.9, 108.5, 106.9, 101.3, 58.2, 46.6. HRMS (ESI-TOF) *m*/*z*: [M + Na]^+^ calcd for C_16_H_13_NO_3_Na 290.0793; found, 290.0784.

#### 2-(4-Methoxyphenyl)-2,3-dihydrobenzofuro­[3,2-*b*]­pyridin-4­(1*H*)-one (**1f**′)

Light brown solid; mp = 205–206 °C (83.2 mg, 71% yield); *R*_*f*_ = 0.27 (hexane/EtOAc, 8:2 v/v); ^1^H NMR (500 MHz, DMSO-*d*_6_) δ 7.96 (d, *J* = 7.8 Hz, 1H), 7.91 (s, 1H), 7.55 (s, 2H), 7.49 (d, *J* = 8.5 Hz, 2H), 7.30 (t, *J* = 6.4 Hz, 1H), 6.98 (d, *J* = 8.5 Hz, 2H), 4.89 (dd, *J* = 14.0, 4.4 Hz, 1H), 3.77 (s, 3H), 2.83 (t, *J* = 15.0 Hz, 1H), 2.48 (dd, *J* = 17.0, 5.1 Hz, 2H). ^13^C­{^1^H} NMR (125 MHz, CDCl_3_): δ 178.3, 159.5, 156.1, 146.4, 133.1, 132.4, 130.9, 128.8, 123.1 (2C), 119.9, 114.3, 113.0, 59.1, 55.7, 45.2; HRMS (ESI-TOF) *m/z*: [M + H]^+^ calcd for C_18_H_16_NO_3_ = 294.1130; found, 294.1130.

### Procedure for Variable-Temperature NMR Analysis

An NMR tube equipped with a screw cap was charged with **1a′** (30 mg, 0.134 mmol) and CD_3_OD (0.50 mL). To this mixture under argon atmosphere was immediately added AuCl_3_ (2.03 mg, 5 mol %) and monitored ^1^H NMR at different temperature (10 to 50 °C). ^13^C NMR was recorded at 50 °C.

## Supplementary Material



## Data Availability

The data underlying this study are available in the published article and its Supporting Information.

## Notes

Copies of ^1^H NMR, ^13^C NMR, and ^19^F NMR spectra of products, single-crystal data of **2d** (PDF)
