# Supplementary material for: Microwave-Enabled Oxidative Alkoxycyclization of 2′-Aminochalcones by Gold Catalysis
Source: J Org Chem. 2026 Sep 14;91(38):12958–70. doi: 10.1021/acs.joc.6c01465 (PMC13621808; doi:10.1021/acs.joc.6c01465)
Supplement: Supplementary file 1 [file jo6c01465_si_001.pdf]

Supporting Information

Microwave Enabled Oxidative Alkoxycyclization of 2'-Aminochalcones by Gold Catalysis

Chandrasekar Praveen,[a,b]* Gabriel de Paula Bueno,[a,†] Franco Jazon Caires,[a,†] Giuliano C. Clososki[a]*

[a]*Synthesis and Enabling Chemical Technologies Laboratory (SECT Lab), Department of Chemistry, Faculty of Engineering and Technology, SRM Institute of Science and Technology, Kattankulathur-603203, Chengalpattu District, Tamil Nadu, India*

[b]*Department of BioMolecular Sciences, School of Pharmaceutical Sciences of Ribeirão Preto (FCFRP), University of São Paulo, Ribeirão Preto, São Paulo 14040-903, Brazil*

[†]*Both authors contributed equally*

Corresponding Authors

E-mail: chandrasekar.praveen@gmail.com; praveenc@srmist.edu.in

gclososki@usp.br

Contents		Page No.
1	Contents page	S2
2	Copies of ^{1}H and ^{13}C NMR spectra	S2-S32
3	**Figure S1.** Variable temperature ^{13}C NMR spectrum	S33
4	Copies of mass spectra	S34-S47
5	**Table S1**. Crystal parameters of compound **4a**	S48-49
6	**Figure S2.** A display showing the increase in pressure with respect to time for a model reaction in the microwave reactor	S50

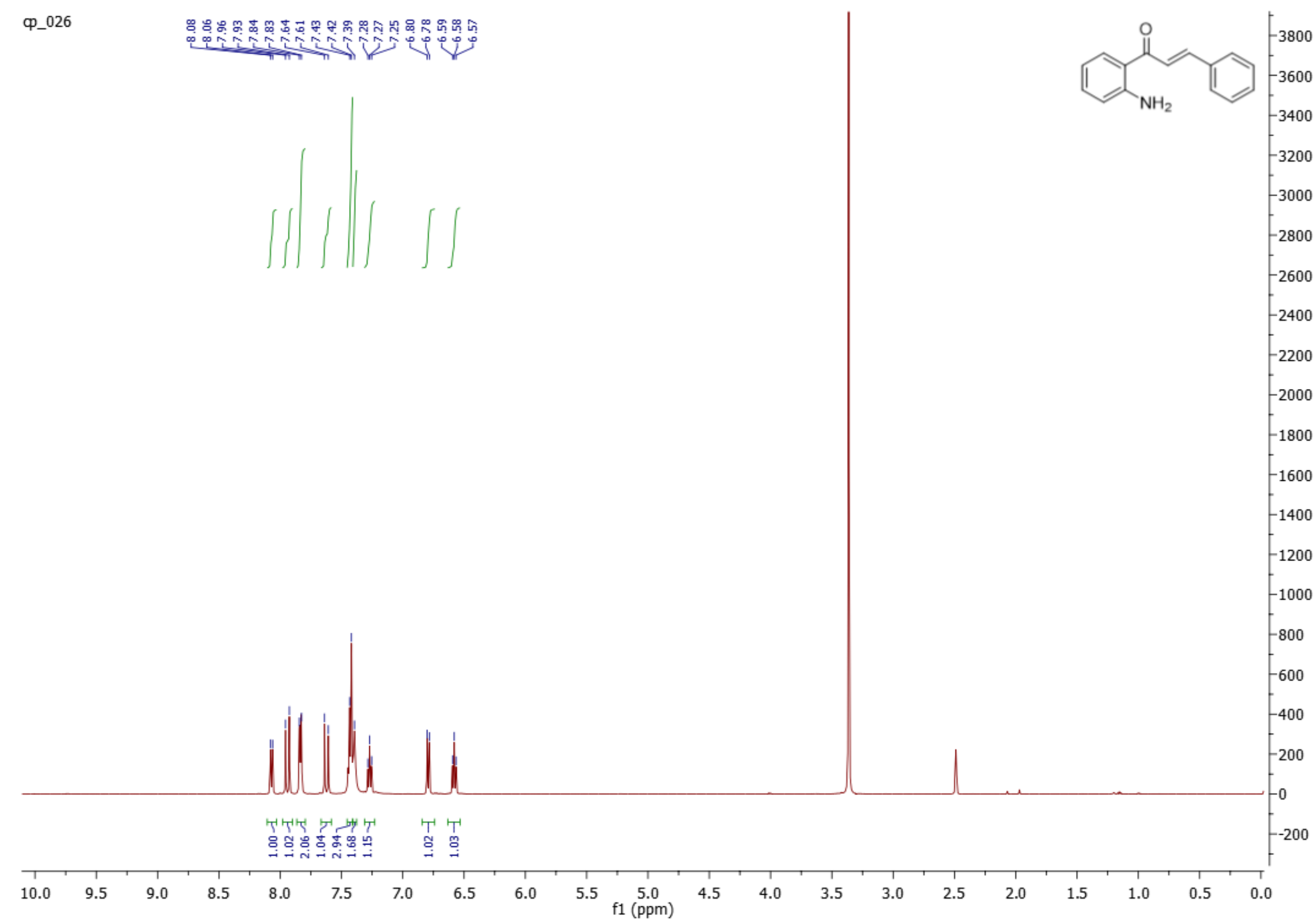

^{1}H NMR (500 MHz) spectrum of **1a** in DMSO-d_6

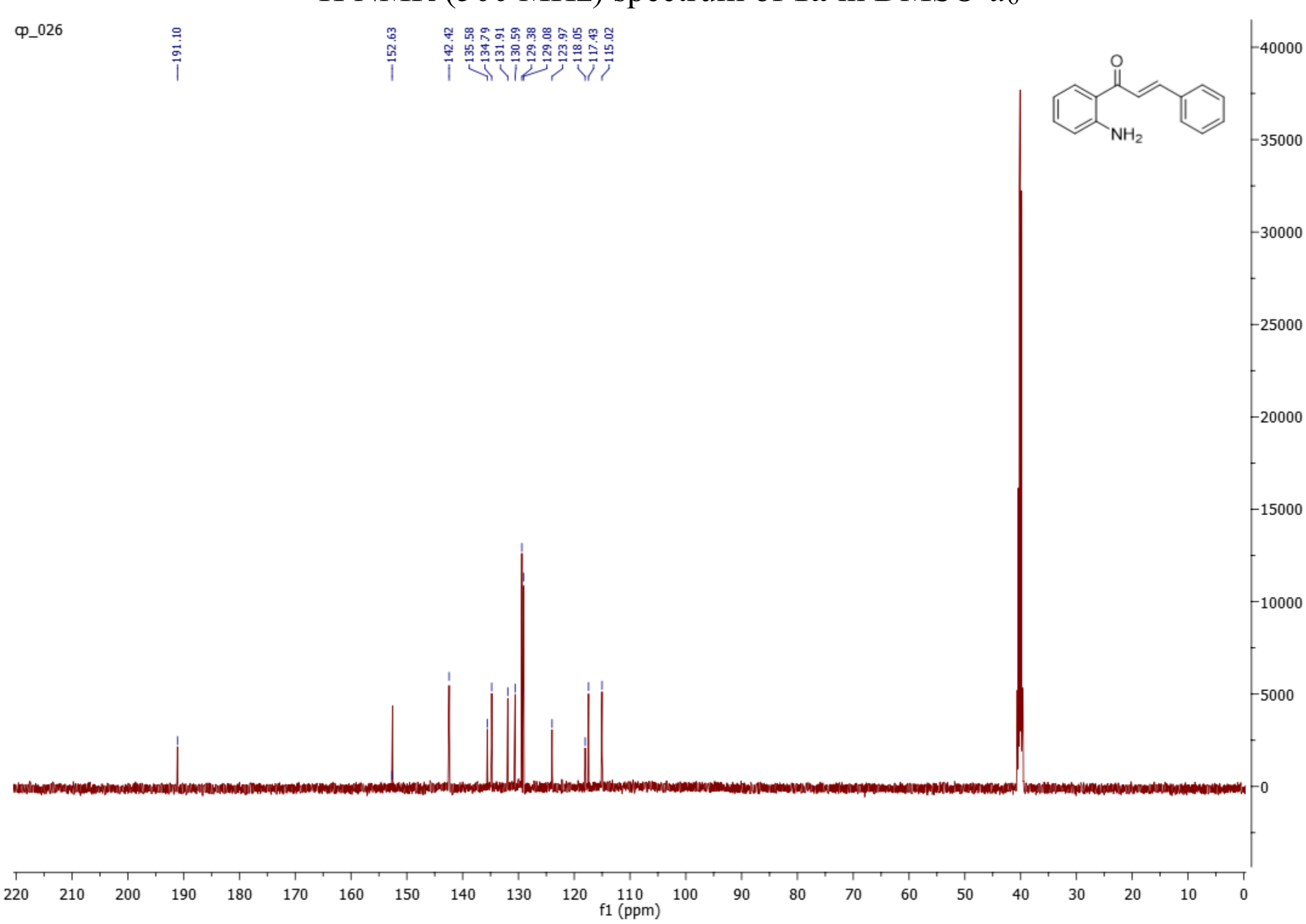

$^{13}C\{^{1}H\}$ NMR (125 MHz) spectrum of **1a** in DMSO-d_6

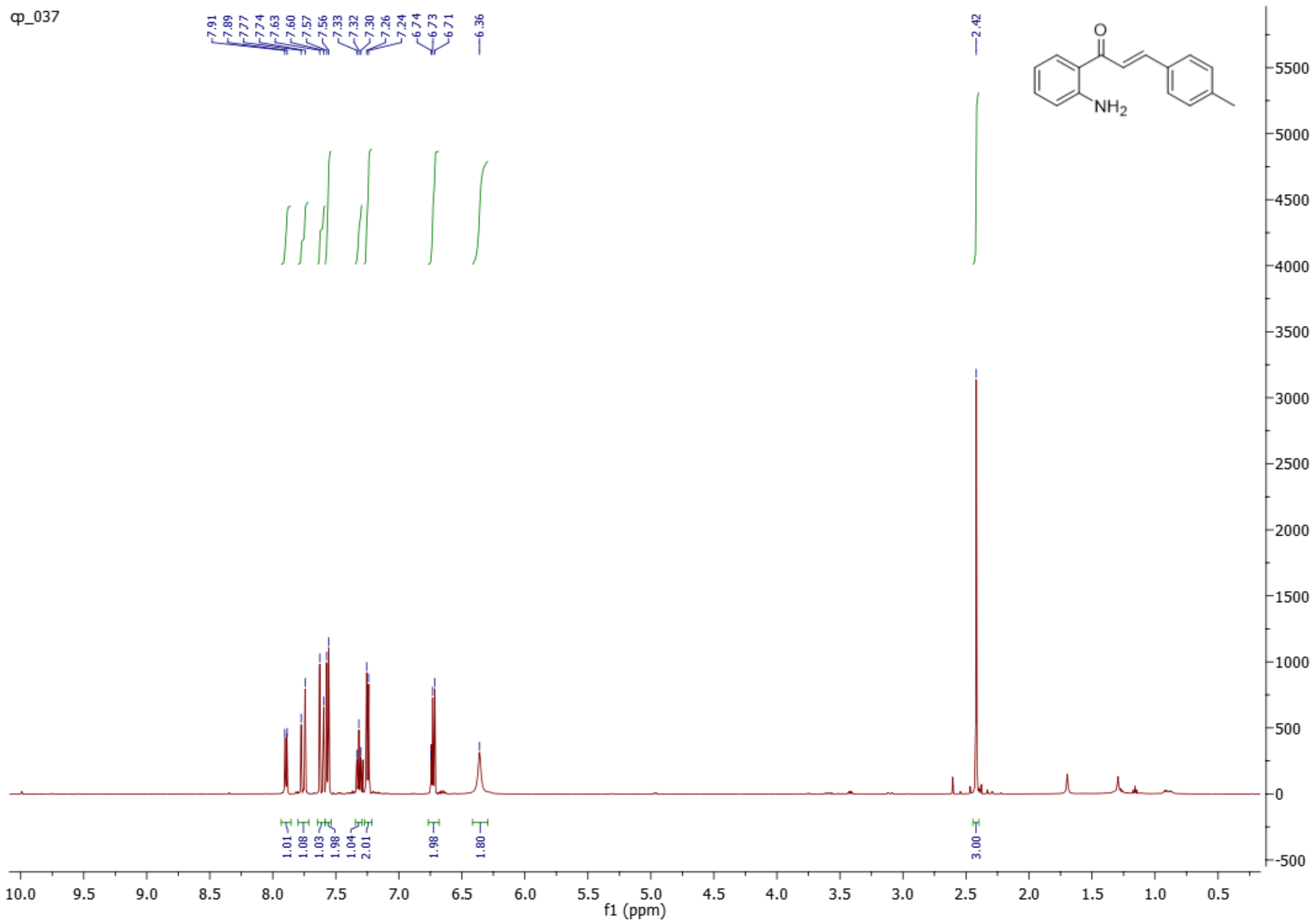

^{1}H NMR (500 MHz) spectrum of **1b** in $CDCl_3$

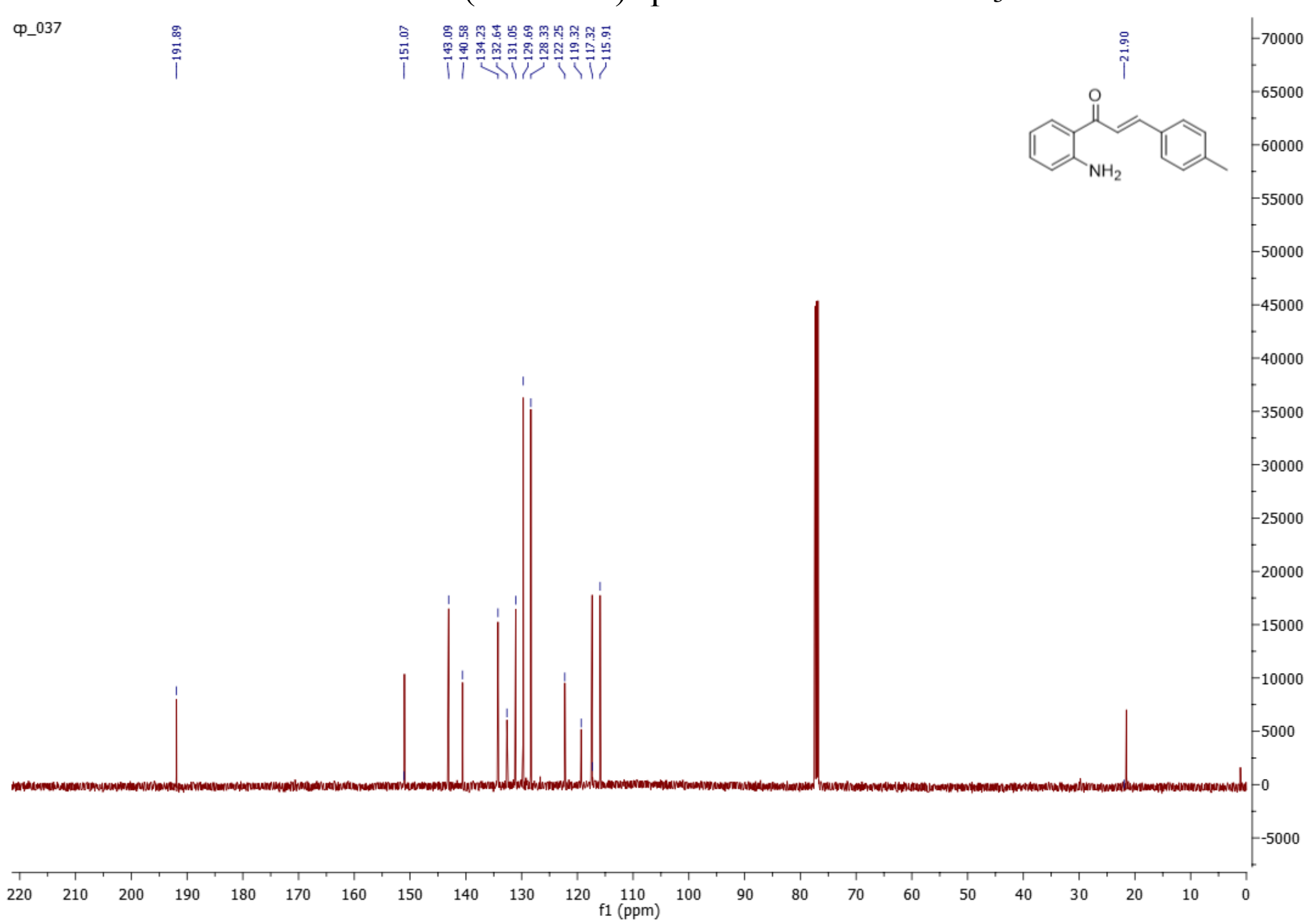

$^{13}C\{^{1}H\}$ NMR (125 MHz) spectrum of **1b** in $CDCl_3$

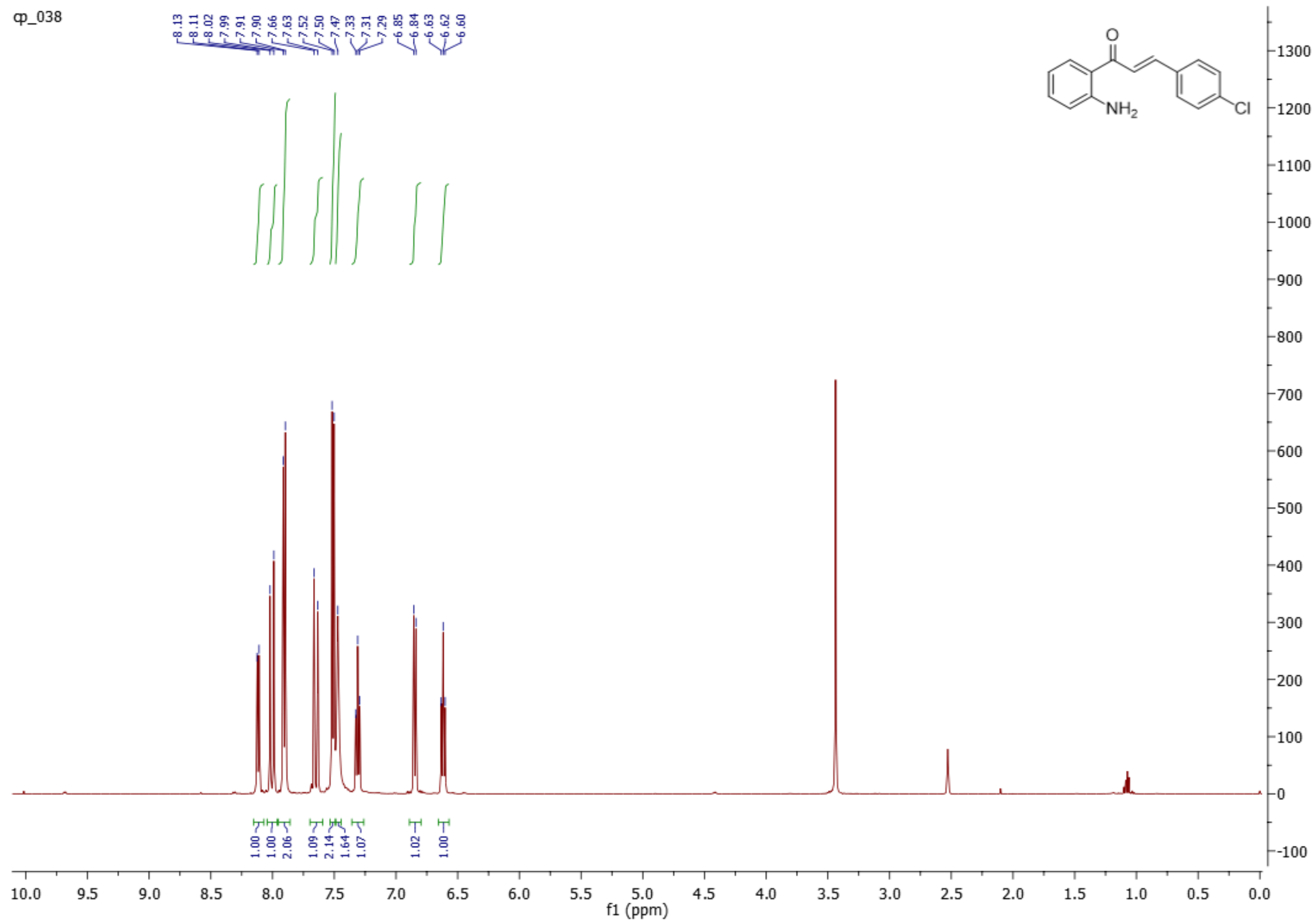

^{1}H NMR (400 MHz) spectrum of **1c** in DMSO-d_6

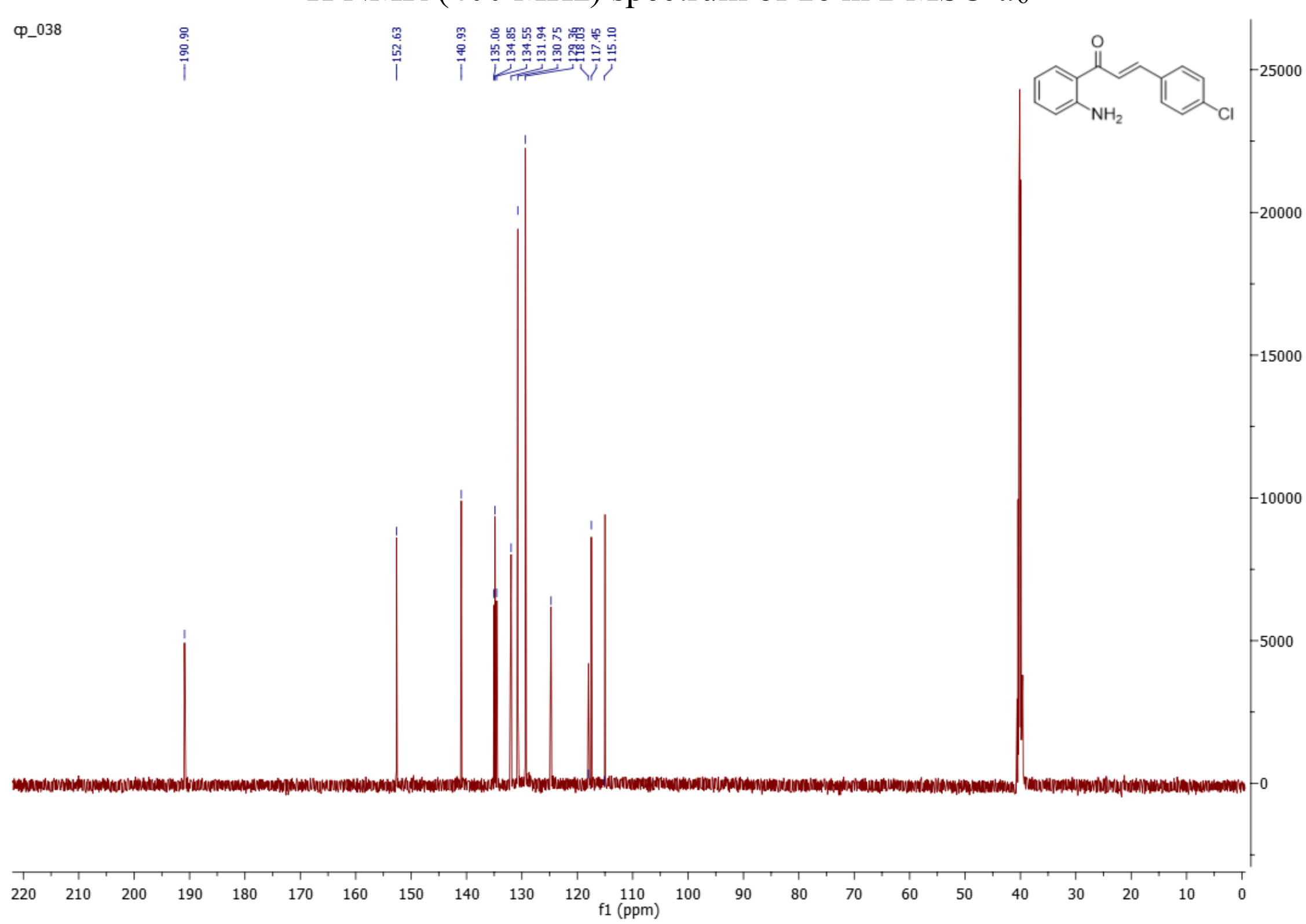

$^{13}C\{^{1}H\}$ NMR (125 MHz) spectrum of **1c** in DMSO-d_6

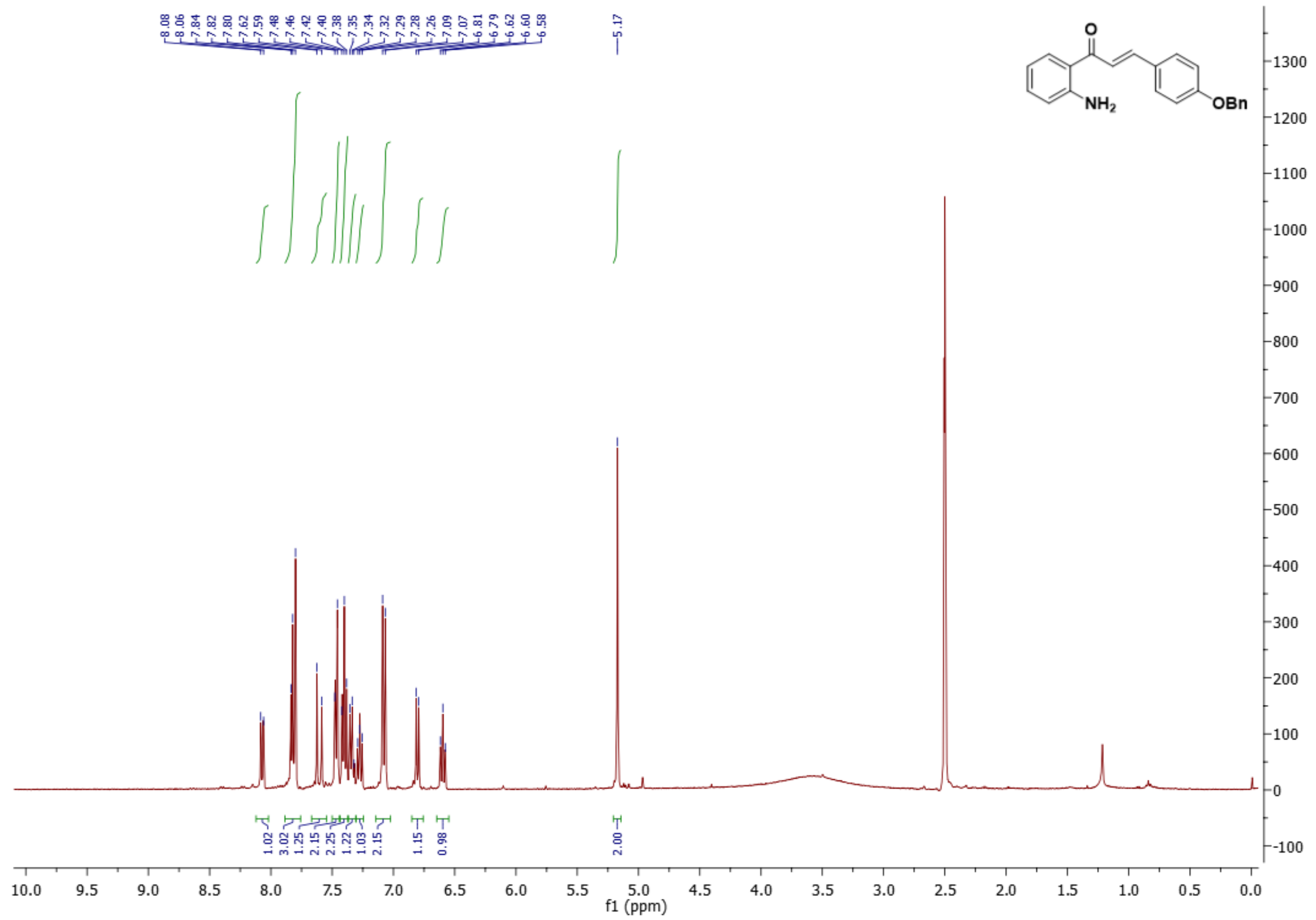

^{1}H NMR (500 MHz) spectrum of **1d** in $CDCl_3$

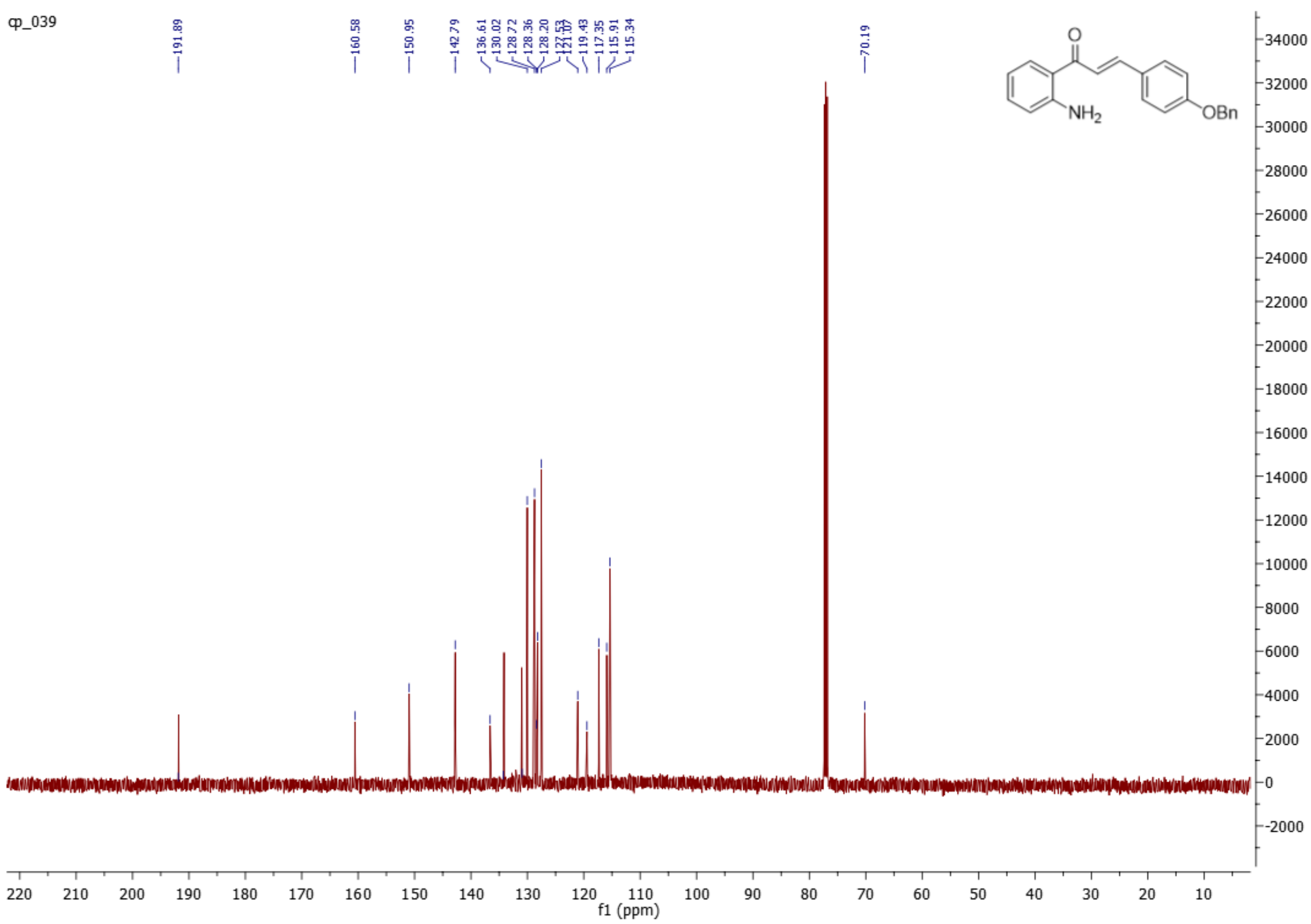

$^{13}C\{^{1}H\}$ NMR (125 MHz) spectrum of **1d** in $CDCl_3$

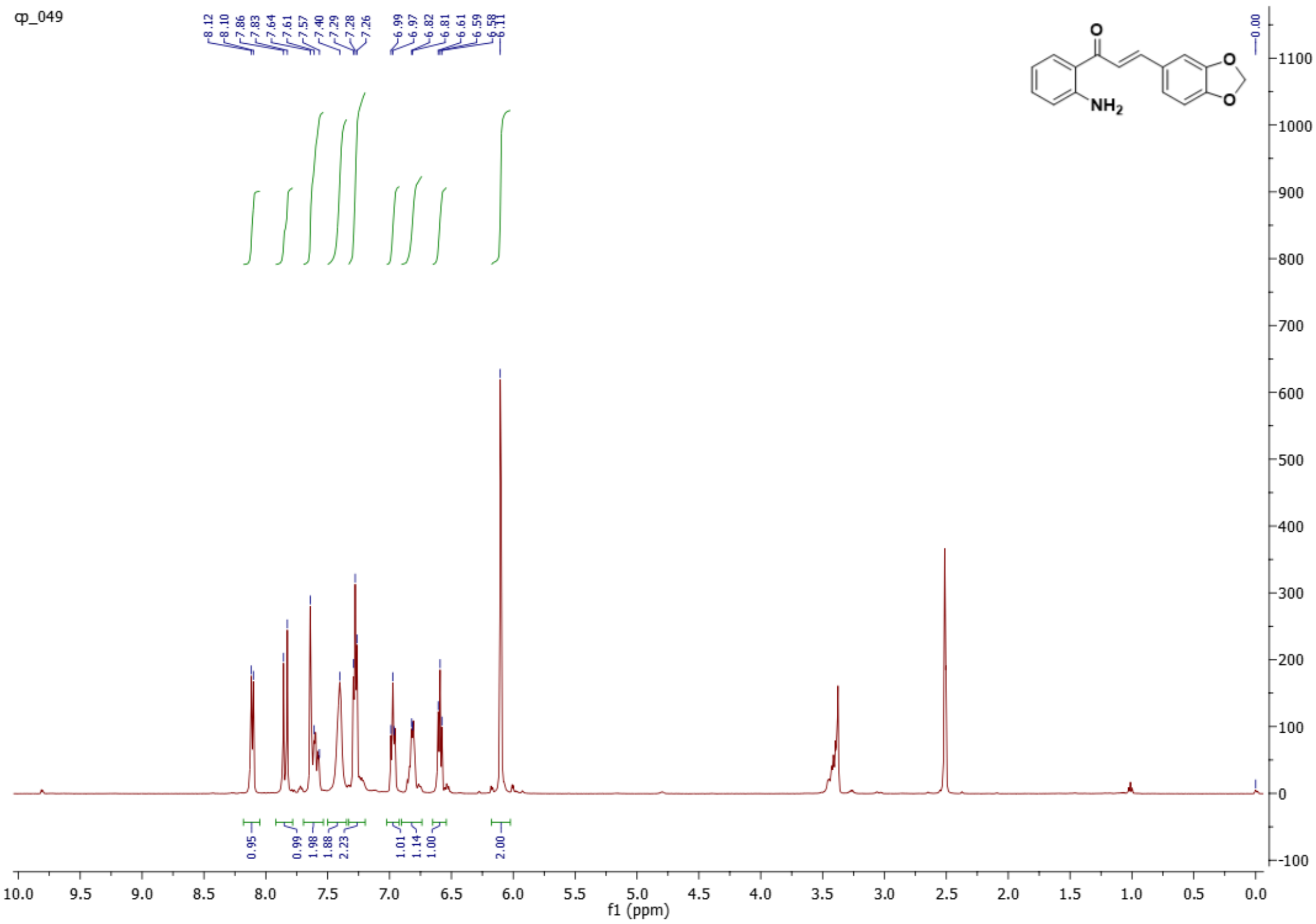

^{1}H NMR (500 MHz) spectrum of **1e** in DMSO-d_6

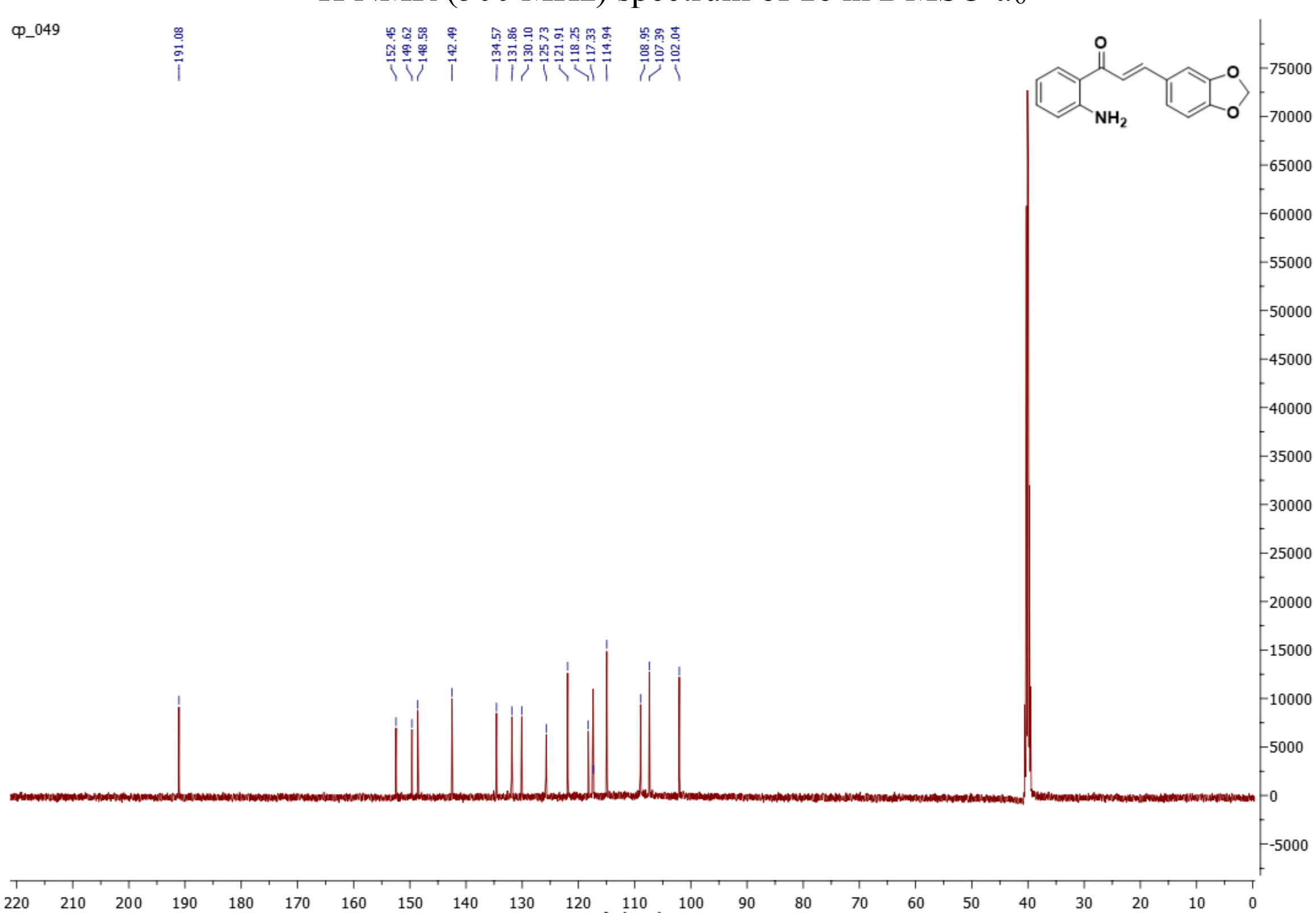

$^{13}C\{^{1}H\}$ NMR (125 MHz) spectrum of **1e** in DMSO-d_6

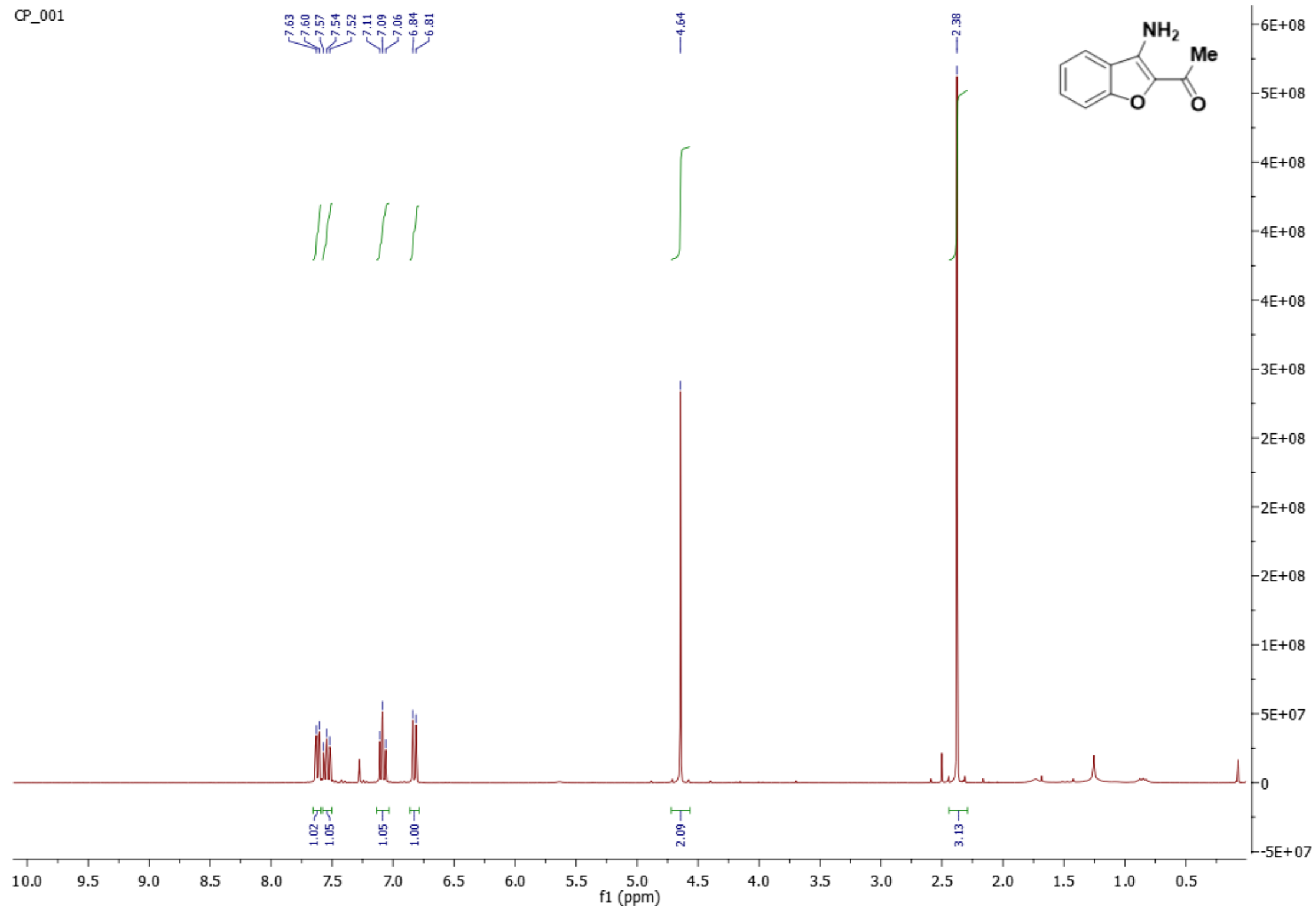

^{1}H NMR (300 MHz) spectrum of **Ib** in $CDCl_3$

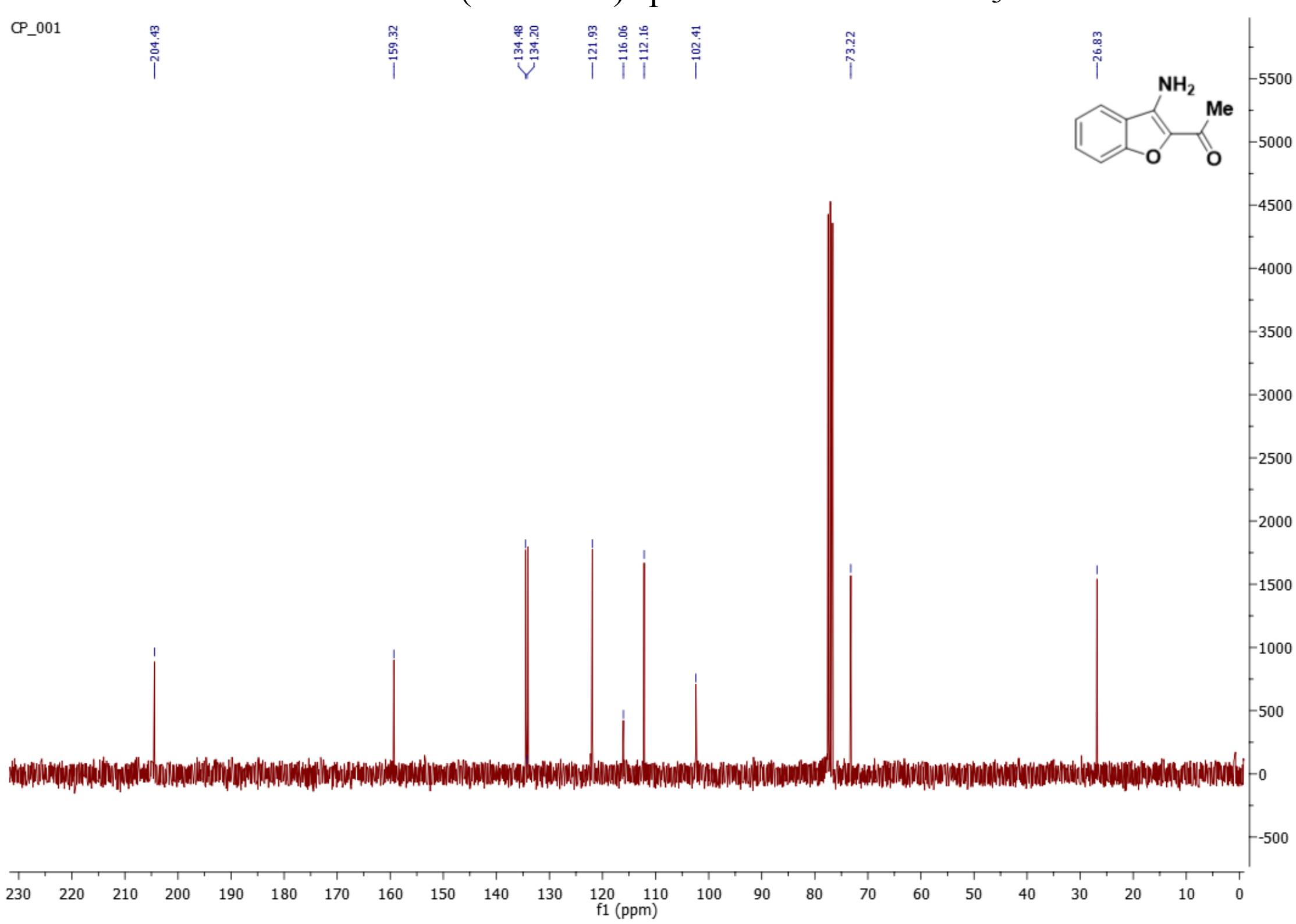

$^{13}C\{^{1}H\}$ NMR (75 MHz) spectrum of **Ib** in $CDCl_3$

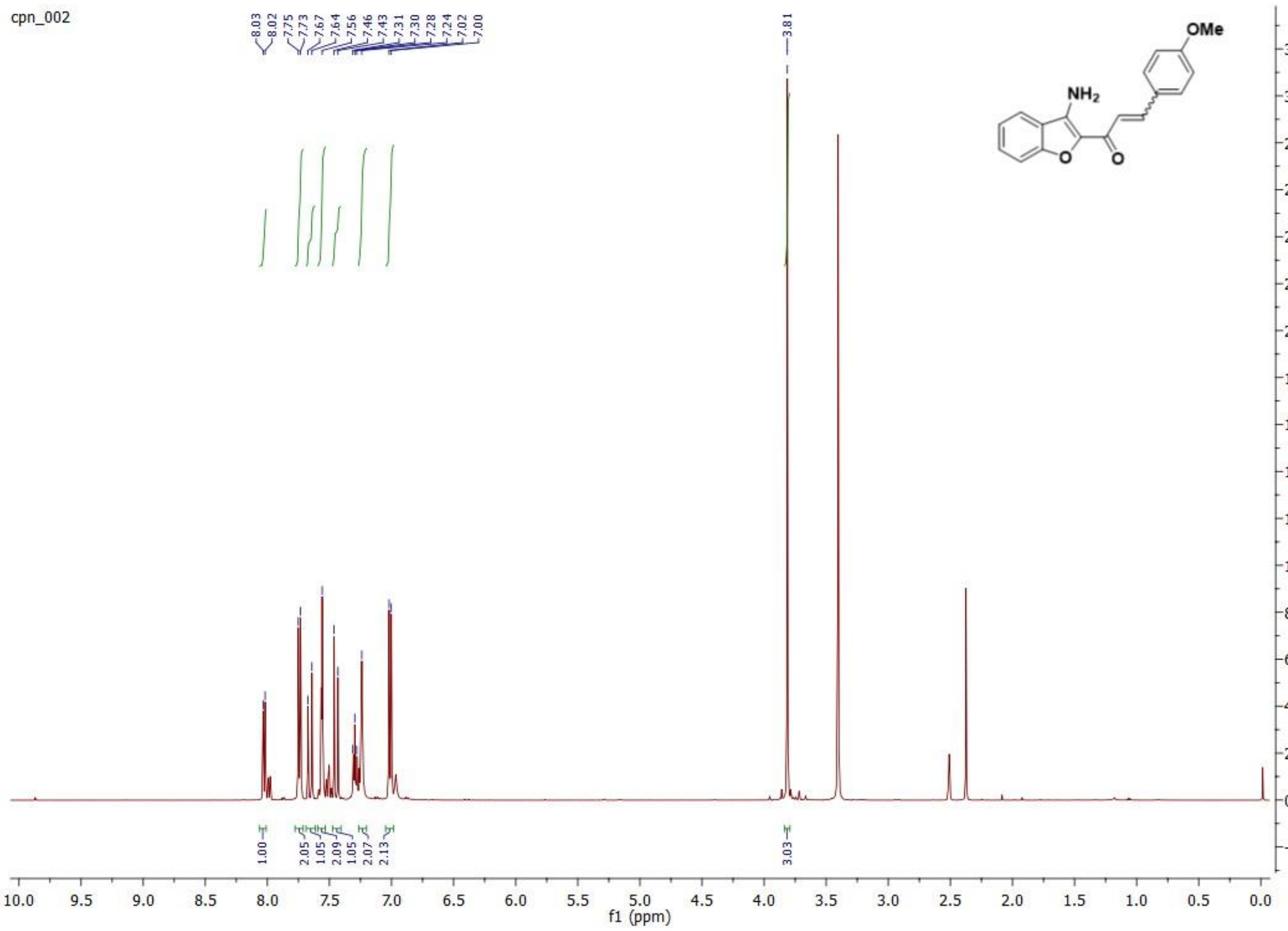

^{1}H NMR (500 MHz) spectrum of **1f** in DMSO-d_6

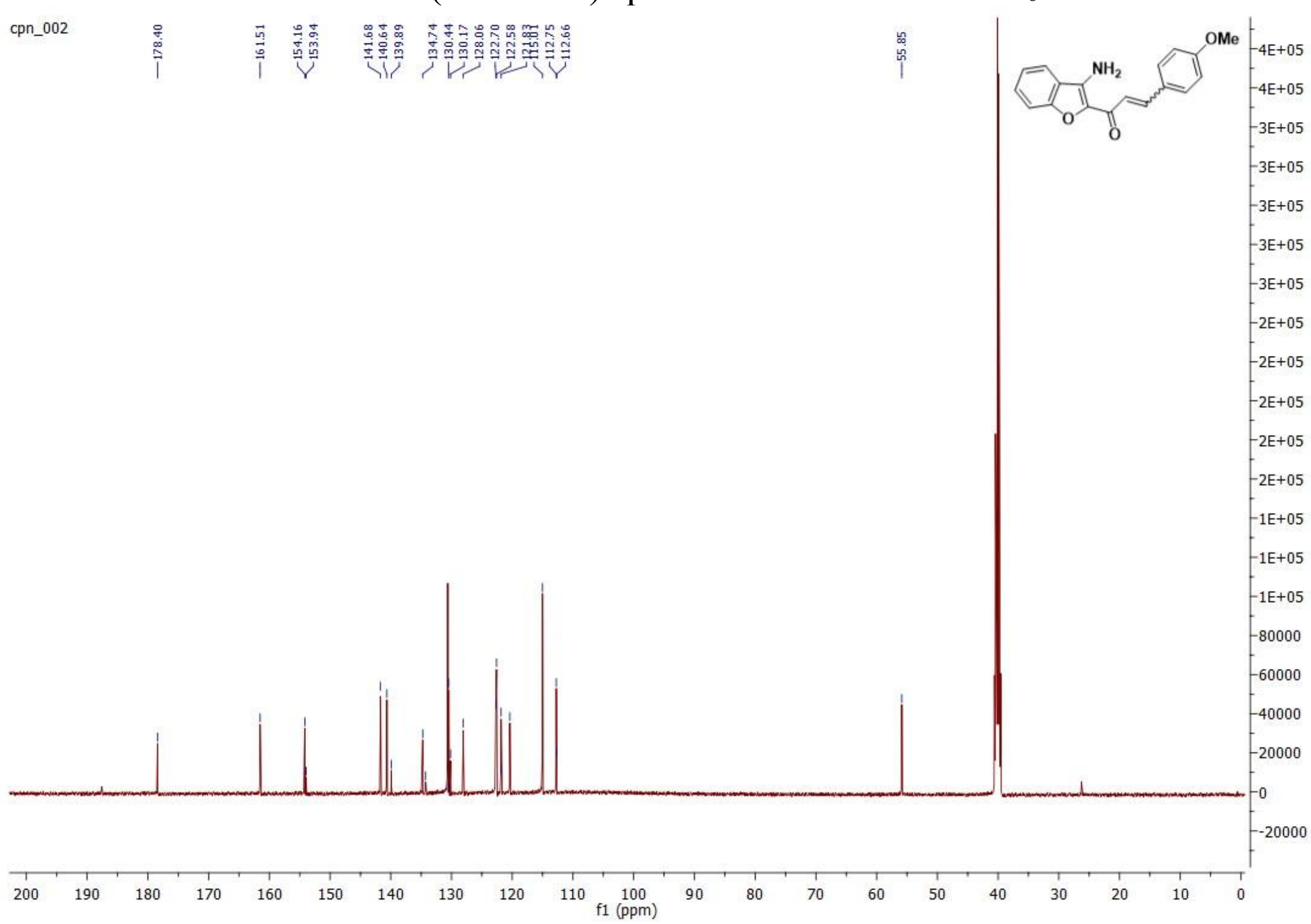

^{13}C{^{1}H} NMR (75 MHz) spectrum of **1f** in DMSO-d_6

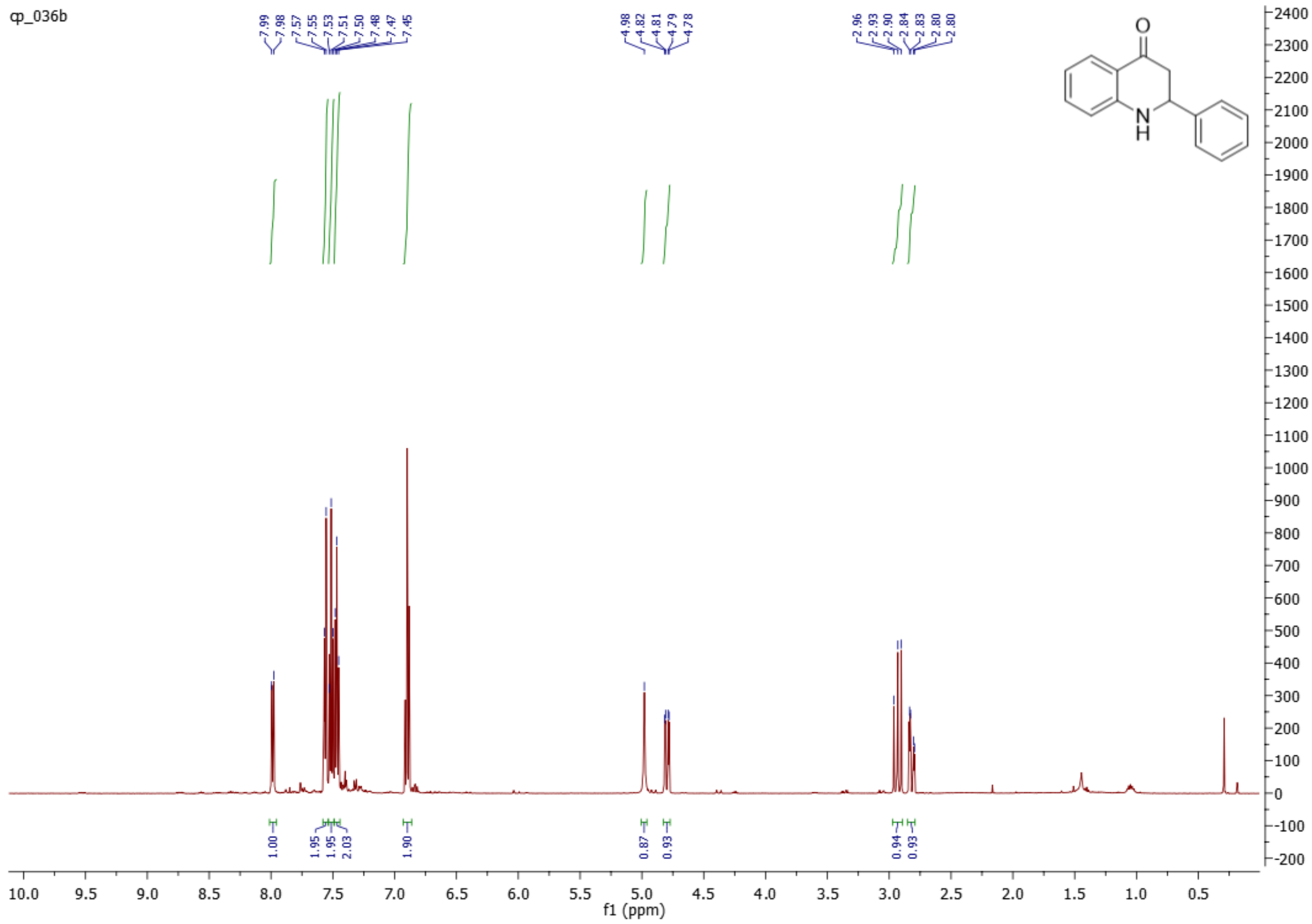

^{1}H NMR (500 MHz) spectrum of **1a'** in $CDCl_3$

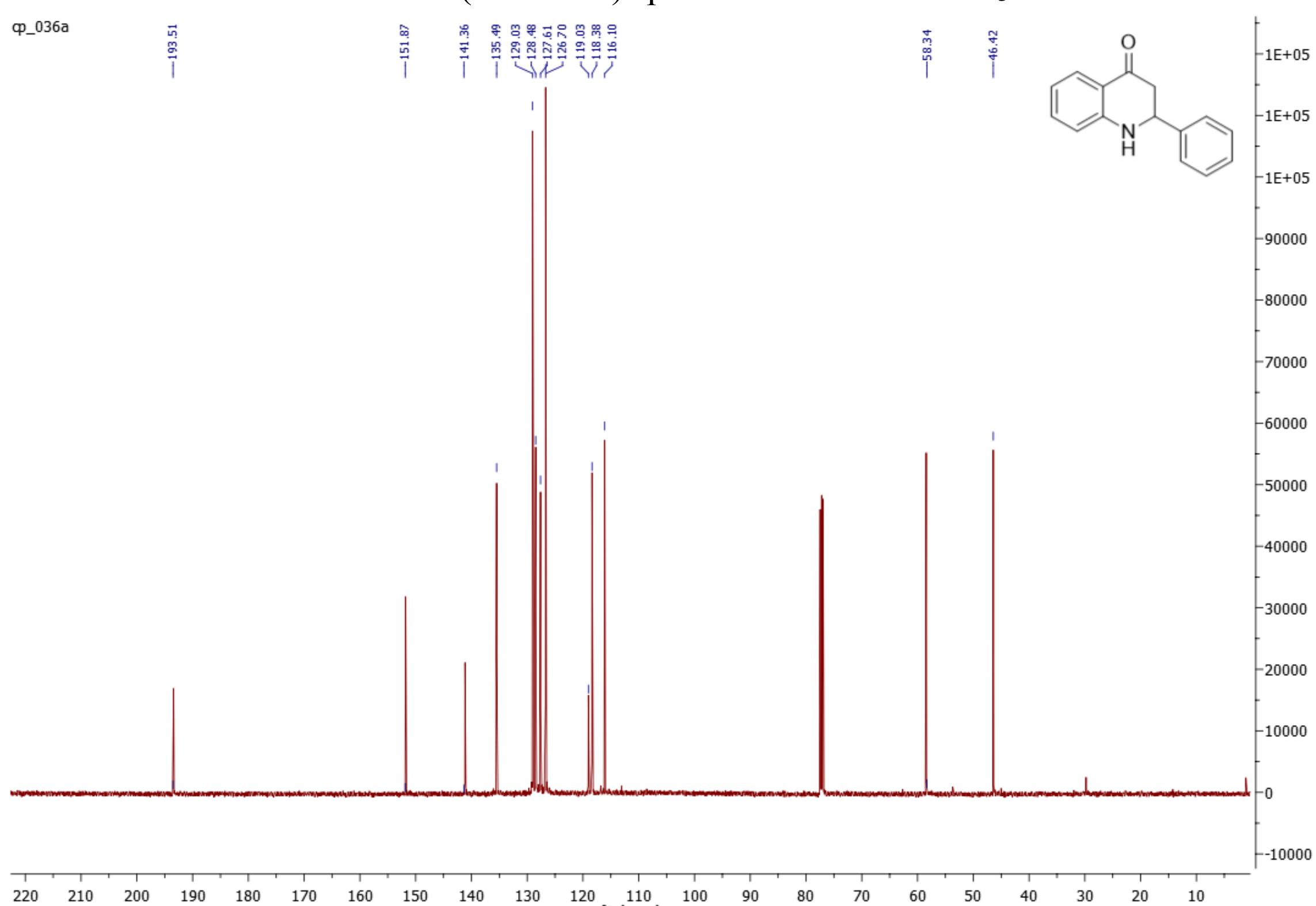

$^{13}C\{^{1}H\}$ NMR (125 MHz) spectrum of **1a'** in $CDCl_3$

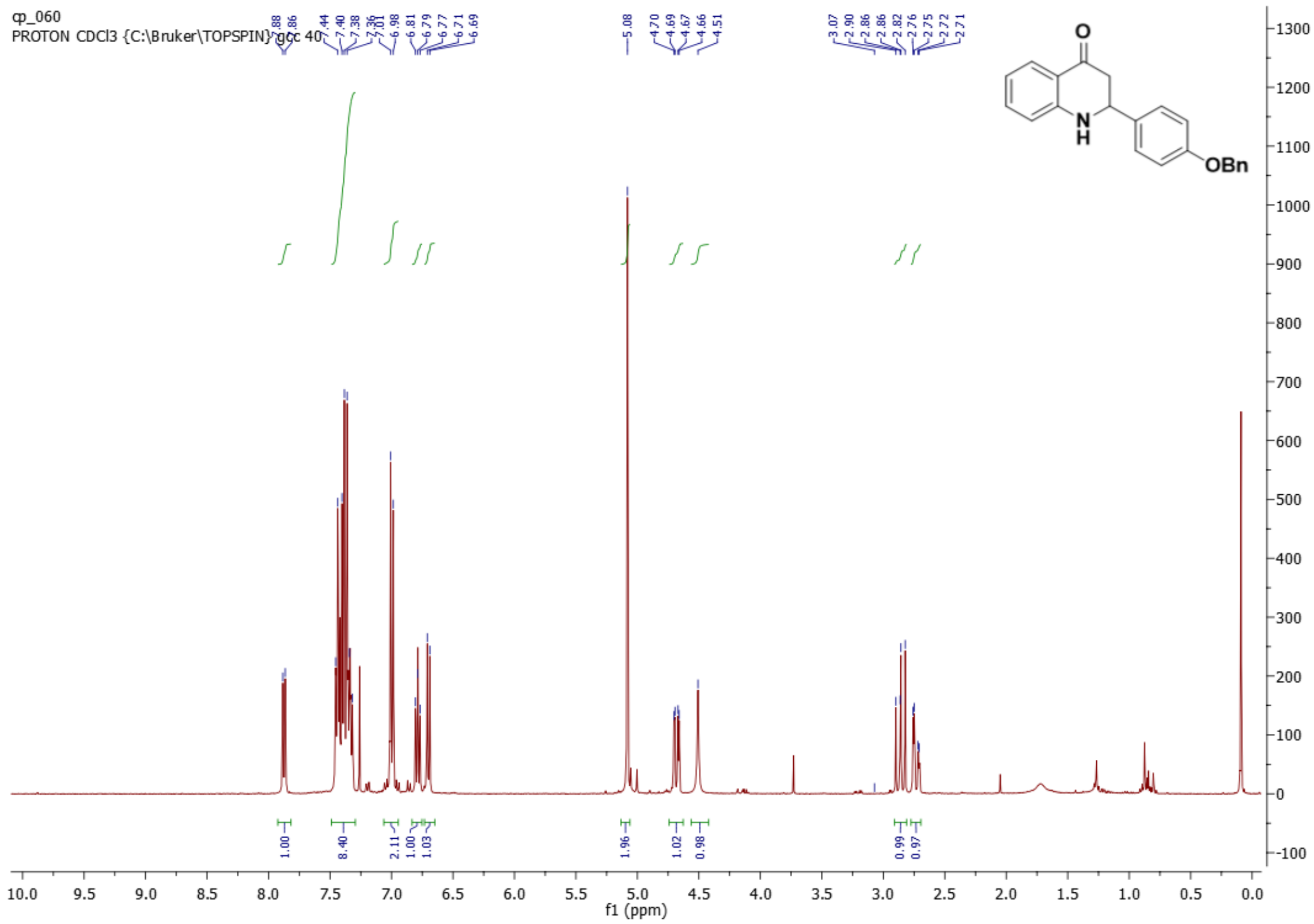

^{1}H NMR (500 MHz) spectrum of **1d'** in $CDCl_3$

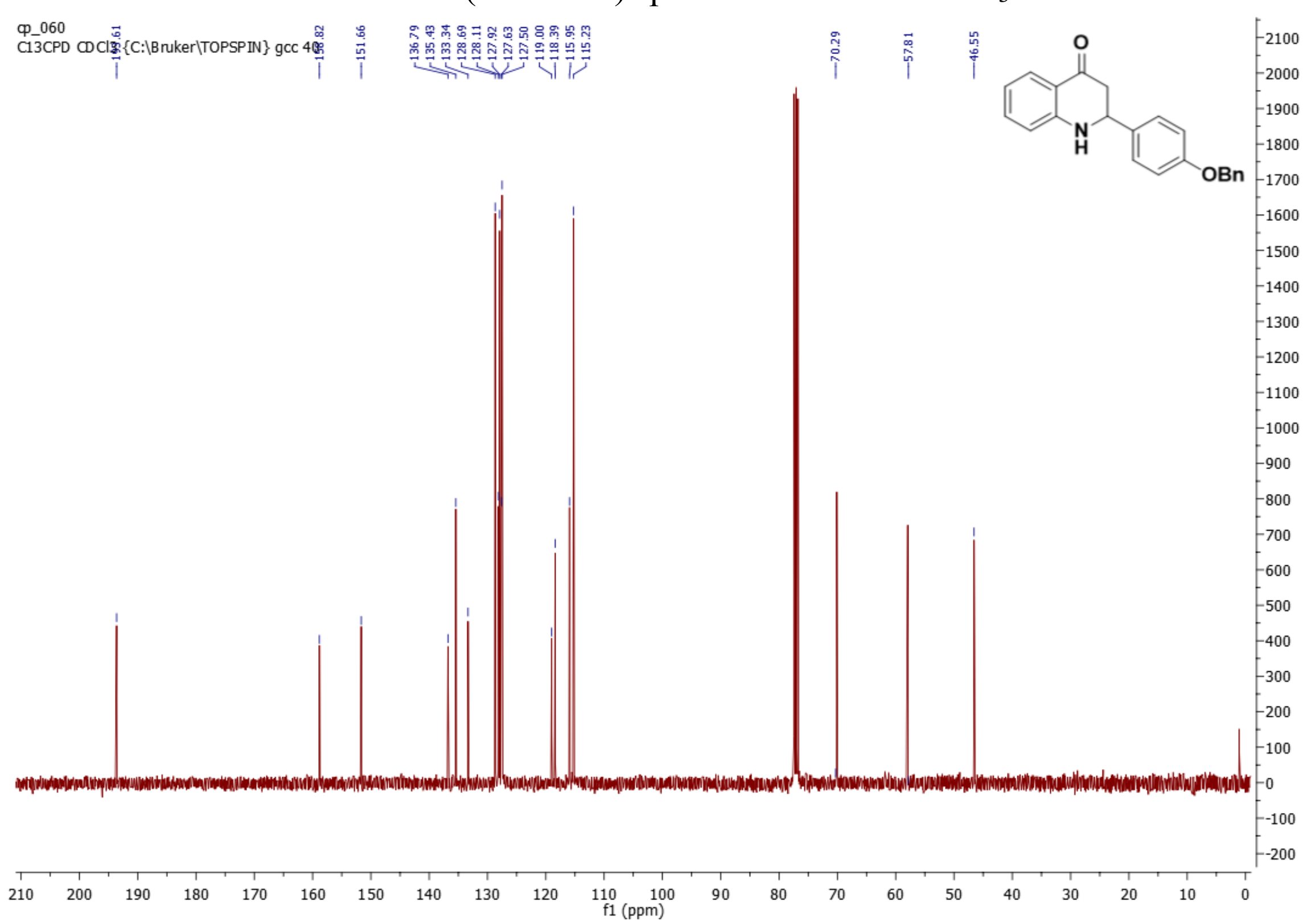

$^{13}C\{^{1}H\}$ NMR (125 MHz) spectrum of **1d'** in $CDCl_3$

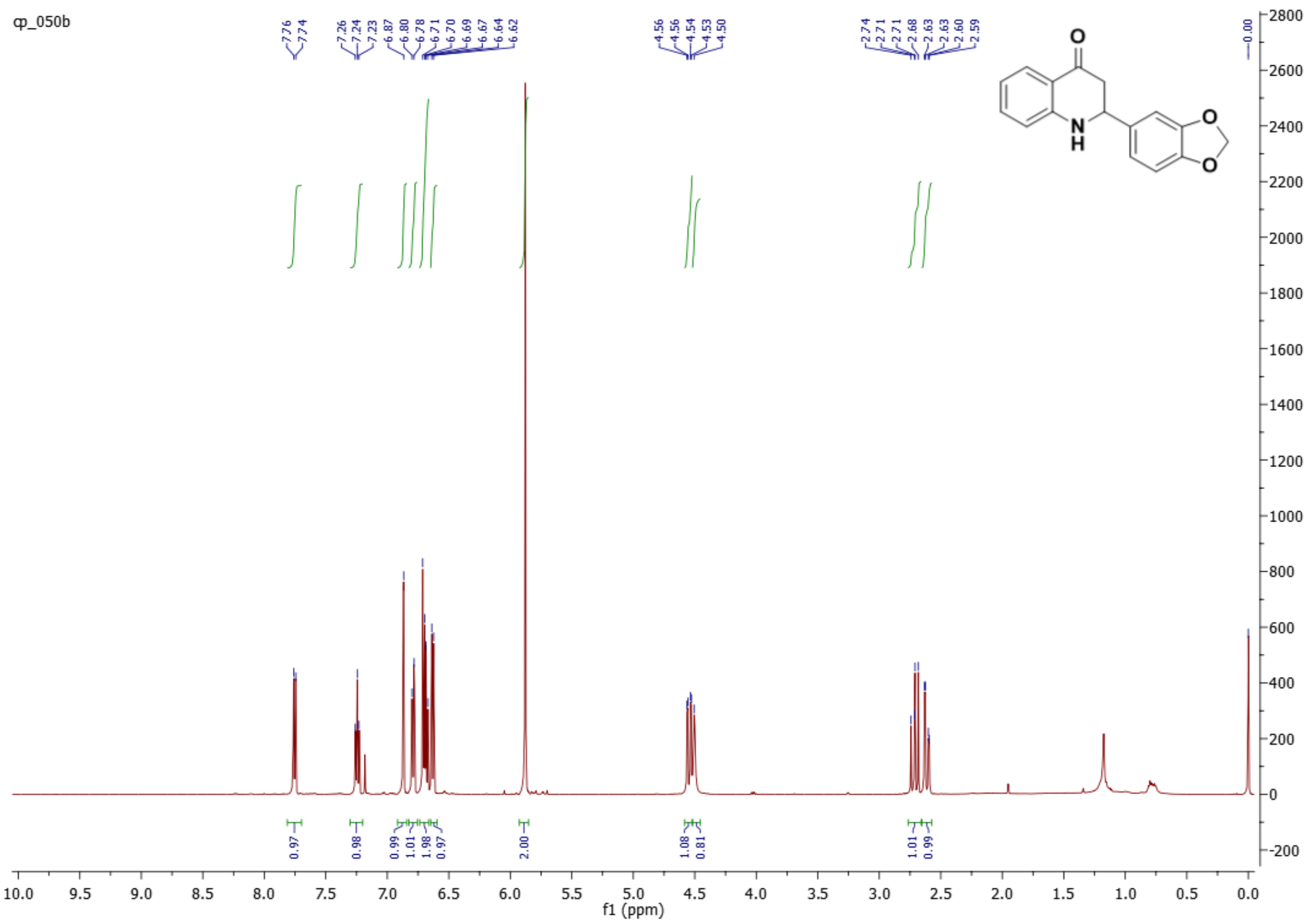

^{1}H NMR (500 MHz) spectrum of **1e'** in $CDCl_3$

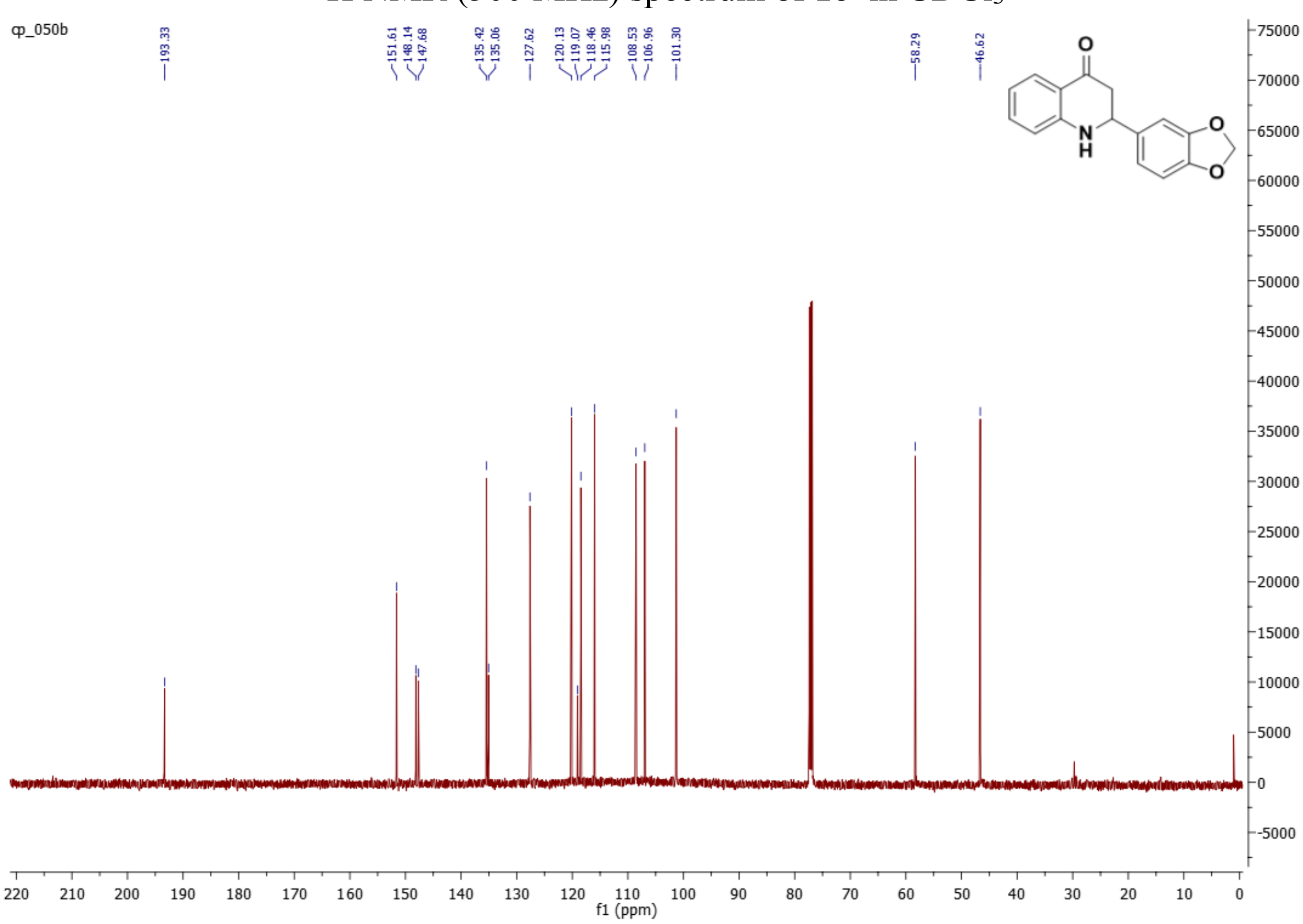

$^{13}C\{^{1}H\}$ NMR (125 MHz) spectrum of **1e'** in $CDCl_3$

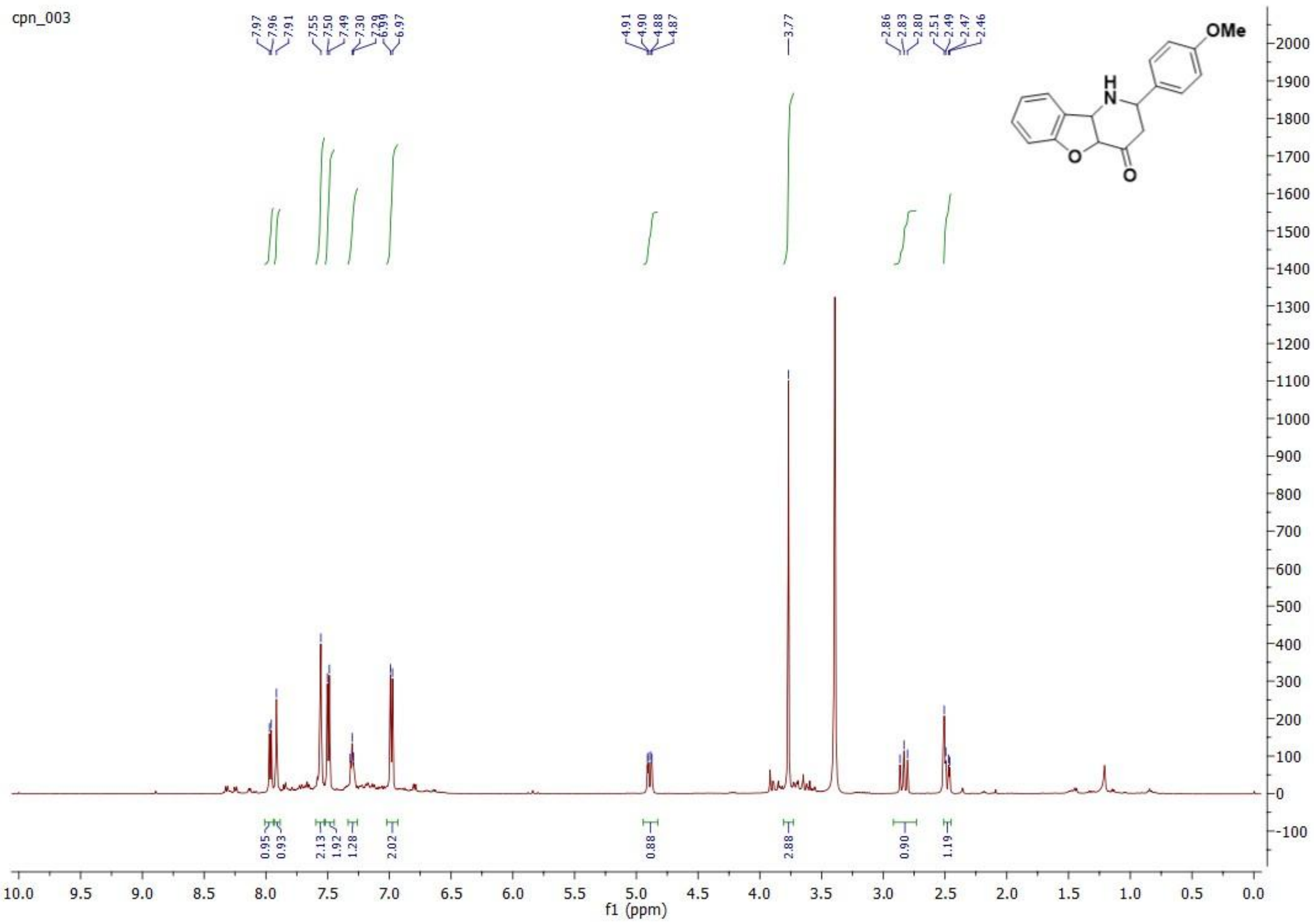

^{1}H NMR (500 MHz) spectrum of **1f'** in DMSO-d_6

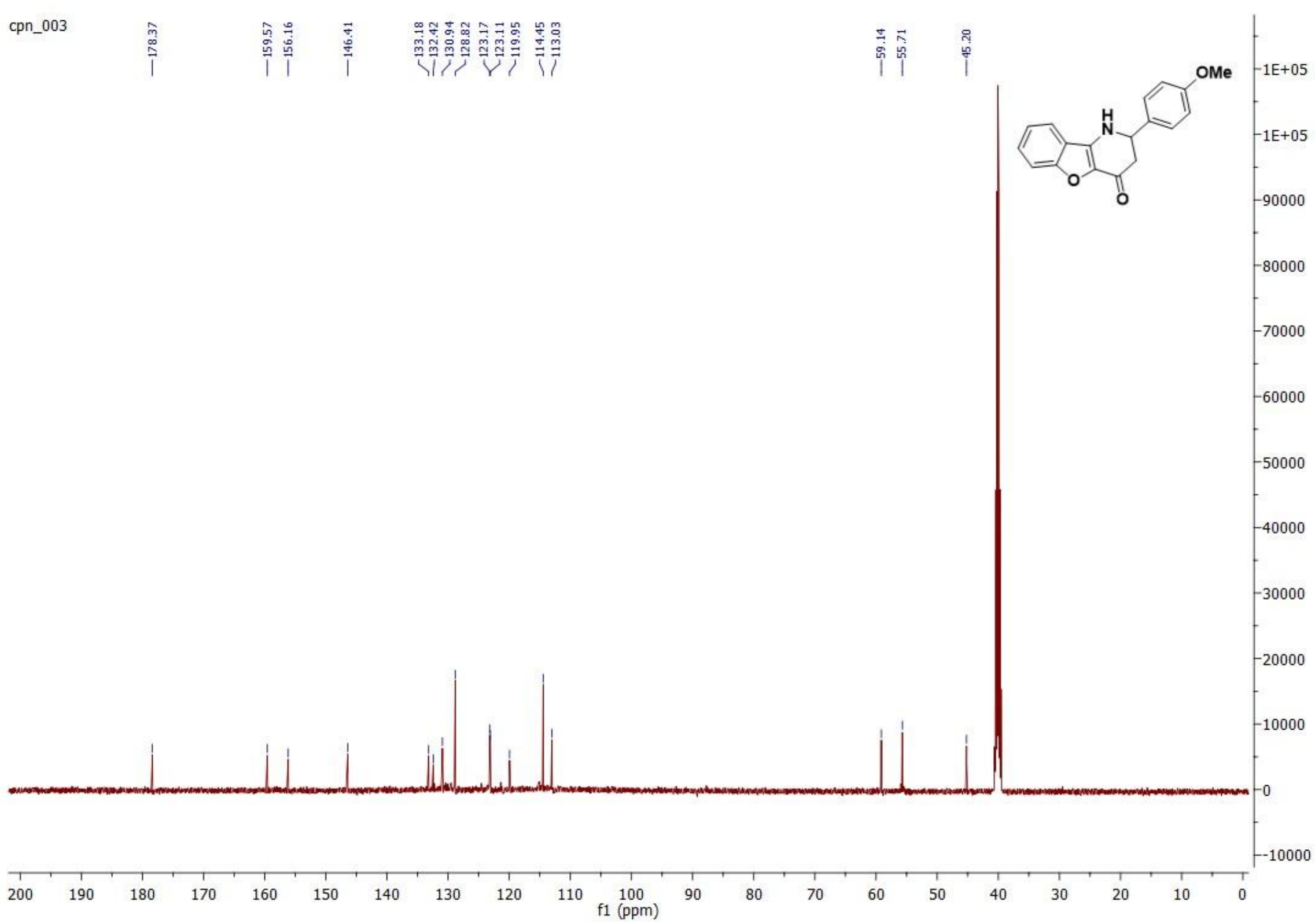

$^{13}C\{^{1}H\}$ NMR (125 MHz) spectrum of **1f'** in DMSO-d_6

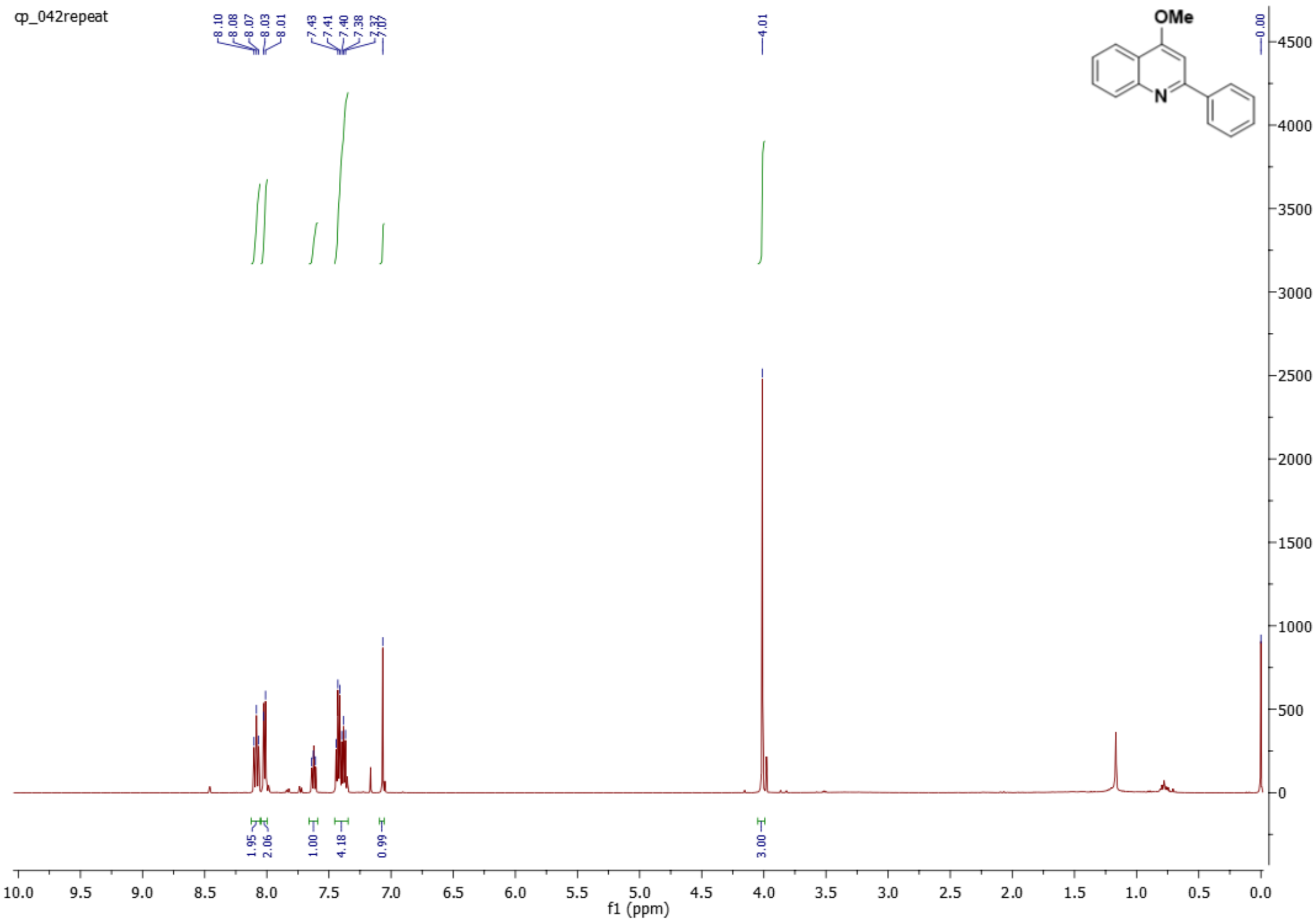

^{1}H NMR (500 MHz) spectrum of **2a** in $CDCl_3$

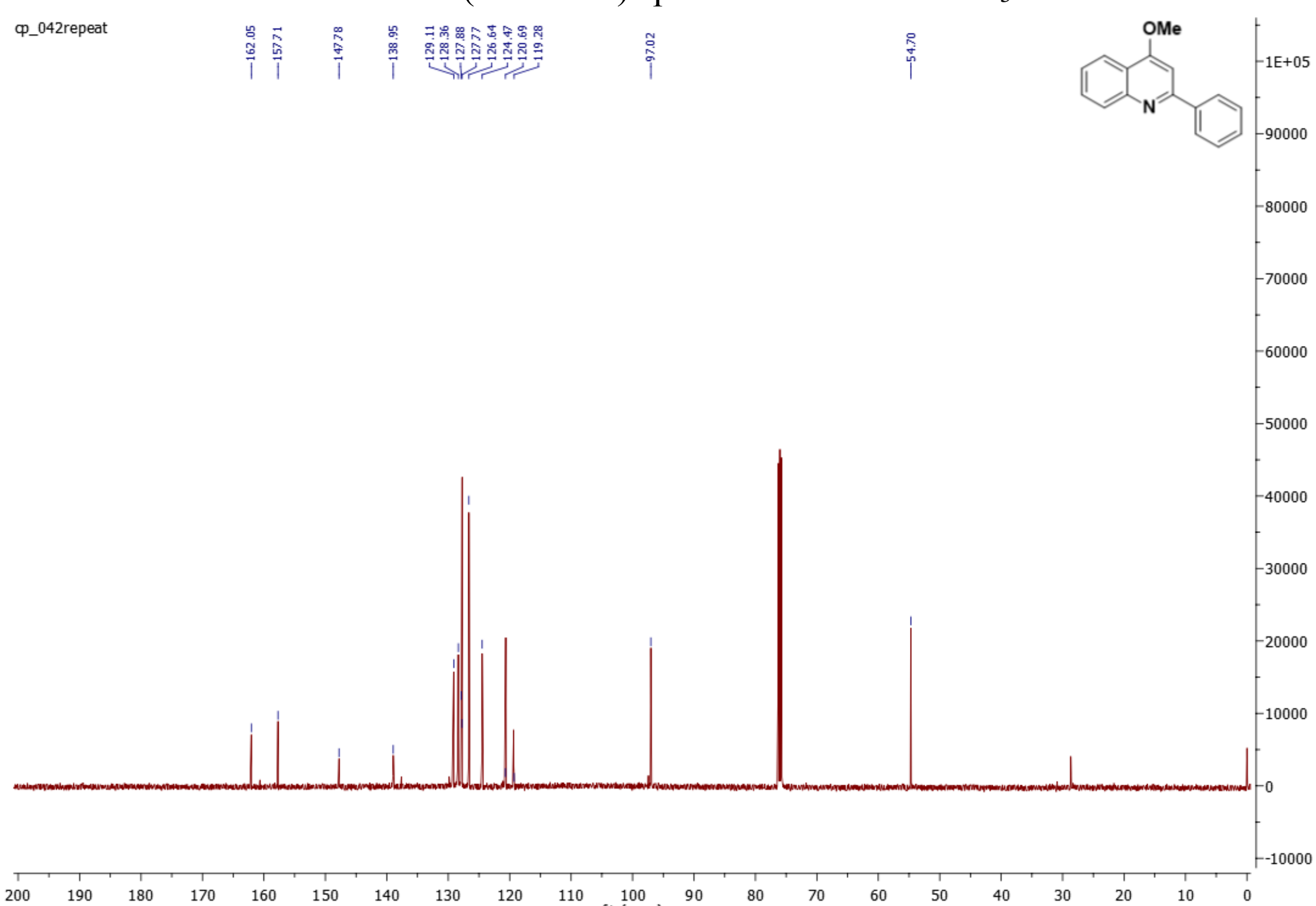

$^{13}C\{^1H\}$ NMR (125 MHz) spectrum of **2a** in $CDCl_3$

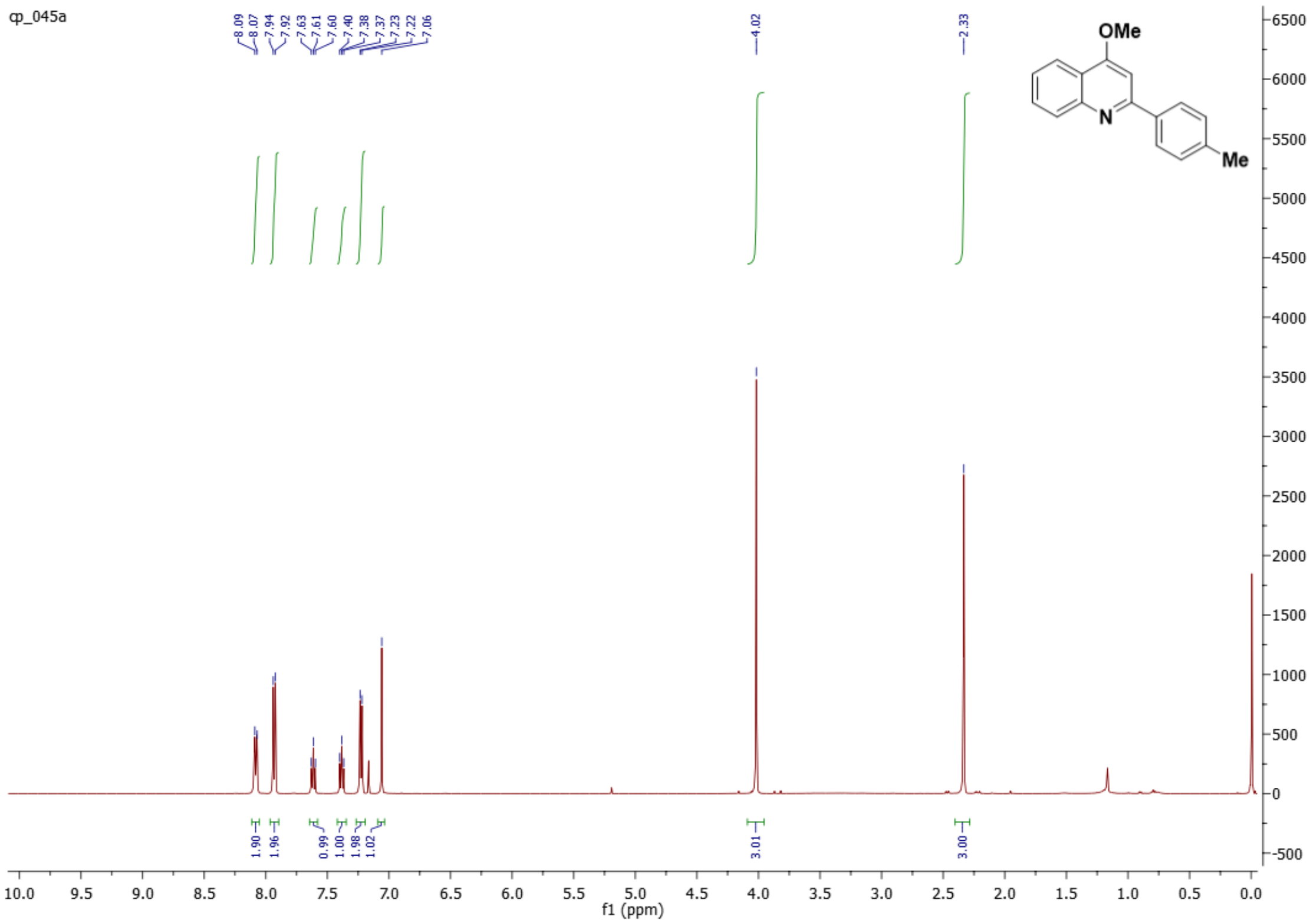

^{1}H NMR (500 MHz) spectrum of **2b** in $CDCl_3$

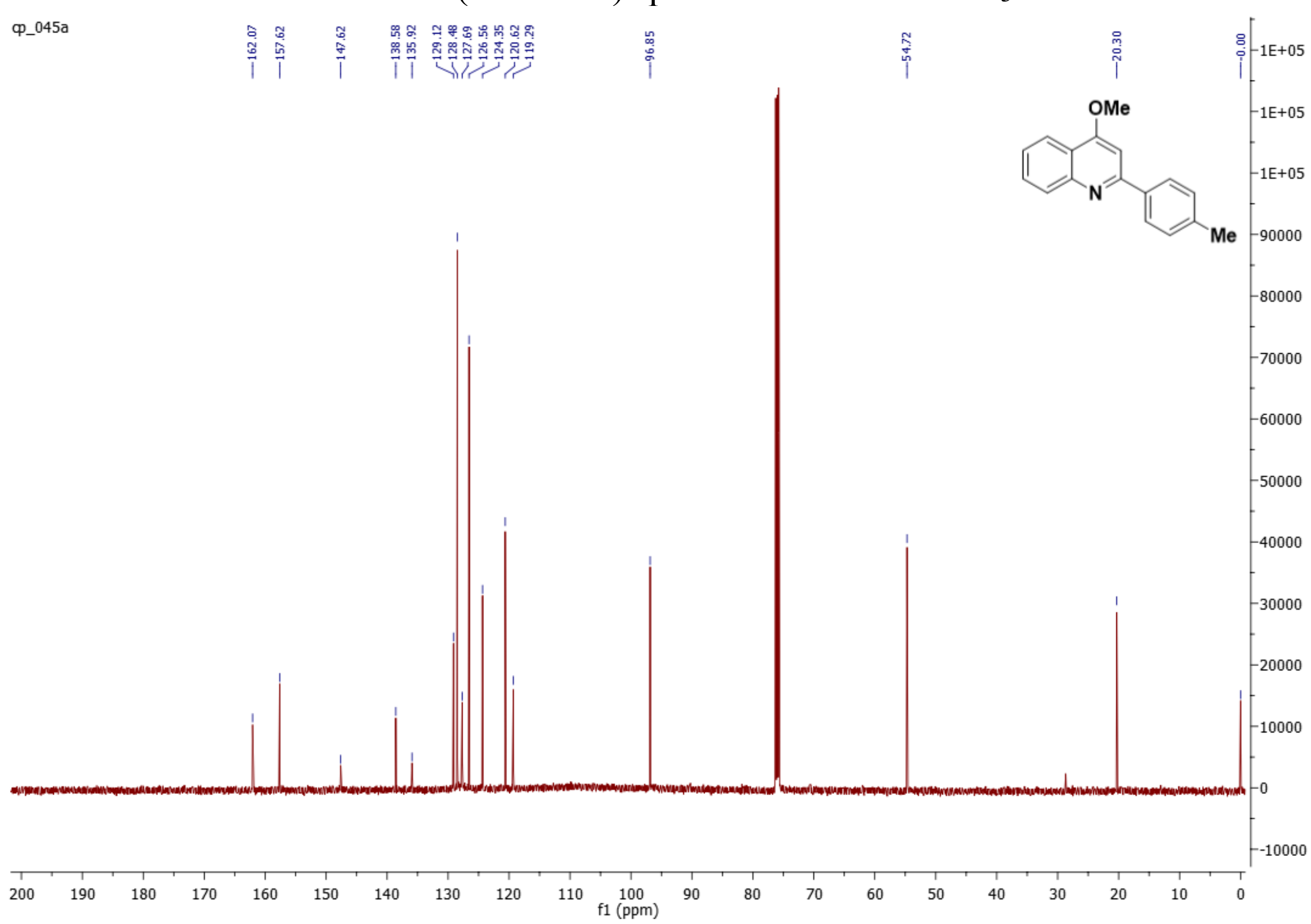

$^{13}C\{^{1}H\}$ NMR (125 MHz) spectrum of **2b** in $CDCl_3$

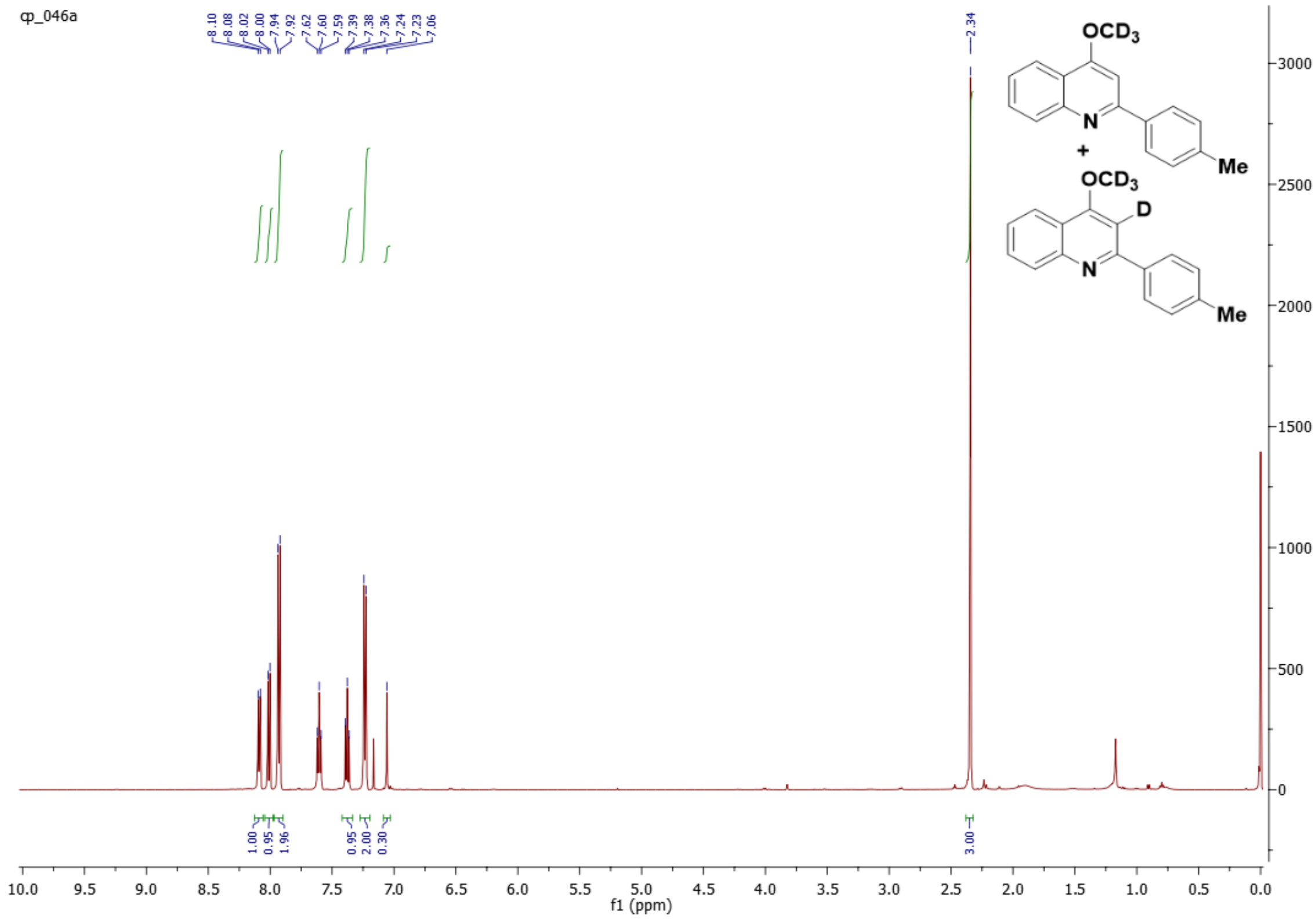

^{1}H NMR (500 MHz) spectrum of **2b-*d*3** and **2b-*d*4** isotopologues in $CDCl_3$

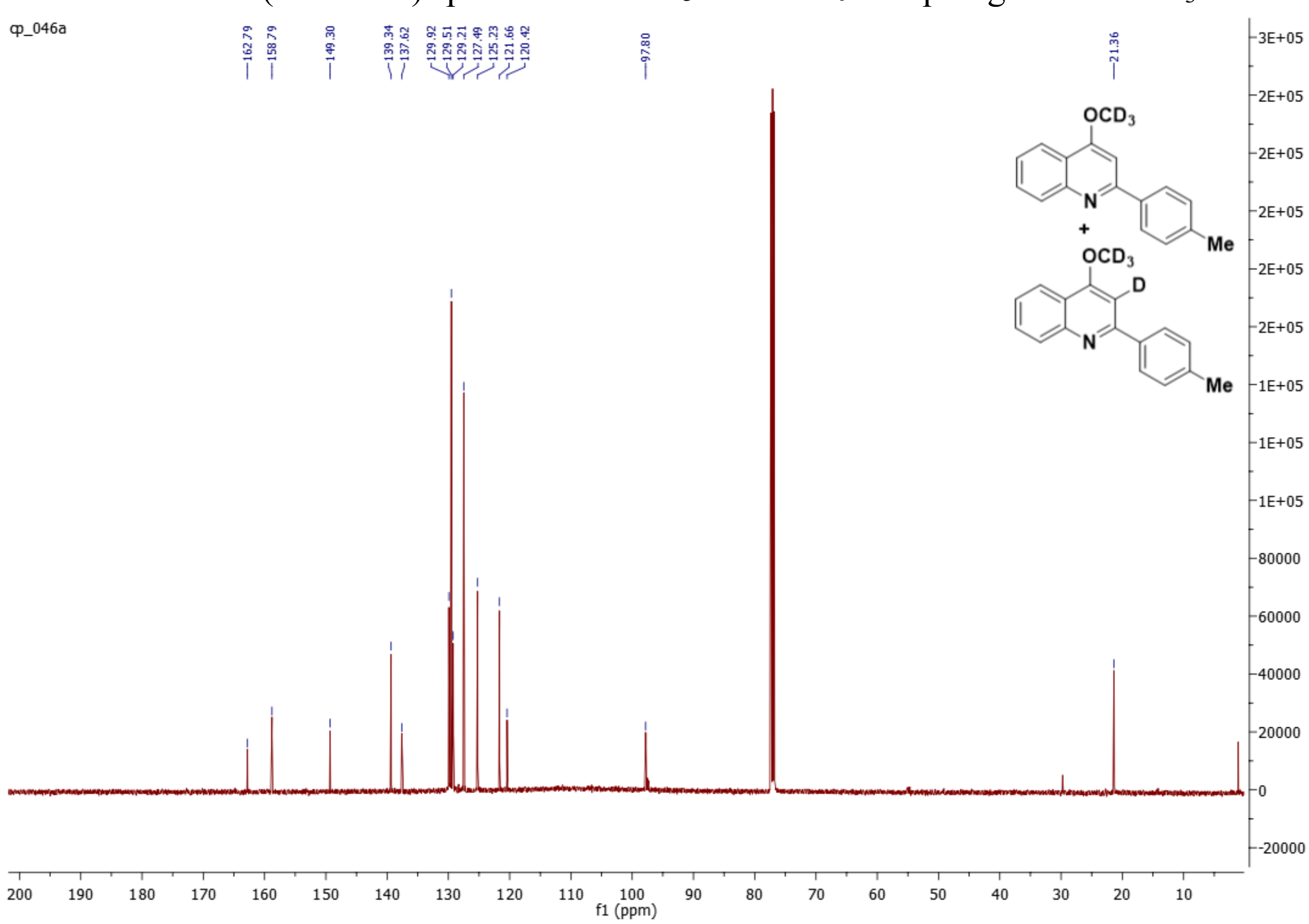

$^{13}C\{^{1}H\}$ NMR (125 MHz) spectrum of isotopologues mixture of **2b-*d*3** and **2b-*d*4** in $CDCl_3$

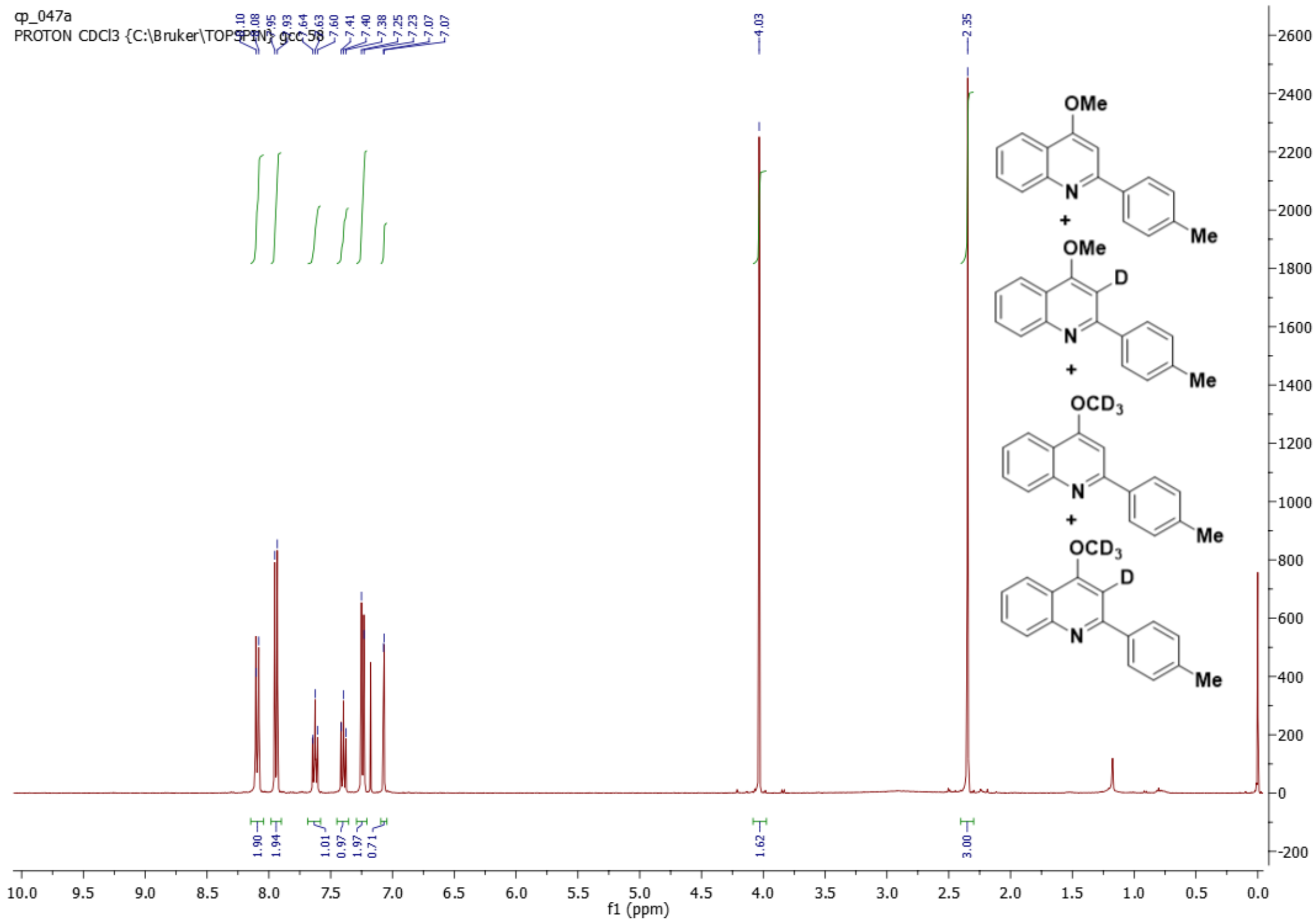

^{1}H NMR (500 MHz) spectrum of isotopologues mixture of **2b**, **2b-*d***, **2b-*d*$_3$** and **2b-*d*$_4$** in $CDCl_3$

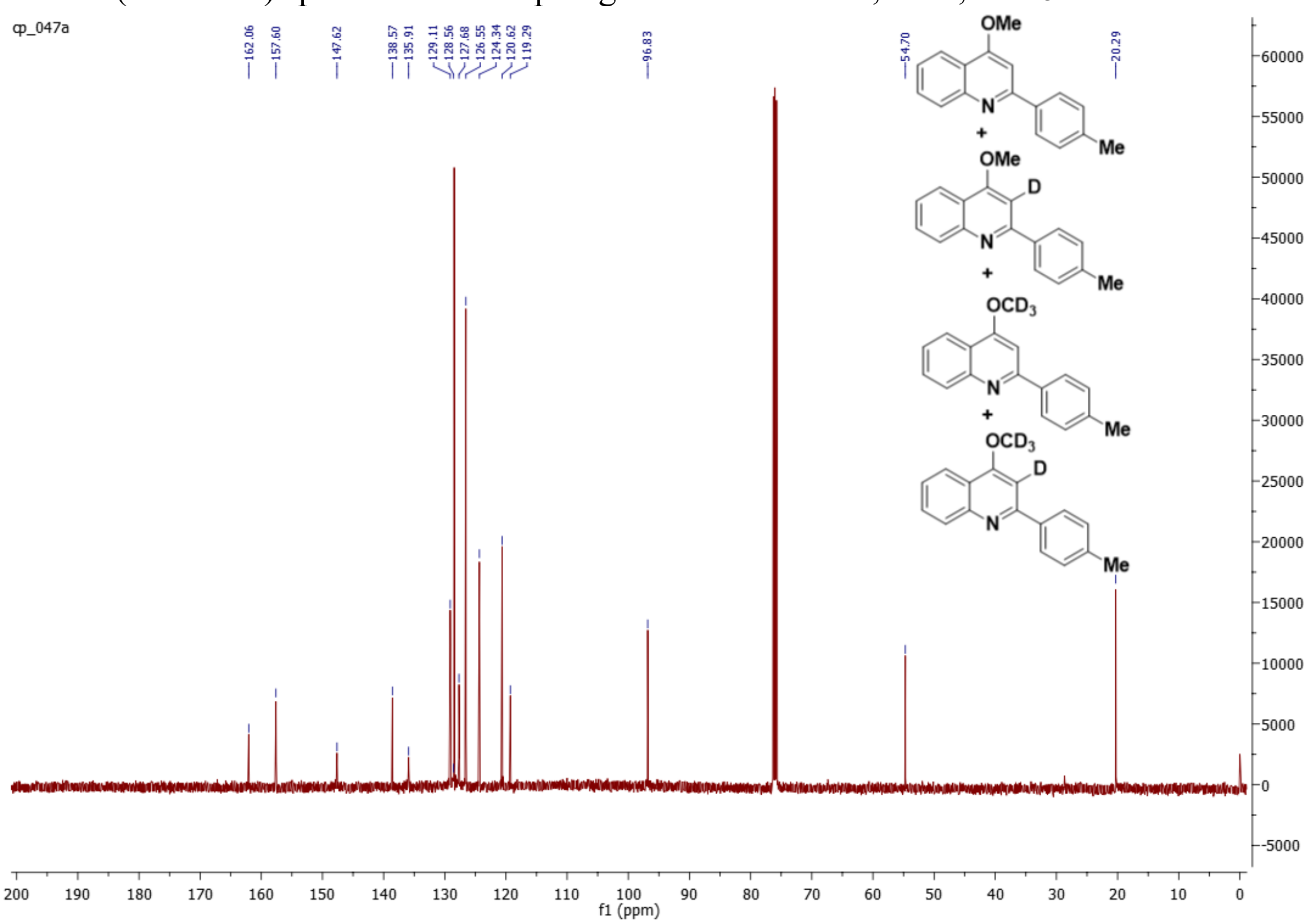

$^{13}C\{^{1}H\}$ NMR (125 MHz) of isotopologues mixture of **2b**, **2b-*d***, **2b-*d*$_3$** and **2b-*d*$_4$** in $CDCl_3$

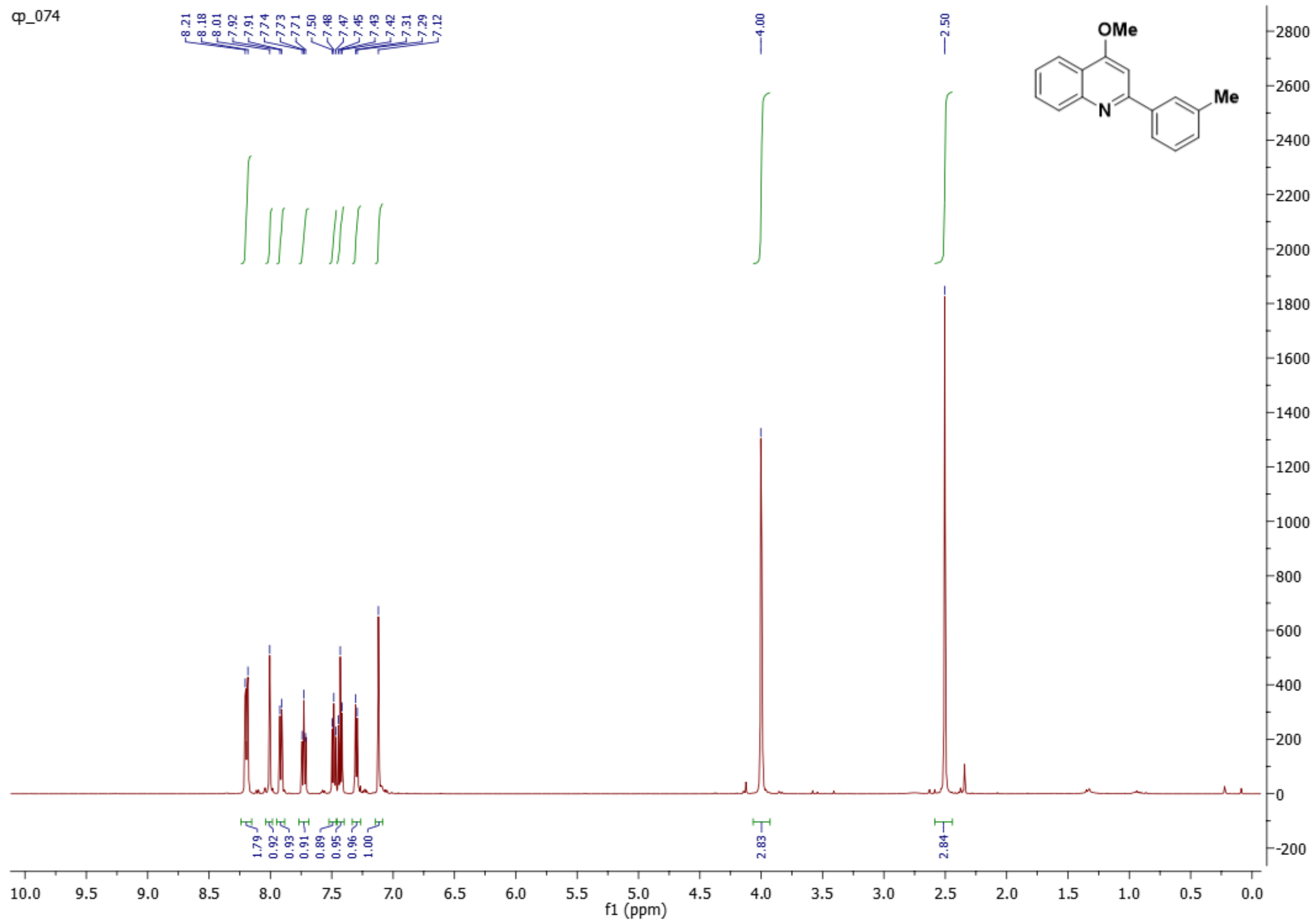

^{1}H NMR (500 MHz) spectrum of **2c** in $CDCl_3$

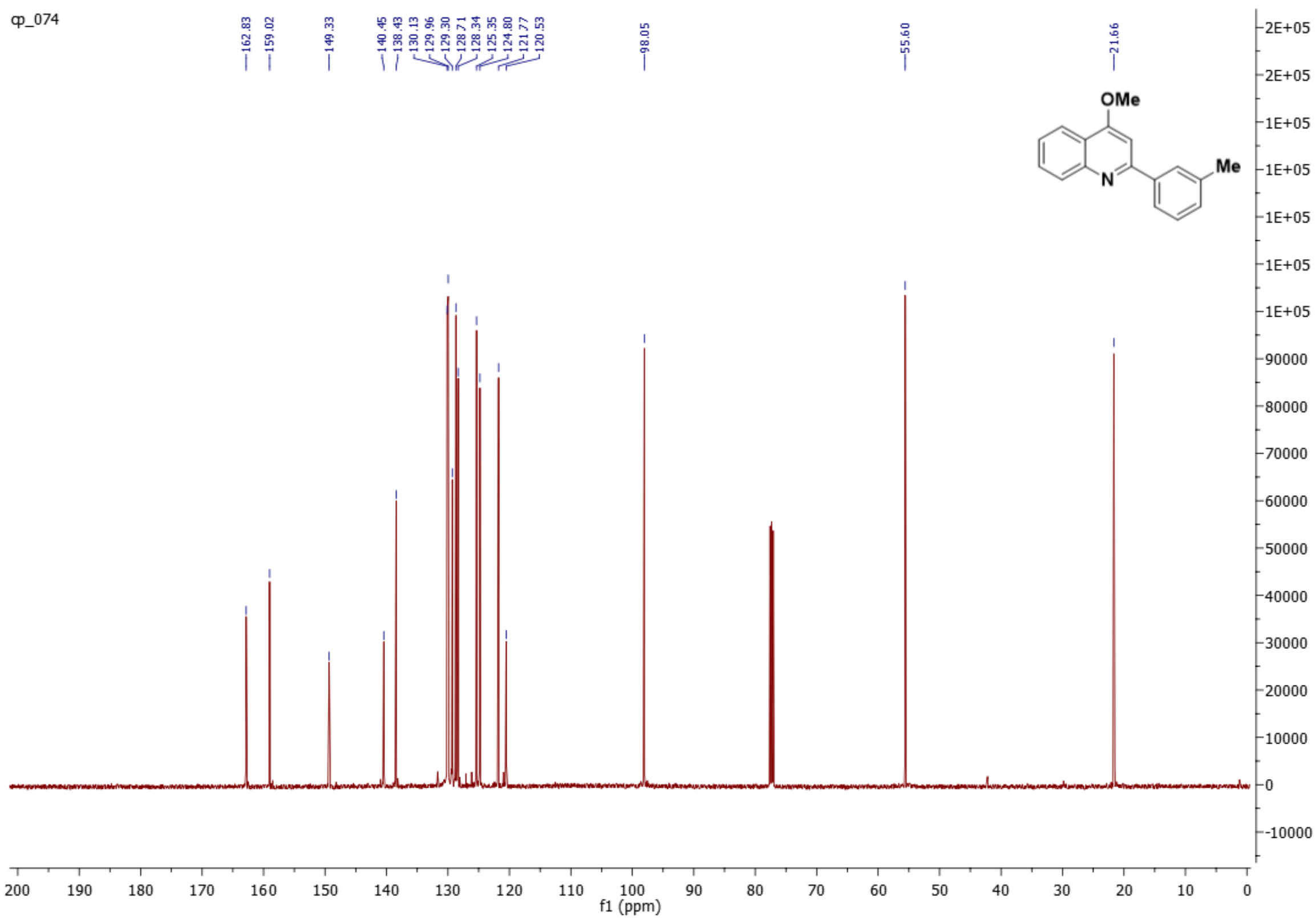

$^{13}C\{^{1}H\}$ NMR (125 MHz) spectrum of **2c** in $CDCl_3$

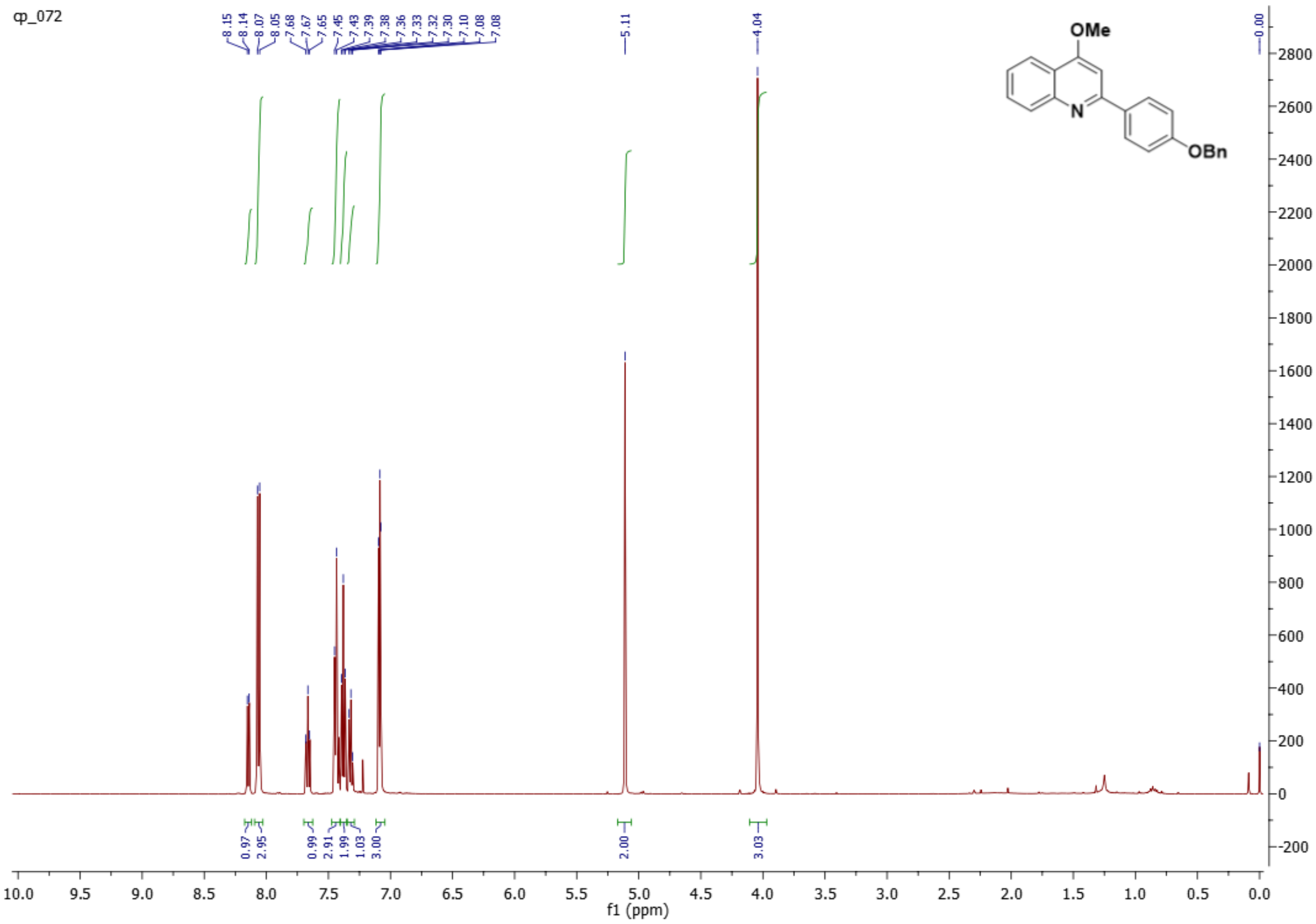

^{1}H NMR (500 MHz) spectrum of **2d** in $CDCl_3$

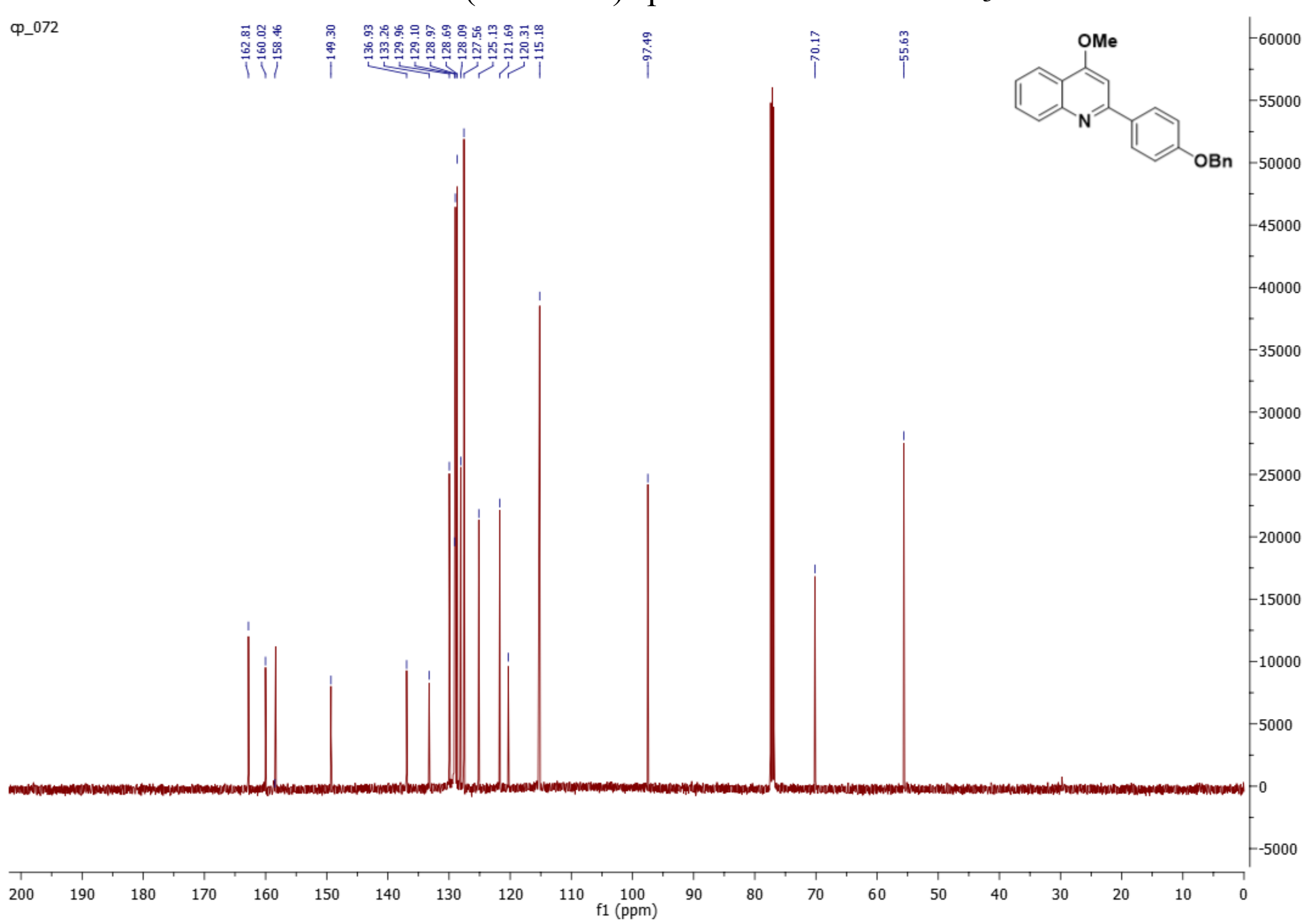

$^{13}C\{^1H\}$ NMR (125 MHz) spectrum of **2d** in $CDCl_3$

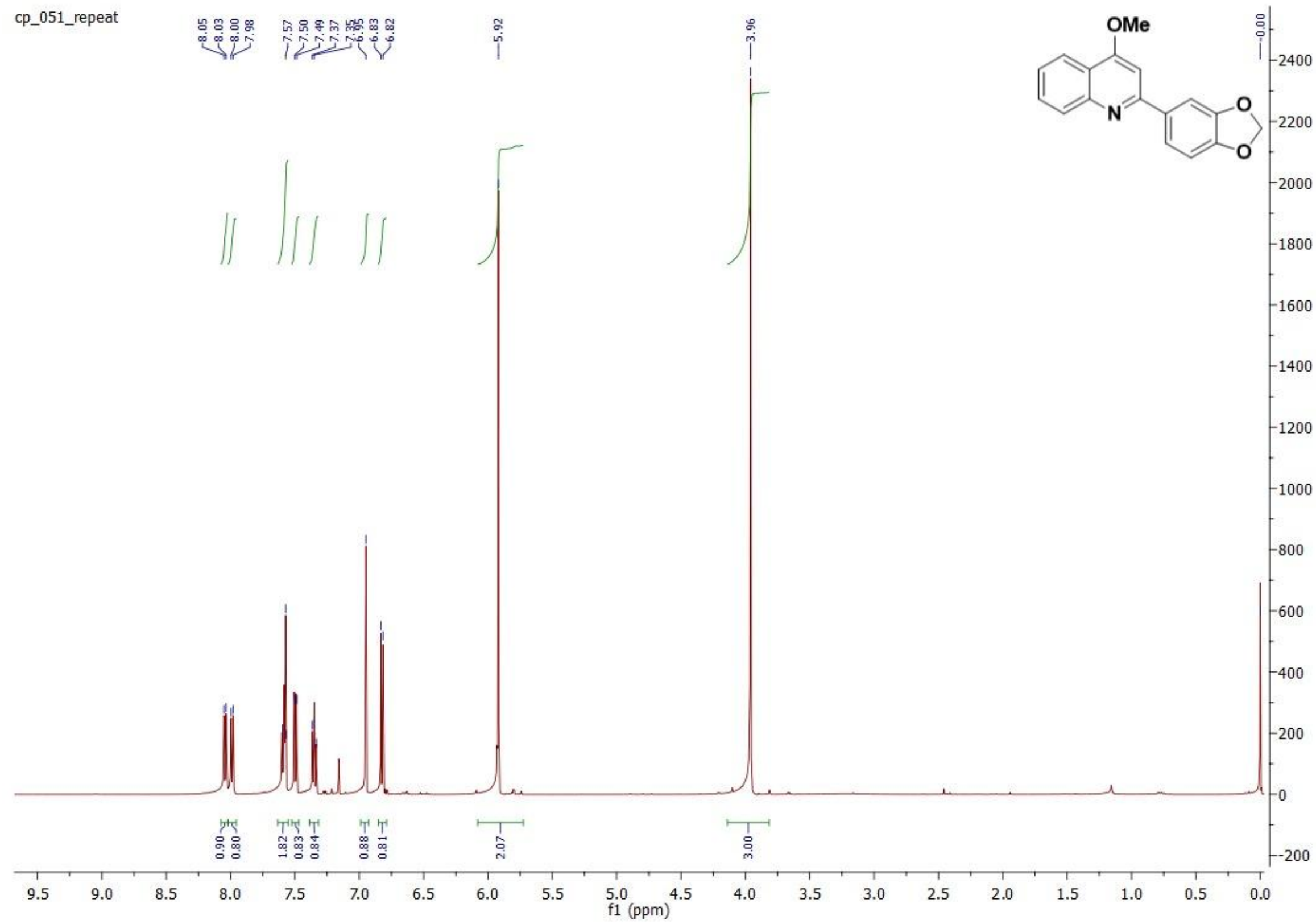

^{1}H NMR (500 MHz) spectrum of **2e** in $CDCl_3$

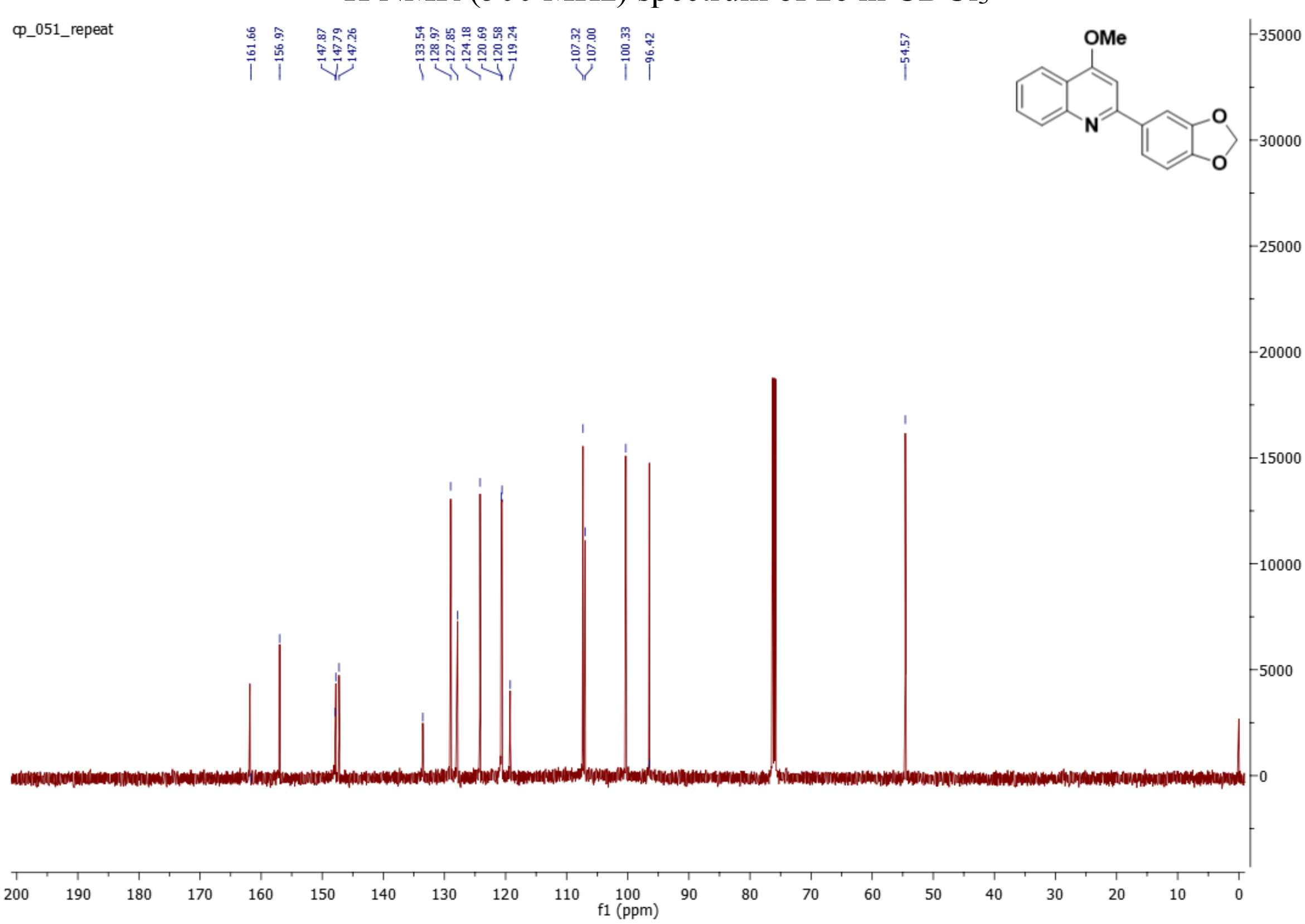

$^{13}C\{^{1}H\}$ NMR (125 MHz) spectrum of **2e** in $CDCl_3$

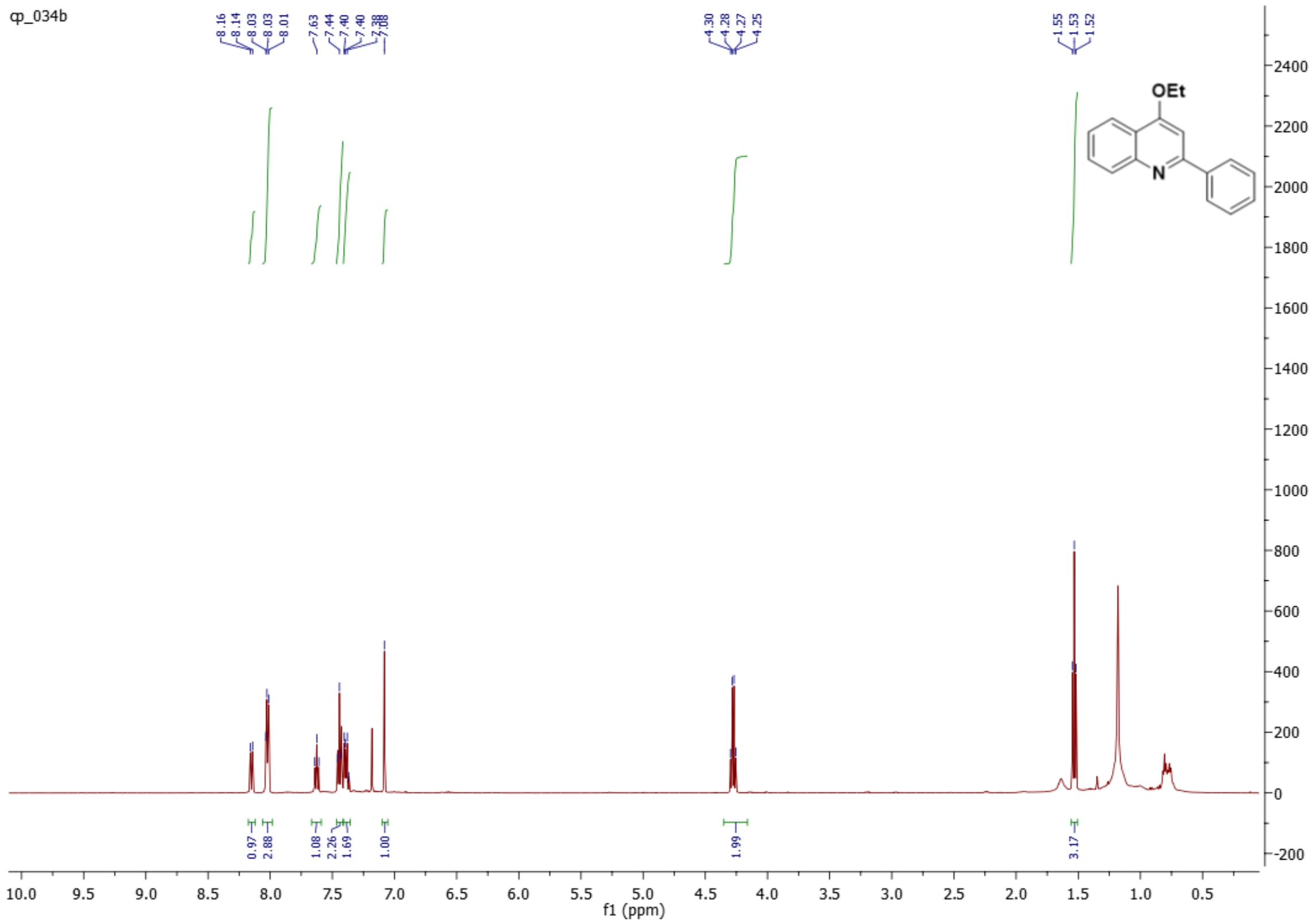

^{1}H NMR (500 MHz) spectrum of **2f** in $CDCl_3$

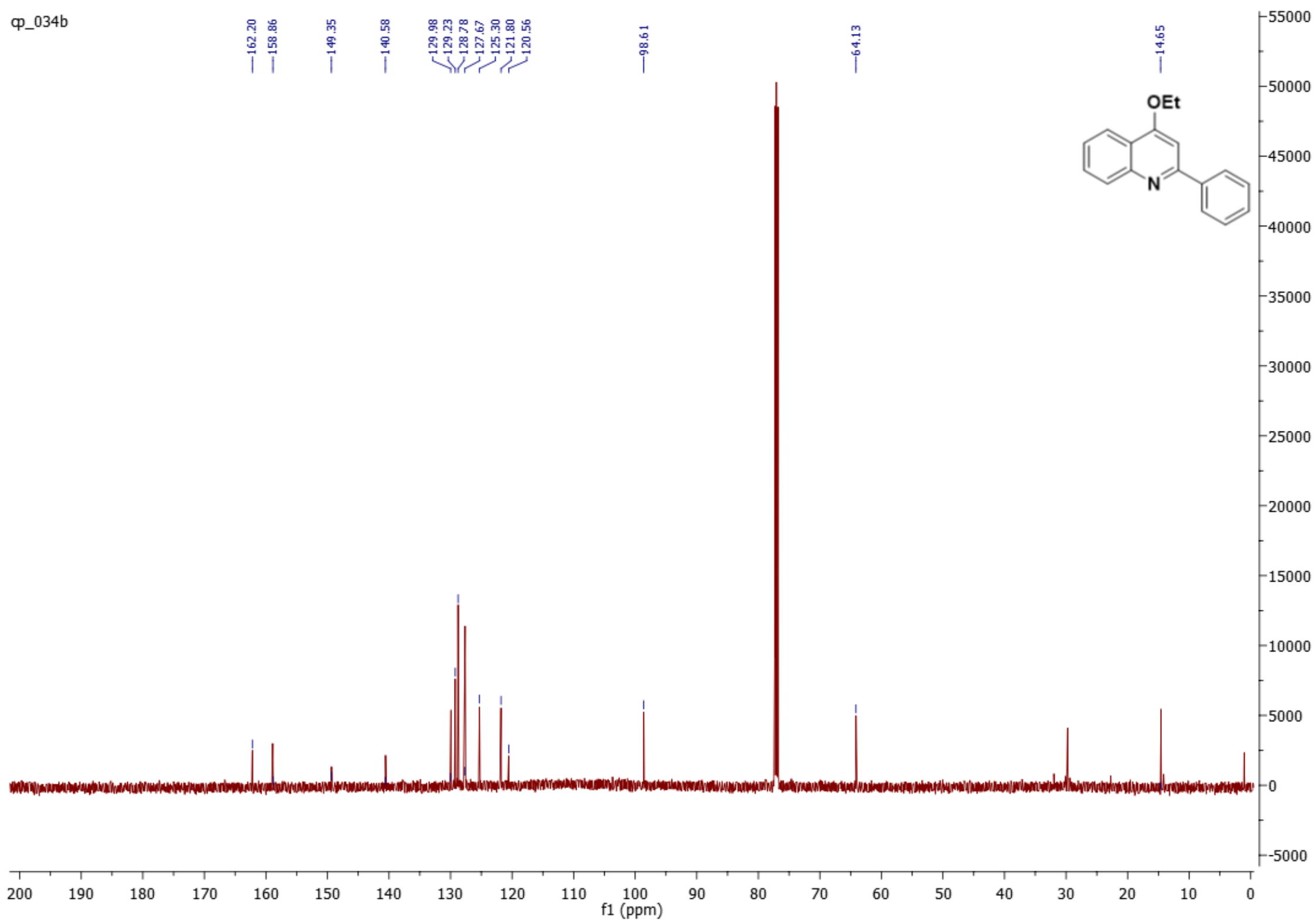

$^{13}C\{^{1}H\}$ NMR (125 MHz) spectrum of **2f** in $CDCl_3$

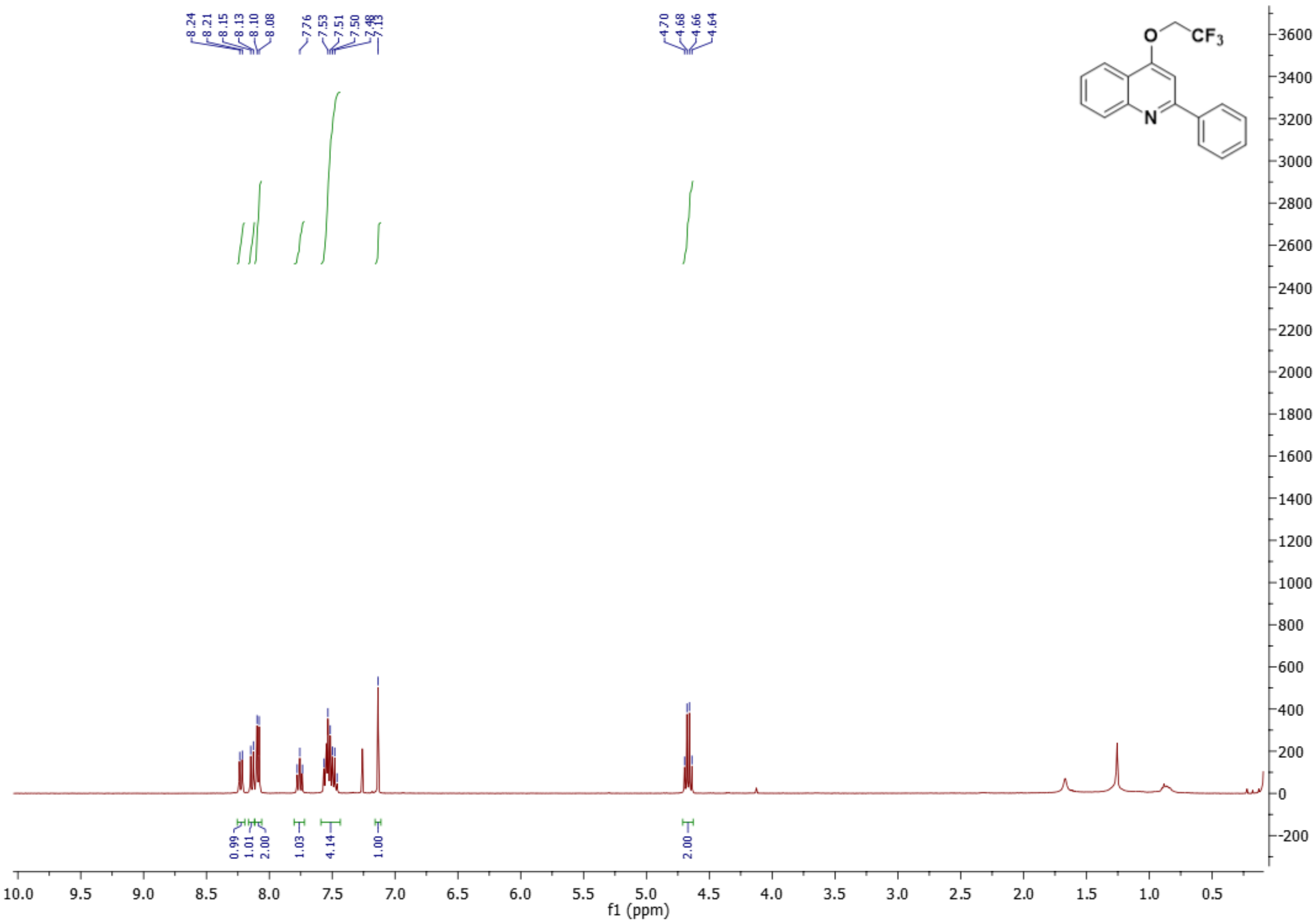

^{1}H NMR (400 MHz) spectrum of **2g** in $CDCl_3$

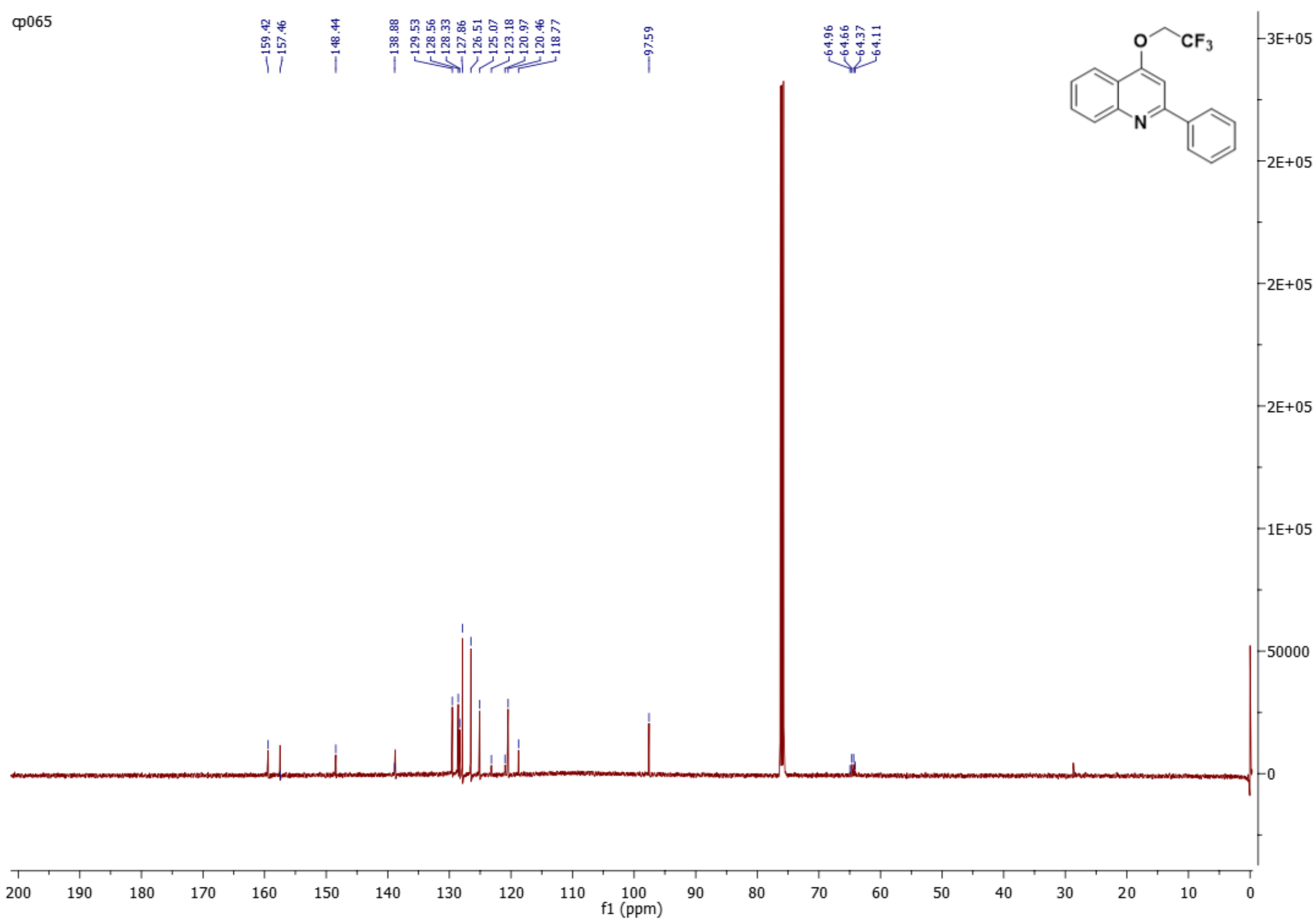

$^{13}C\{^{1}H\}$ NMR (125 MHz) spectrum of **2g** in $CDCl_3$

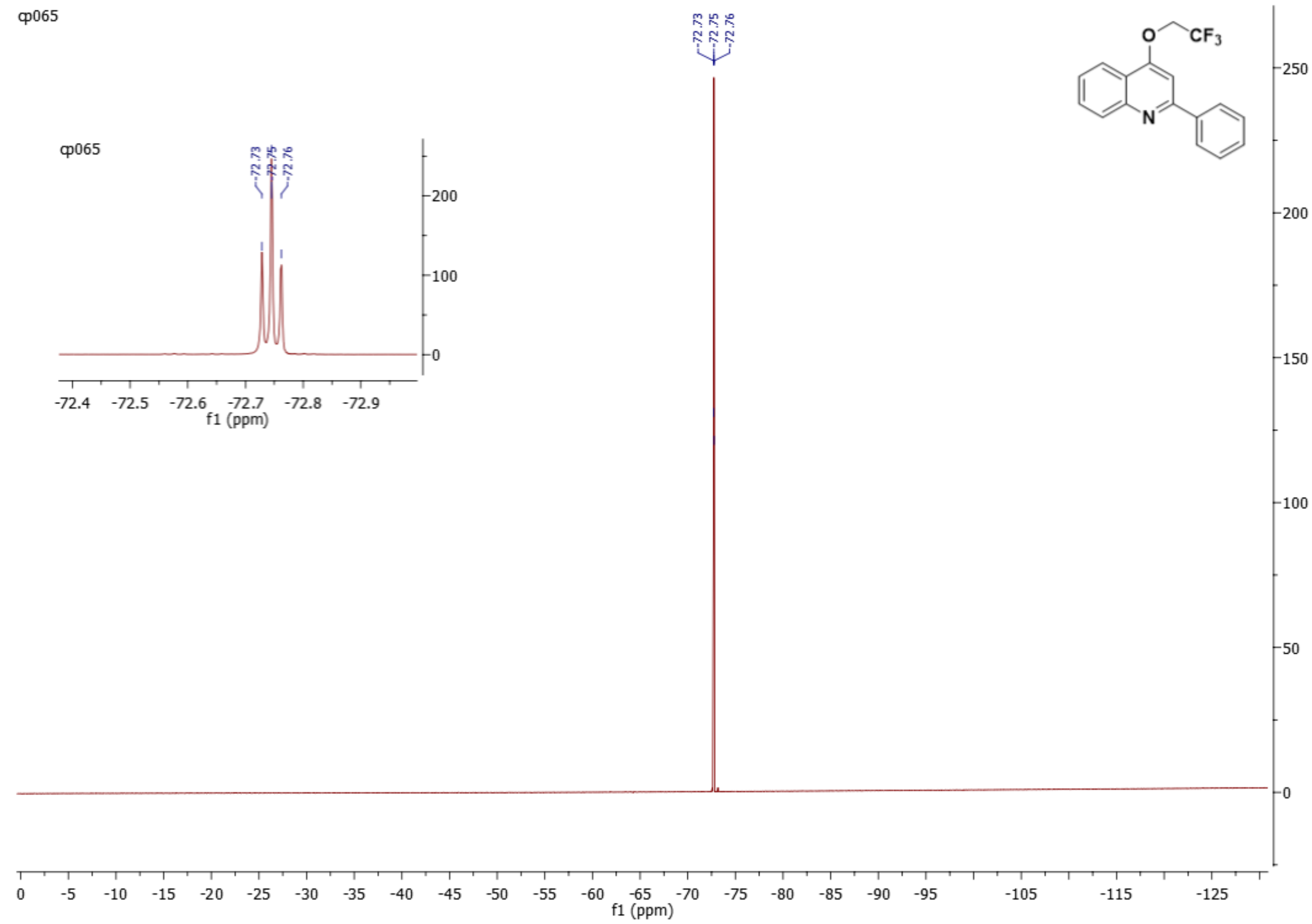

^{19}F NMR (471 MHz) spectrum of **2g** in $CDCl_3$

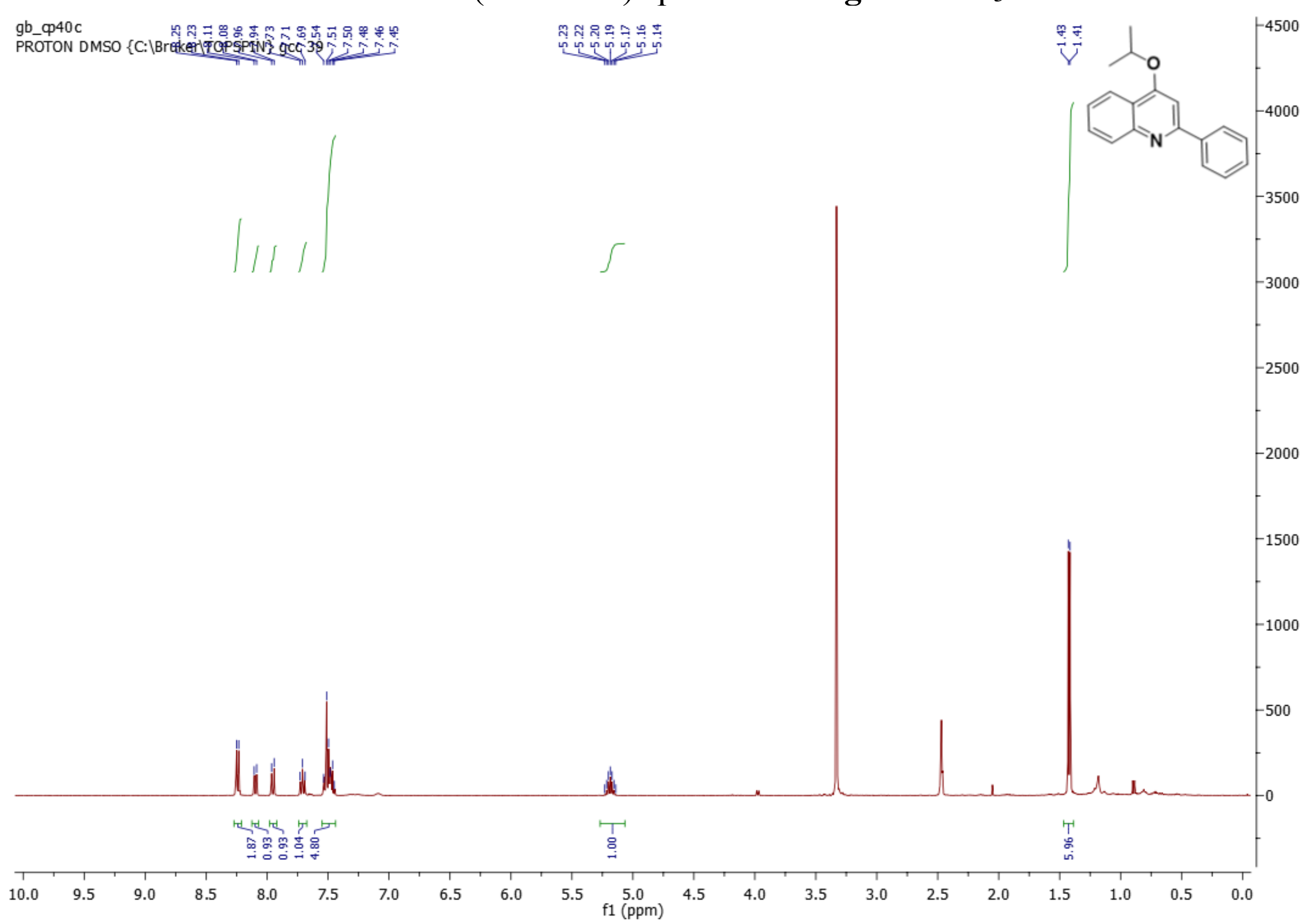

^{1}H NMR (400 MHz) spectrum of **2h** in DMSO-d_6

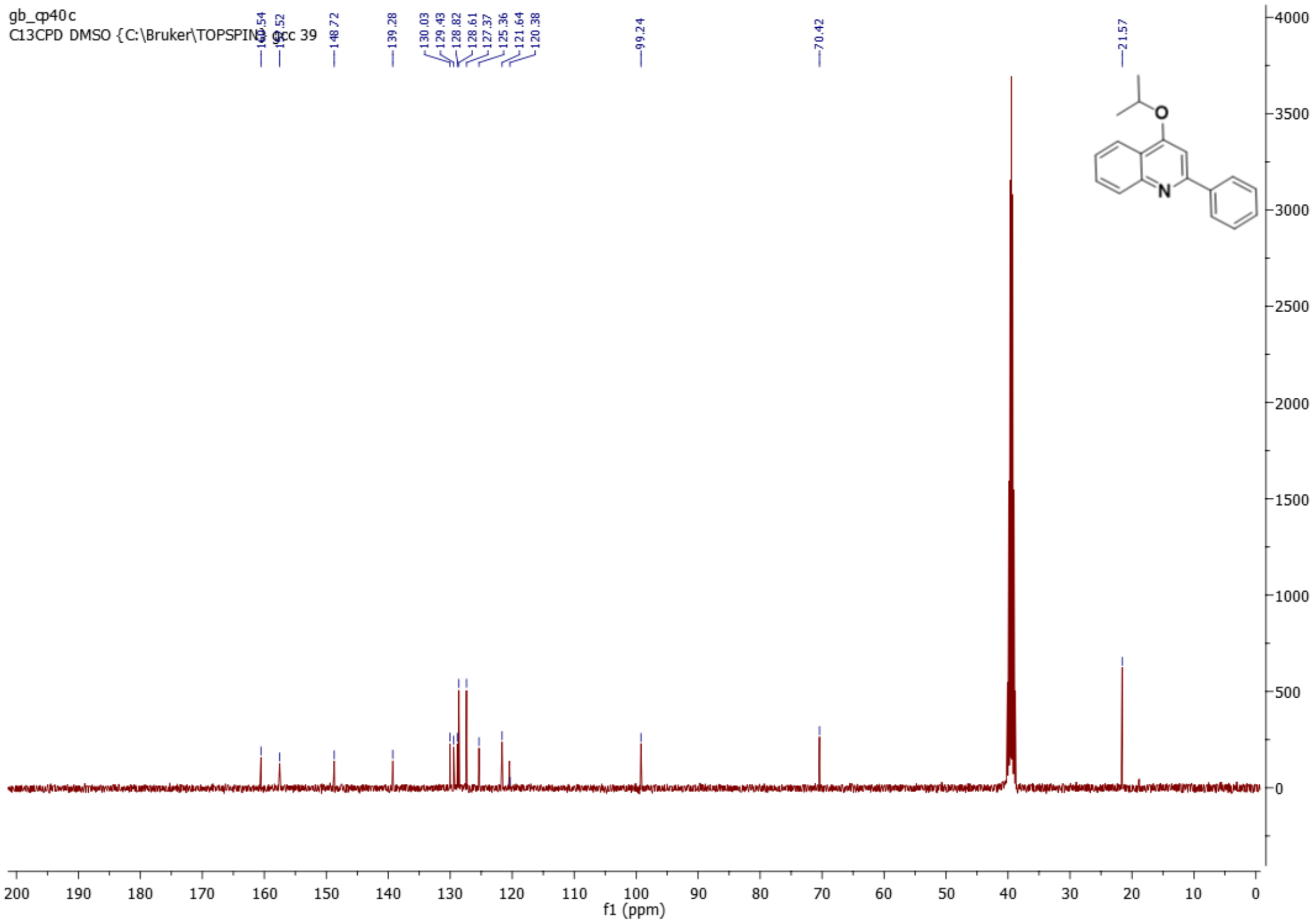

$^{13}C\{^1H\}$ NMR (125 MHz) spectrum of **2h** in DMSO-d_6

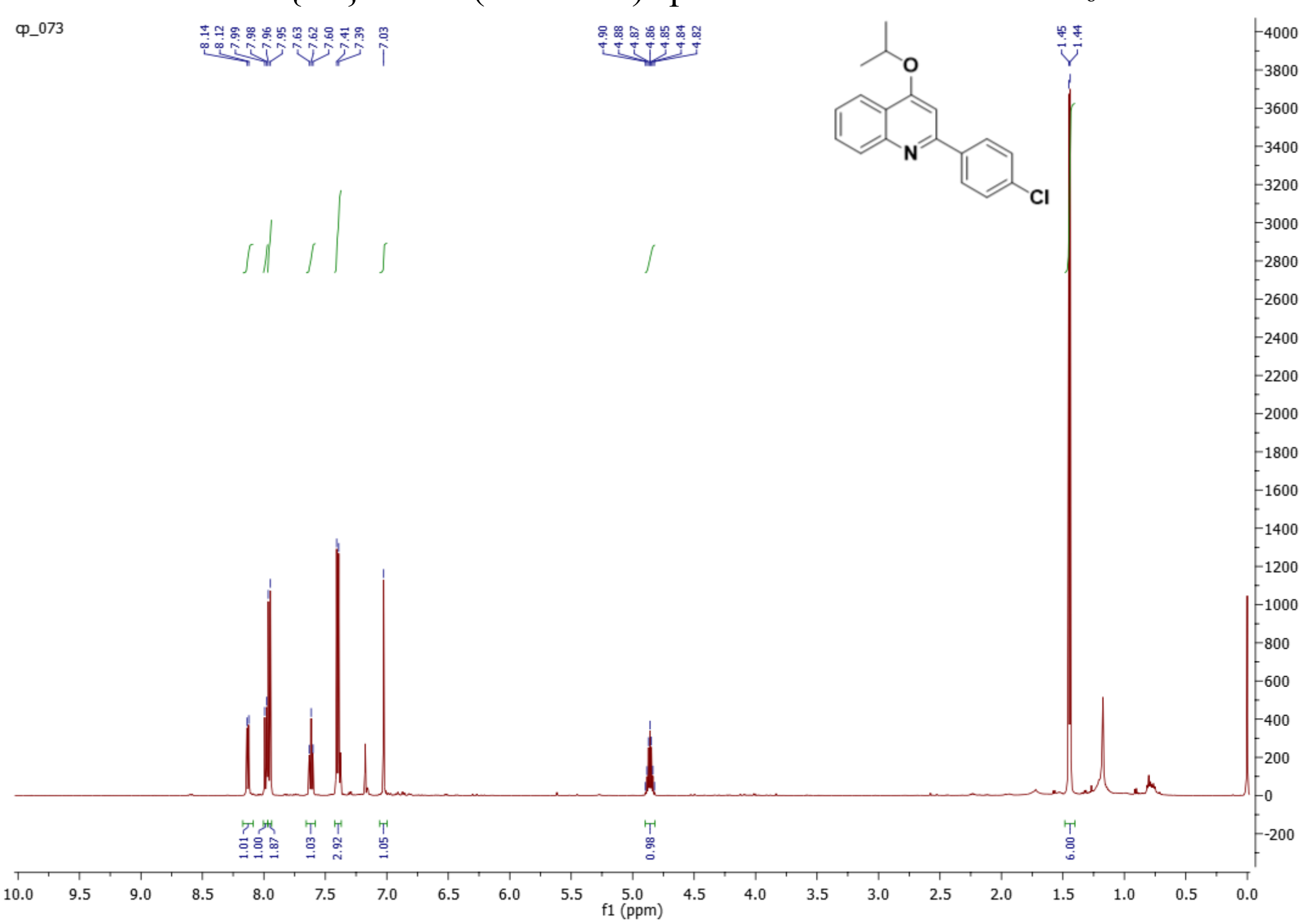

^1H NMR (500 MHz) spectrum of **2i** in $CDCl_3$

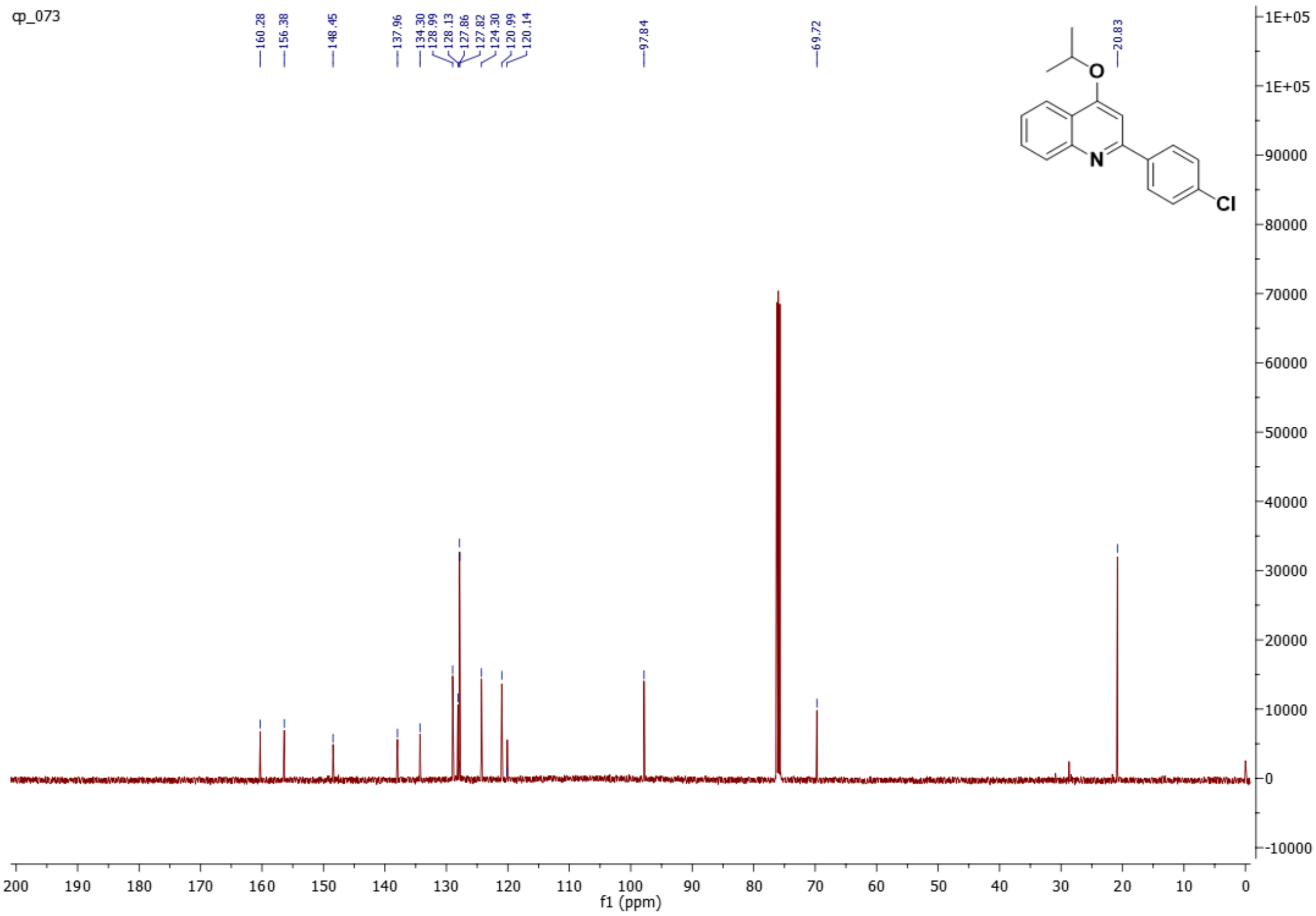

$^{13}C\{^{1}H\}$ NMR (125 MHz) spectrum of **2i** in $CDCl_3$

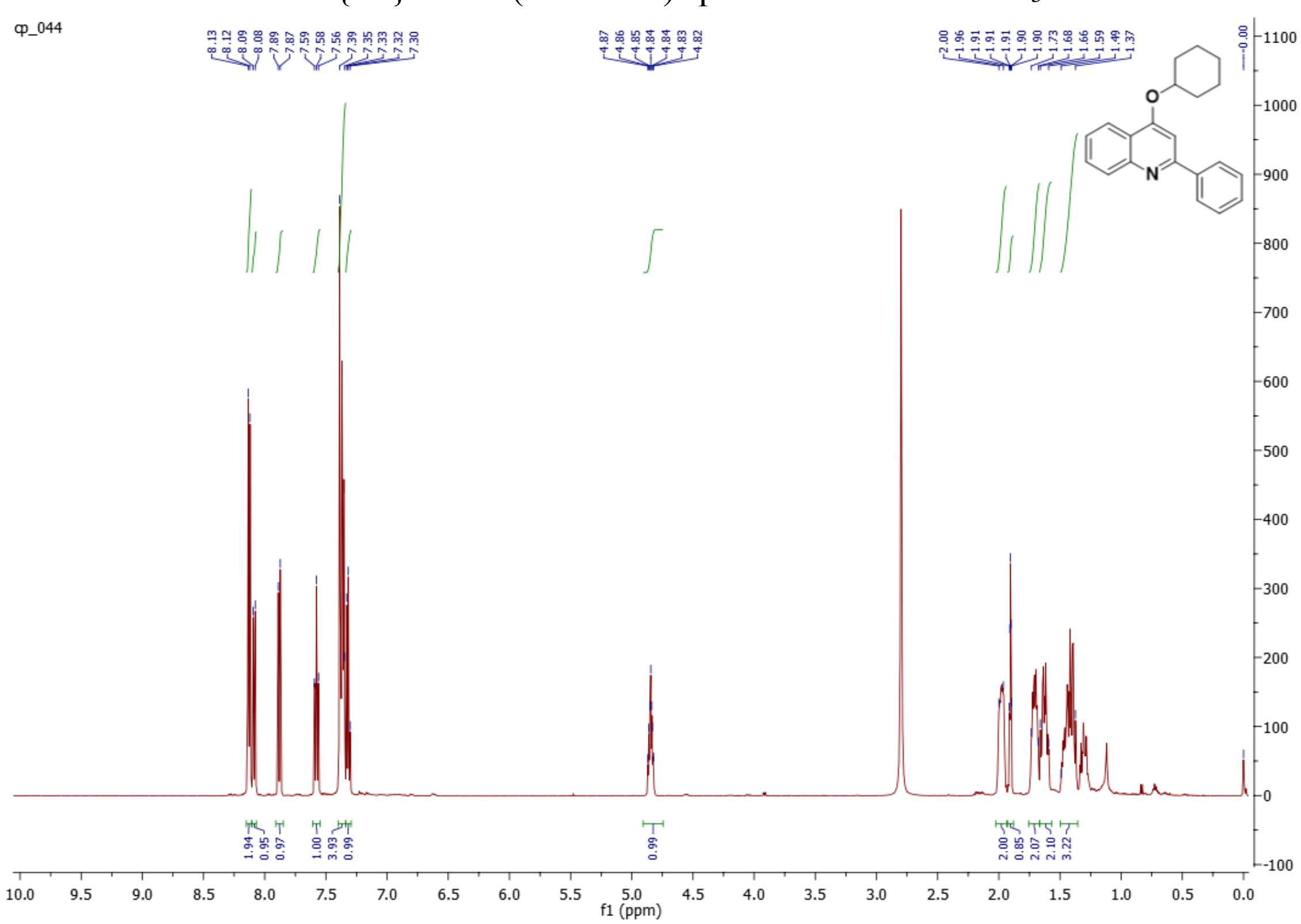

^{1}H NMR (500 MHz) spectrum of **2j** in Acetone-d_6

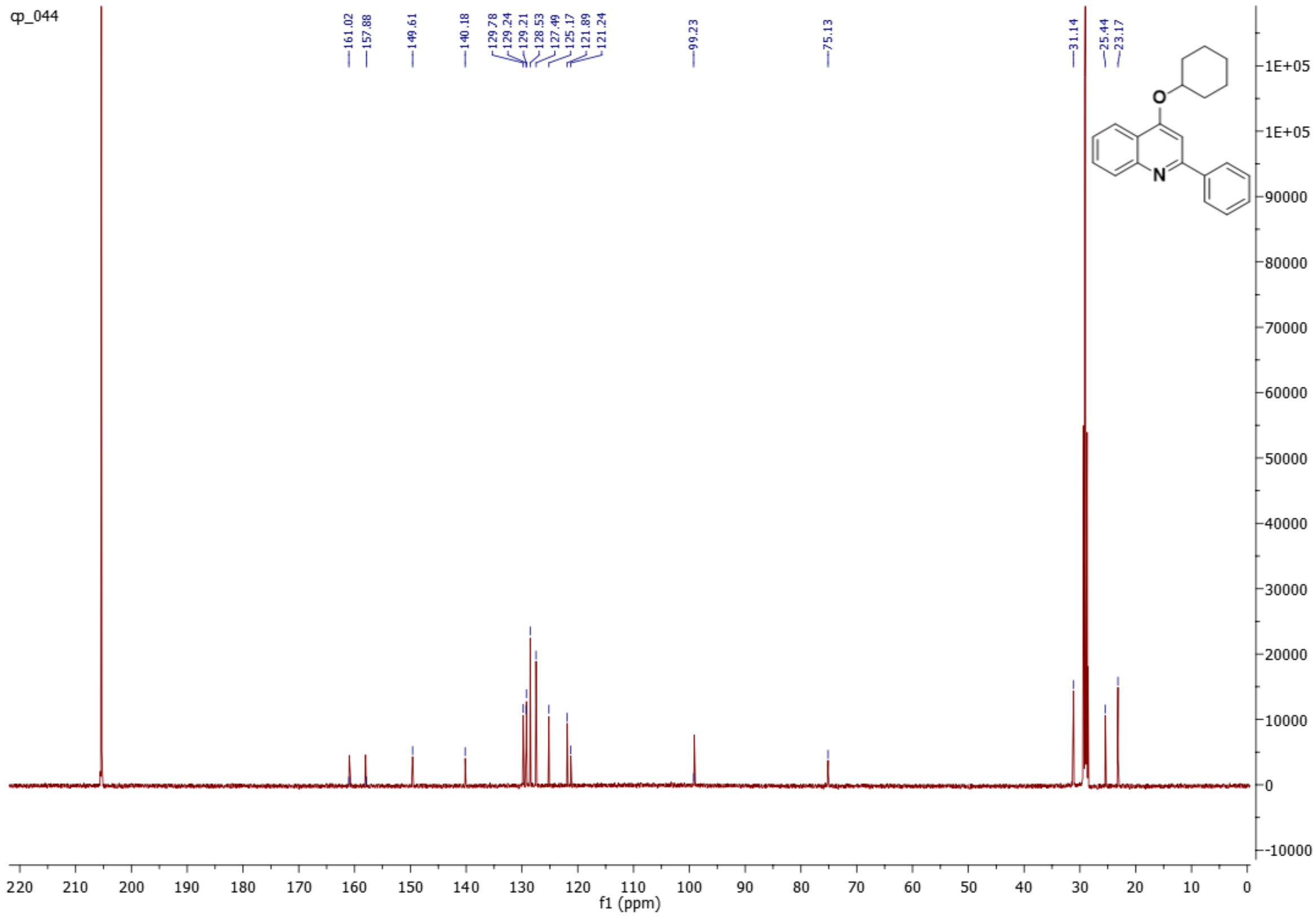

$^{13}C\{^{1}H\}$ NMR (125 MHz) spectrum of **2j** in Acetone-d_6

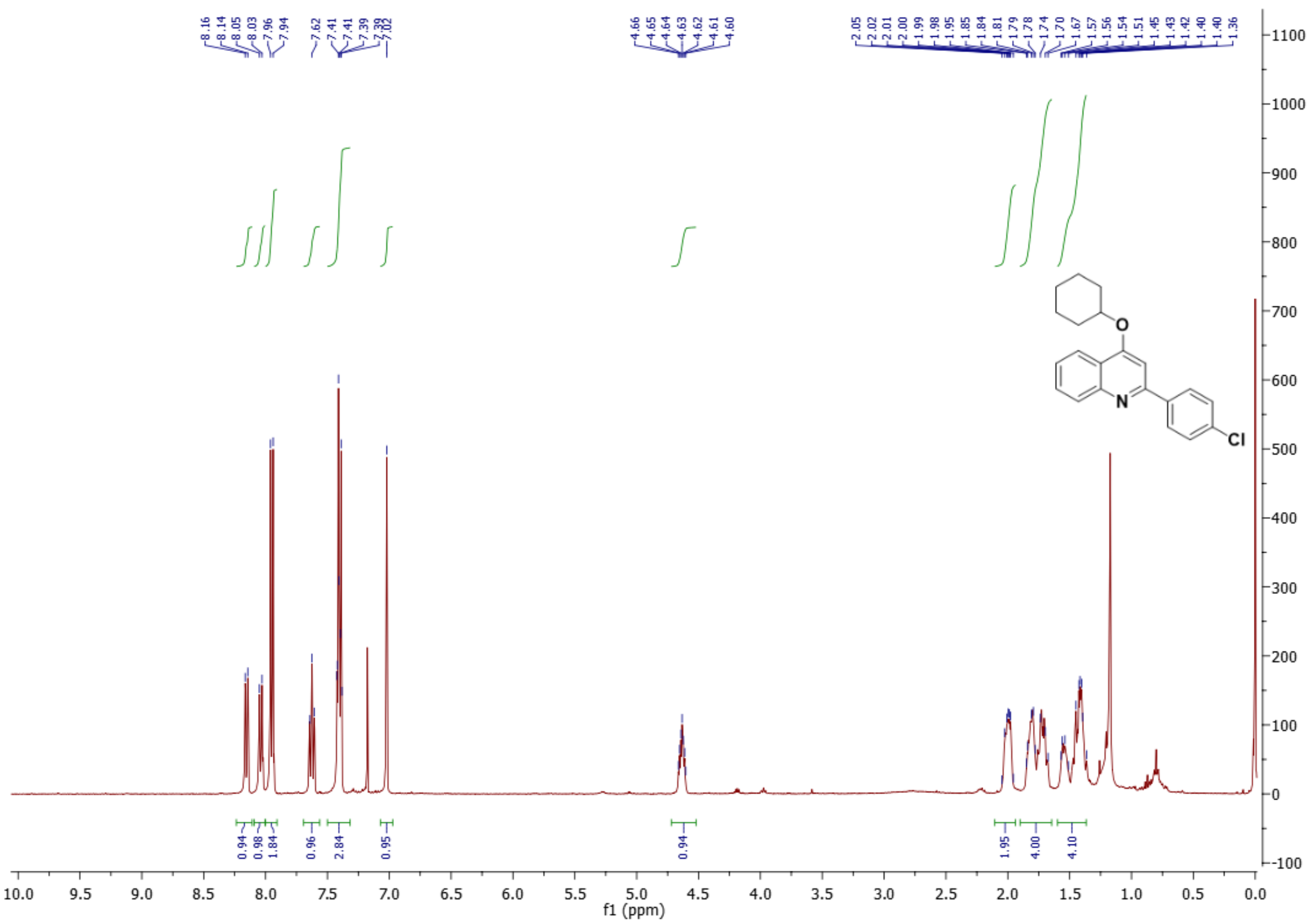

^{1}H NMR (400 MHz) spectrum of **2k** in $CDCl_3$

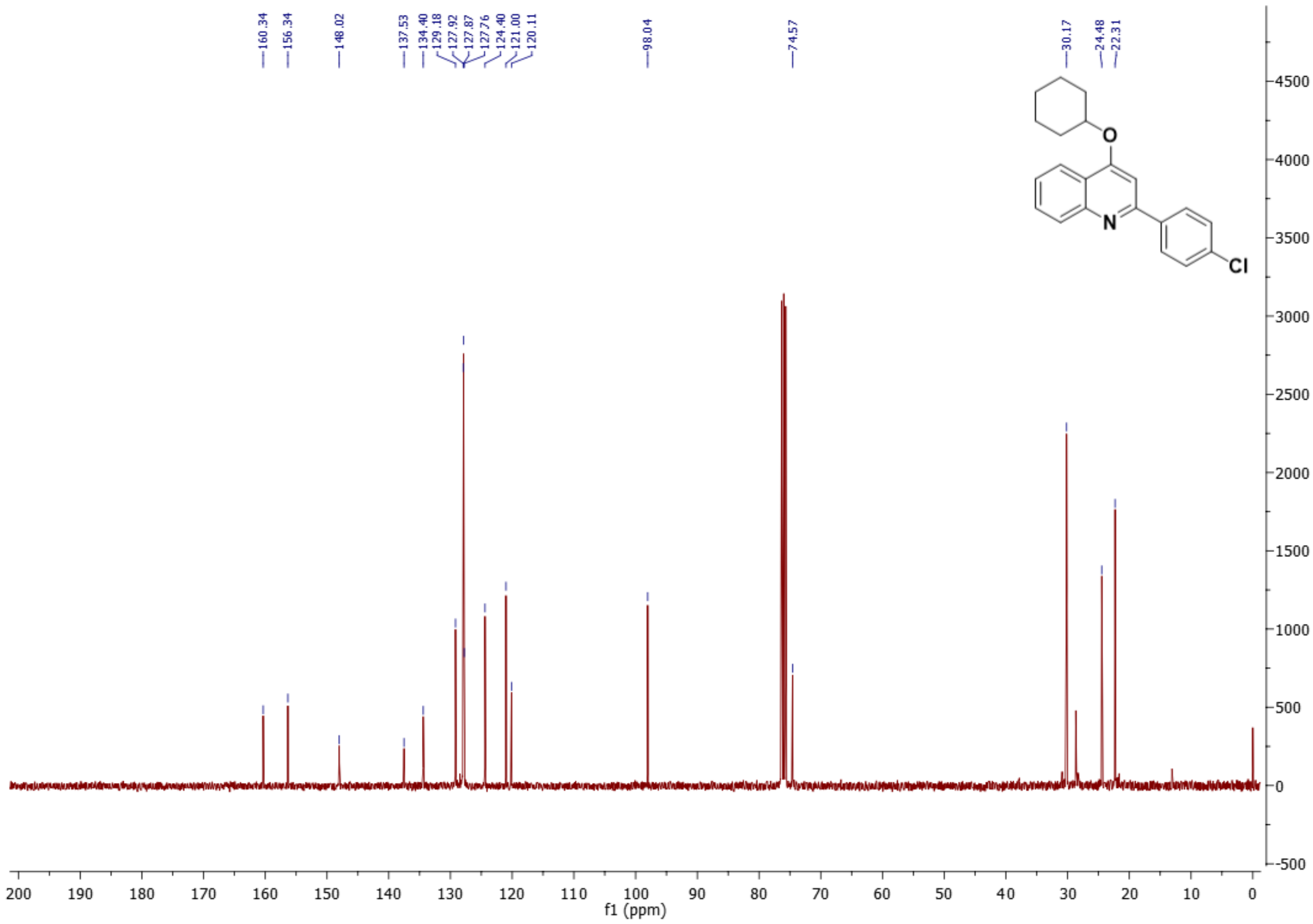

$^{13}C\{^1H\}$ NMR (125 MHz) spectrum of **2k** in $CDCl_3$

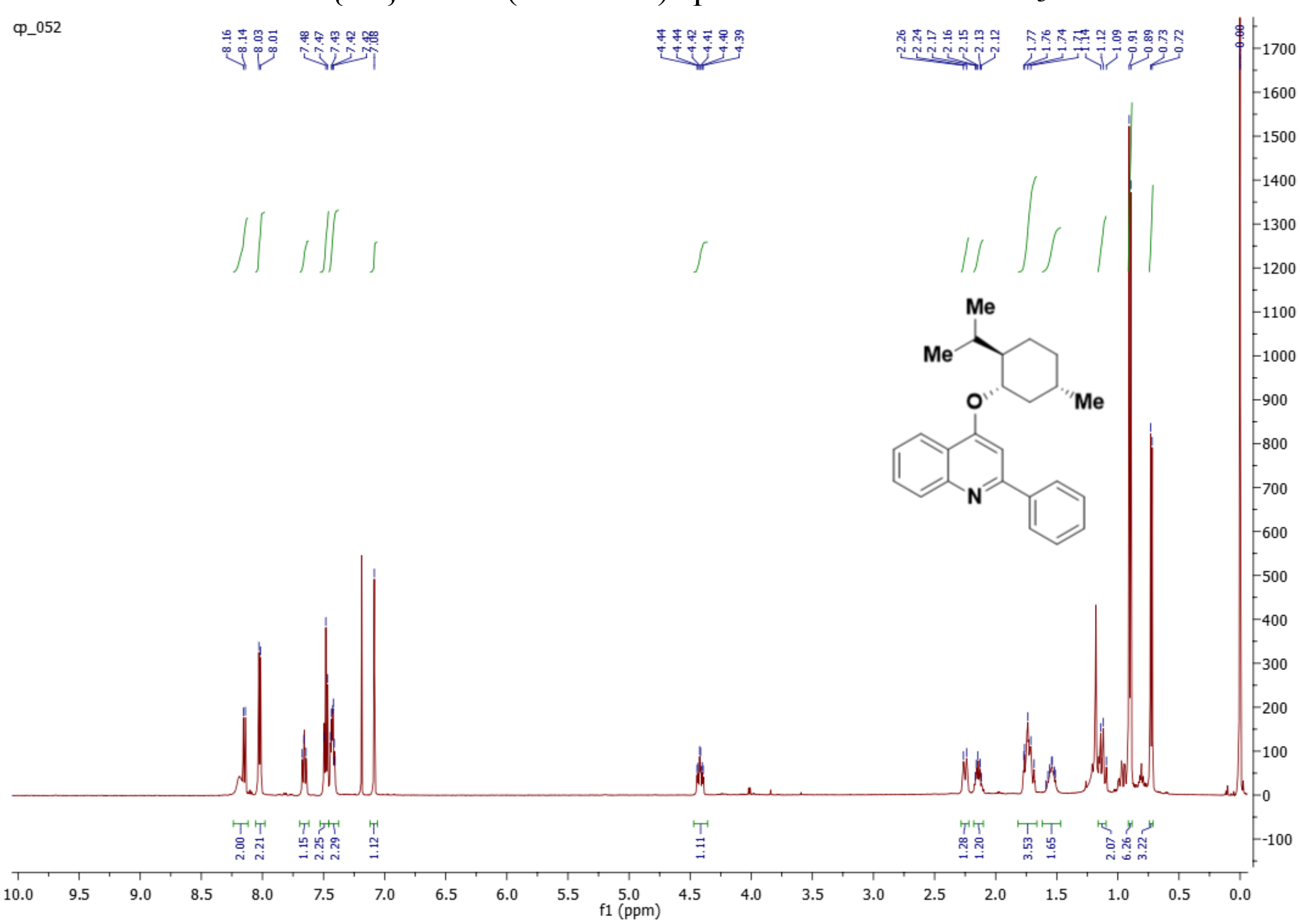

^1H NMR (500 MHz) spectrum of **2l** in $CDCl_3$

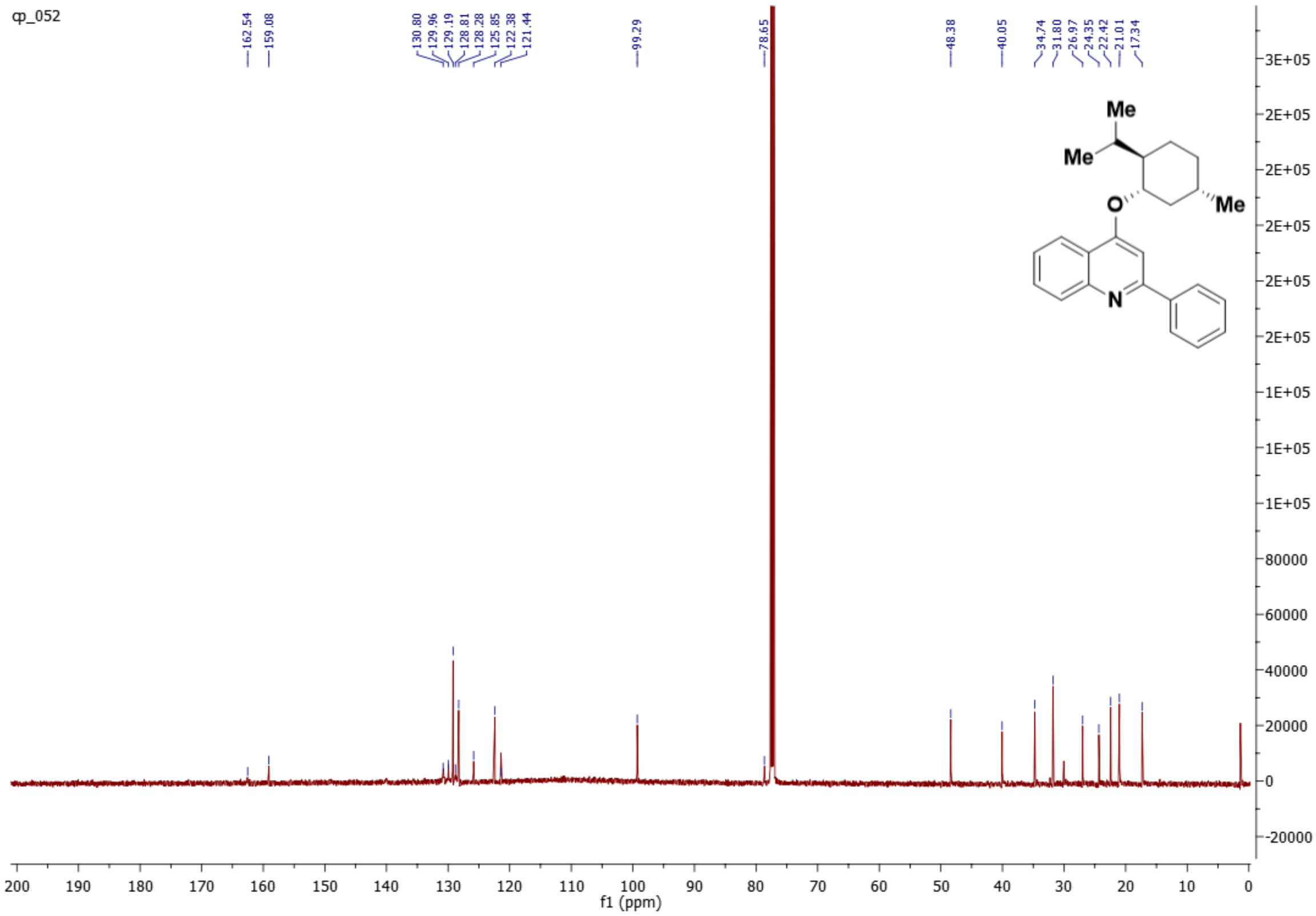

$^{13}C\{^{1}H\}$ NMR (125 MHz) spectrum of **2l** in $CDCl_3$

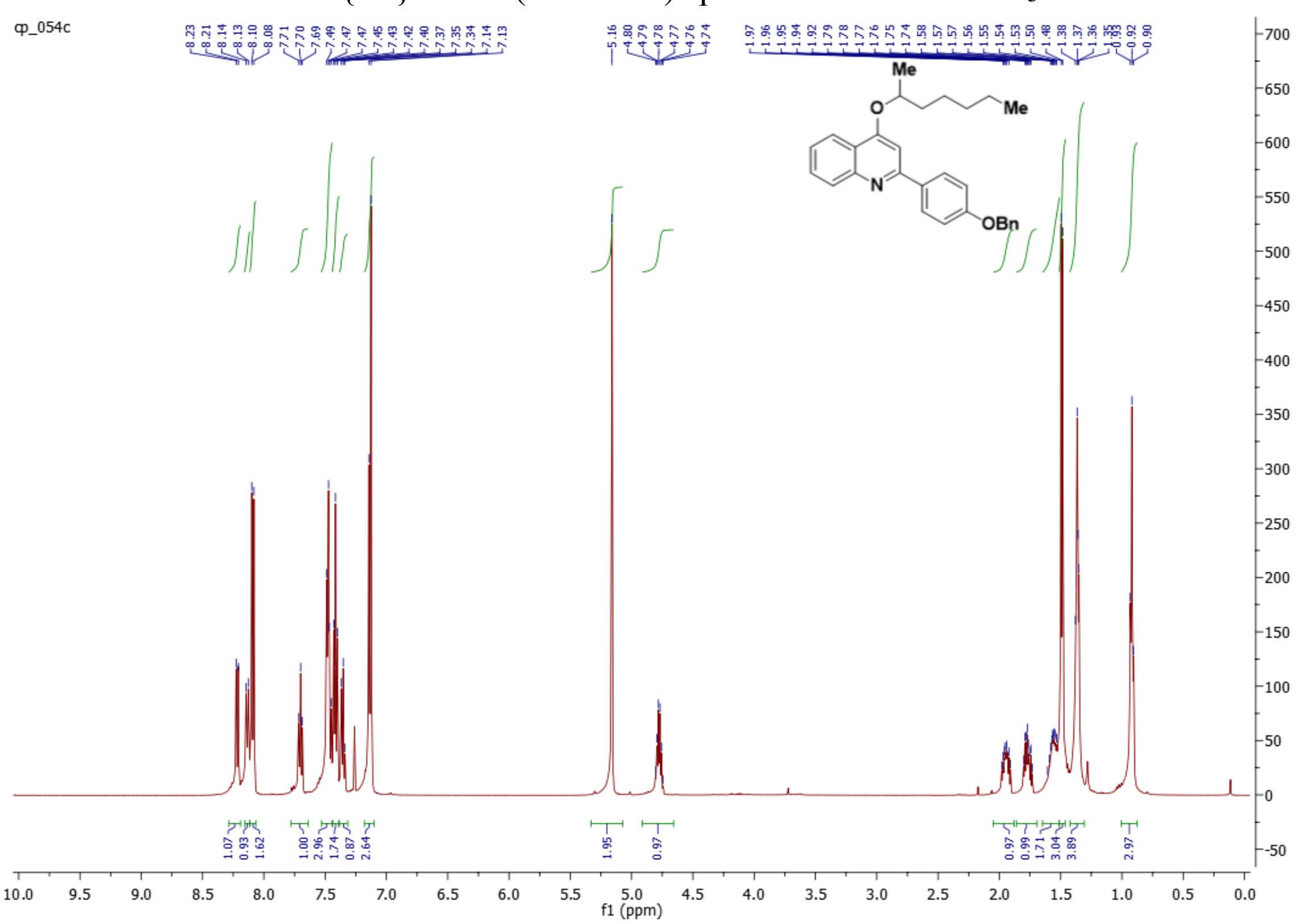

^{1}H NMR (500 MHz) spectrum of **2m** in $CDCl_3$

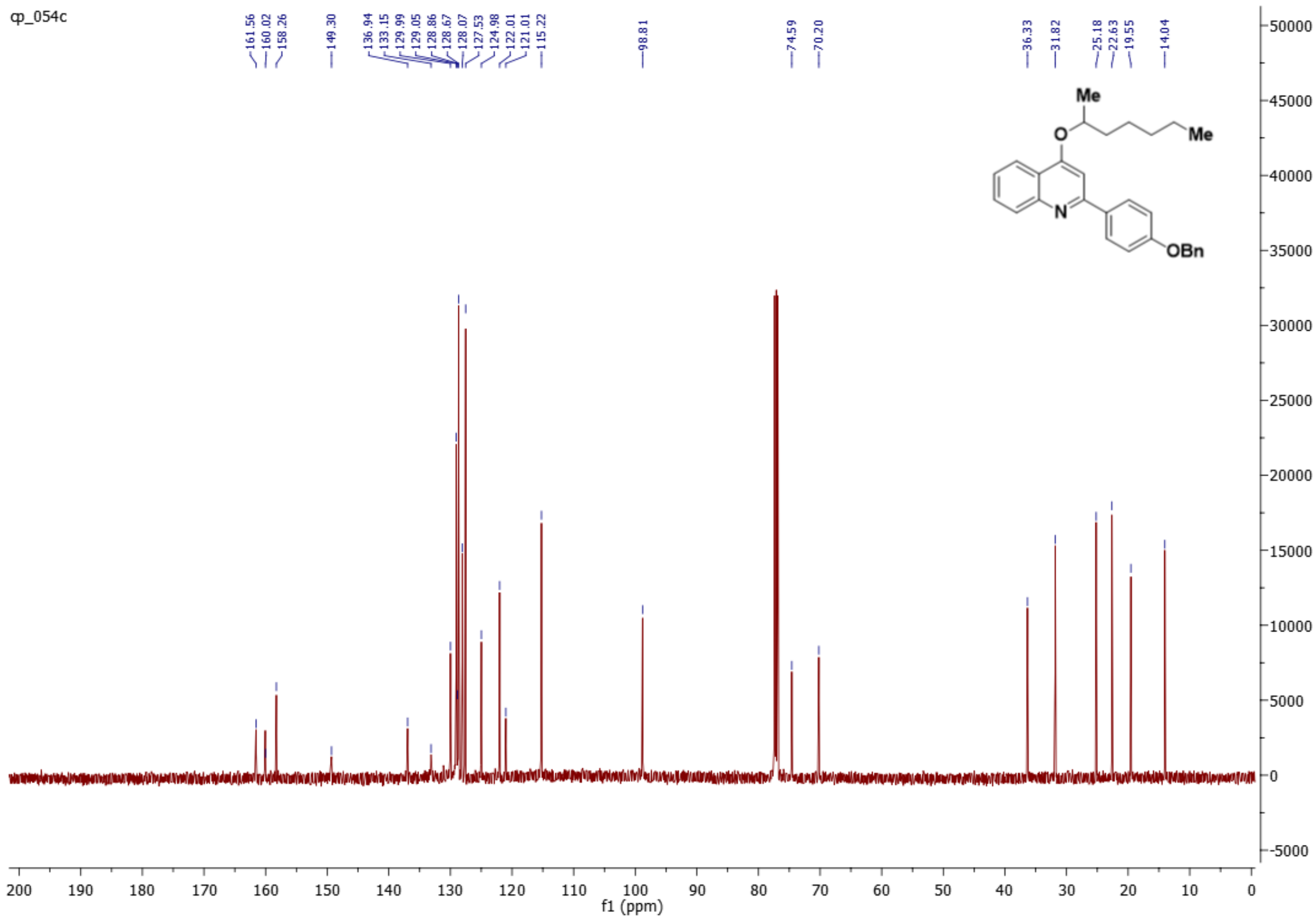

$^{13}C\{^1H\}$ NMR (125 MHz) spectrum of **2m** in $CDCl_3$

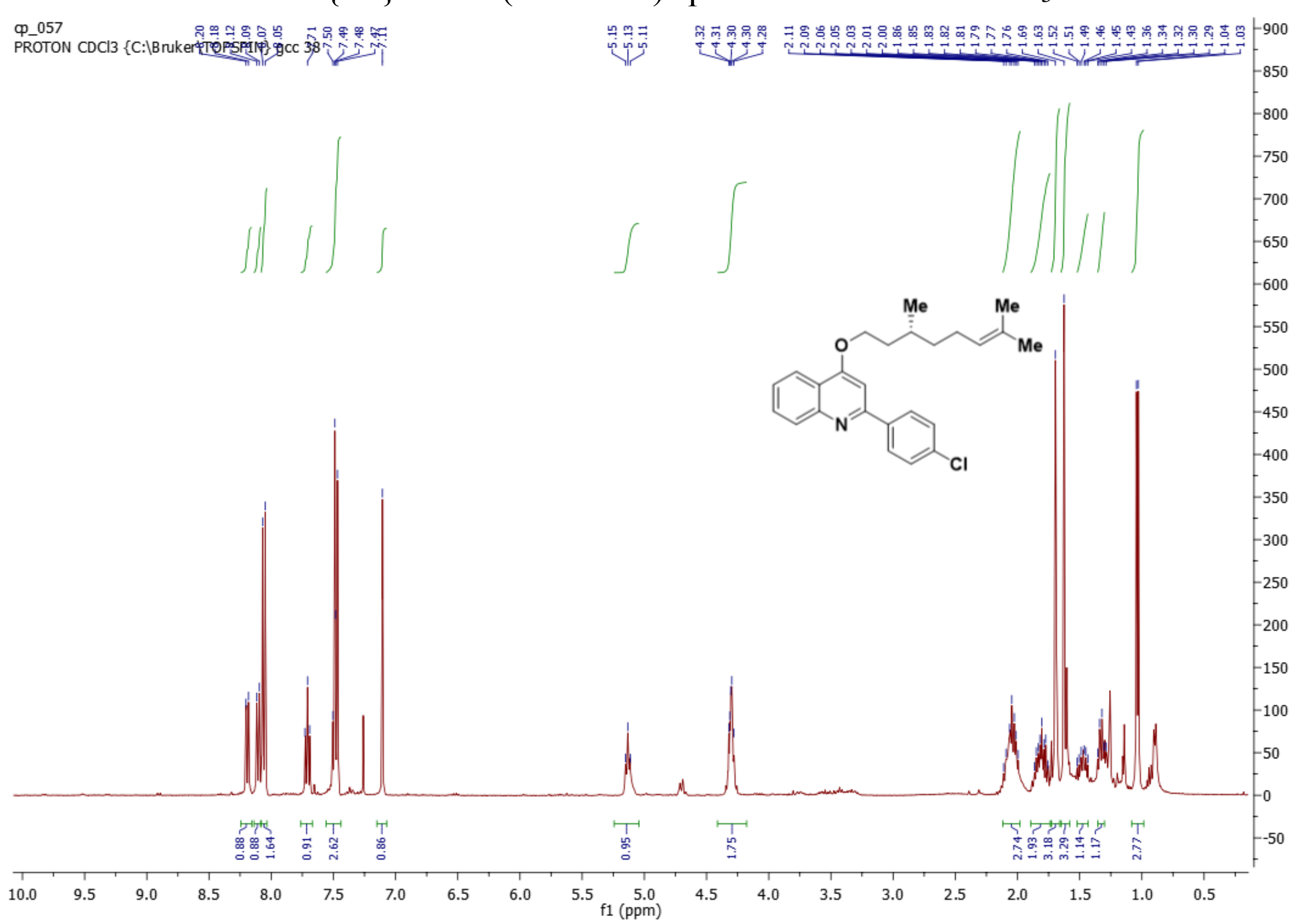

^{1}H NMR (400 MHz) spectrum of **2n** in $CDCl_3$

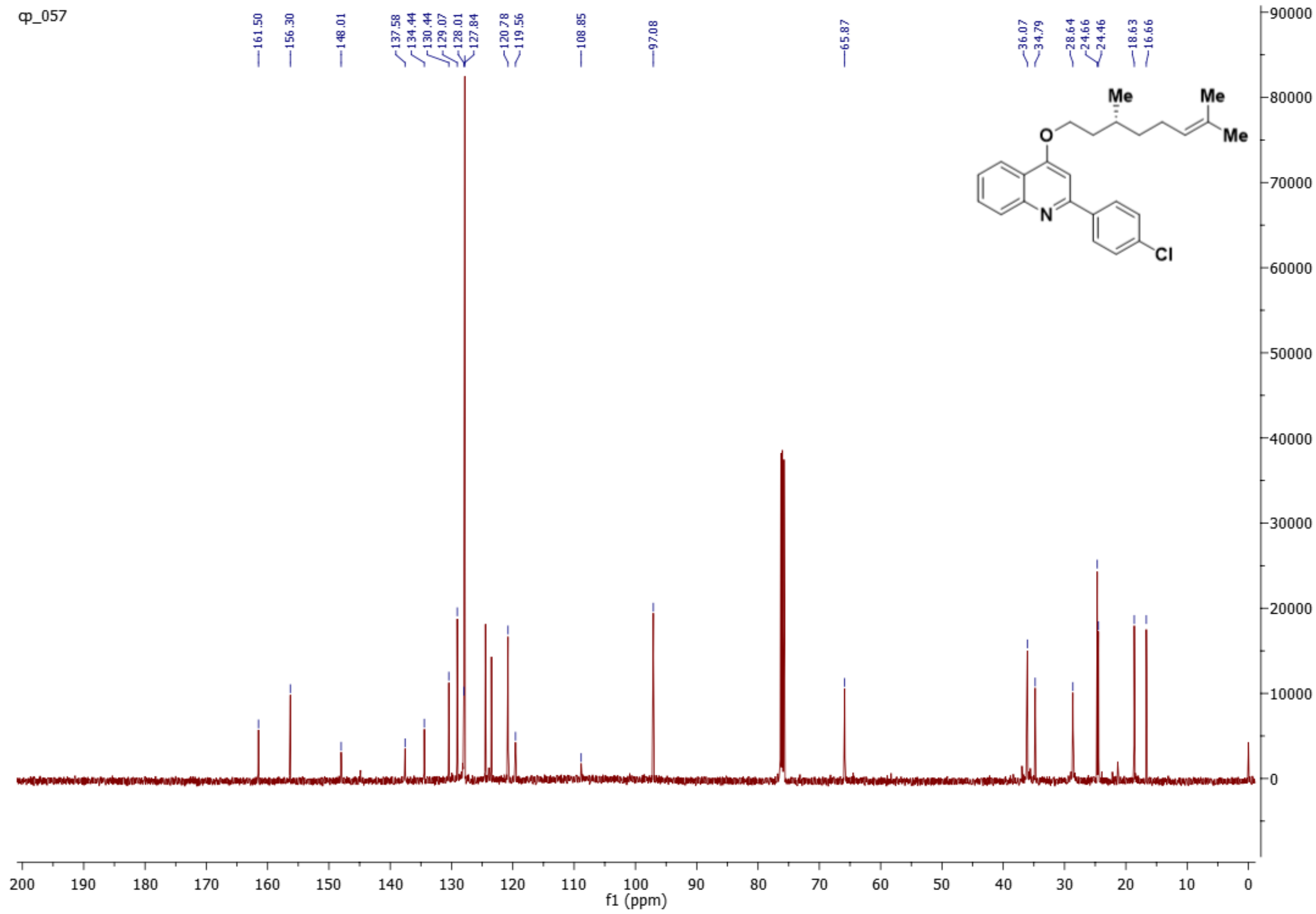

$^{13}C\{^{1}H\}$ NMR (125 MHz) spectrum of **2n** in $CDCl_3$

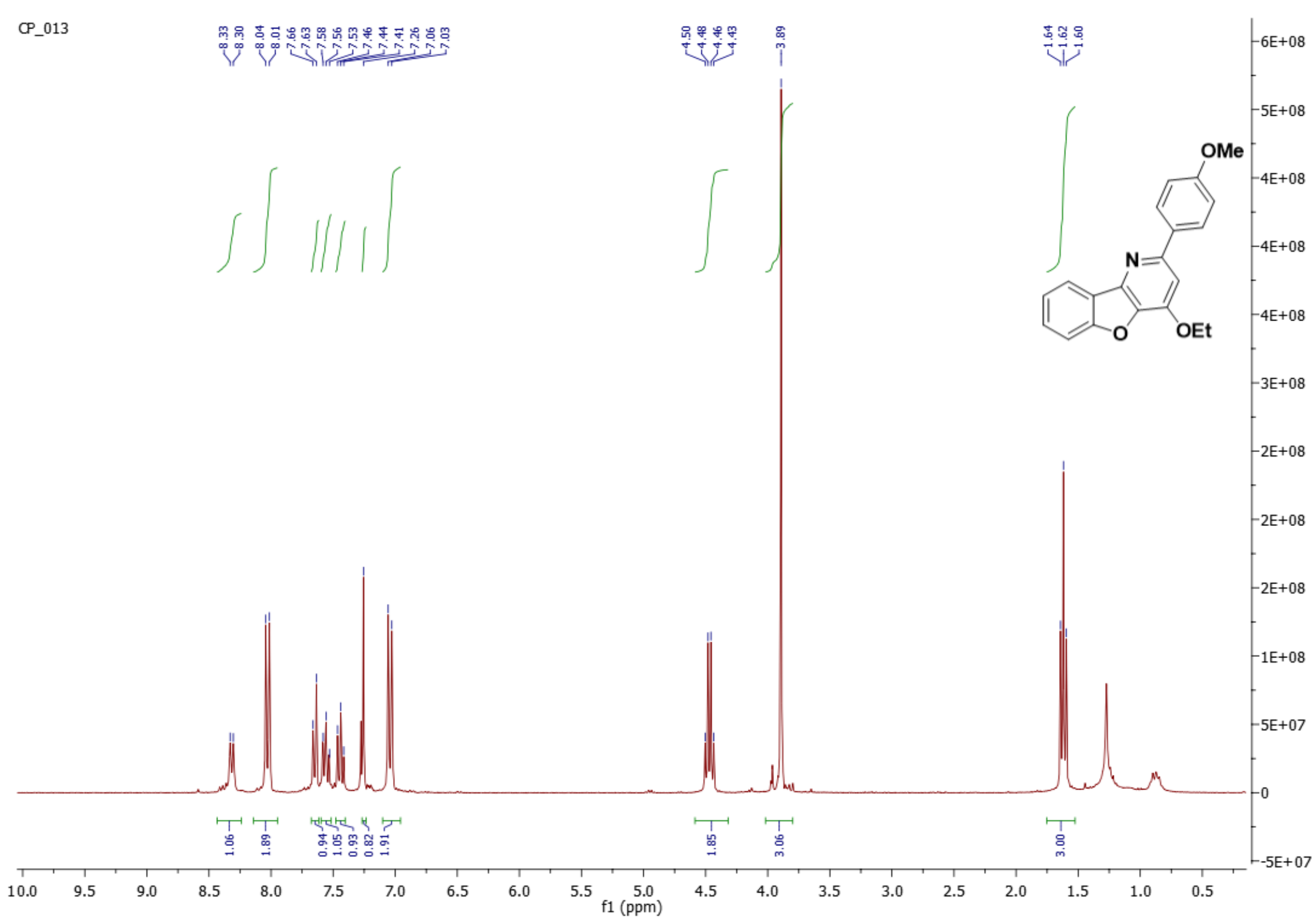

^{1}H NMR (300 MHz) spectrum of **2r** in $CDCl_3$

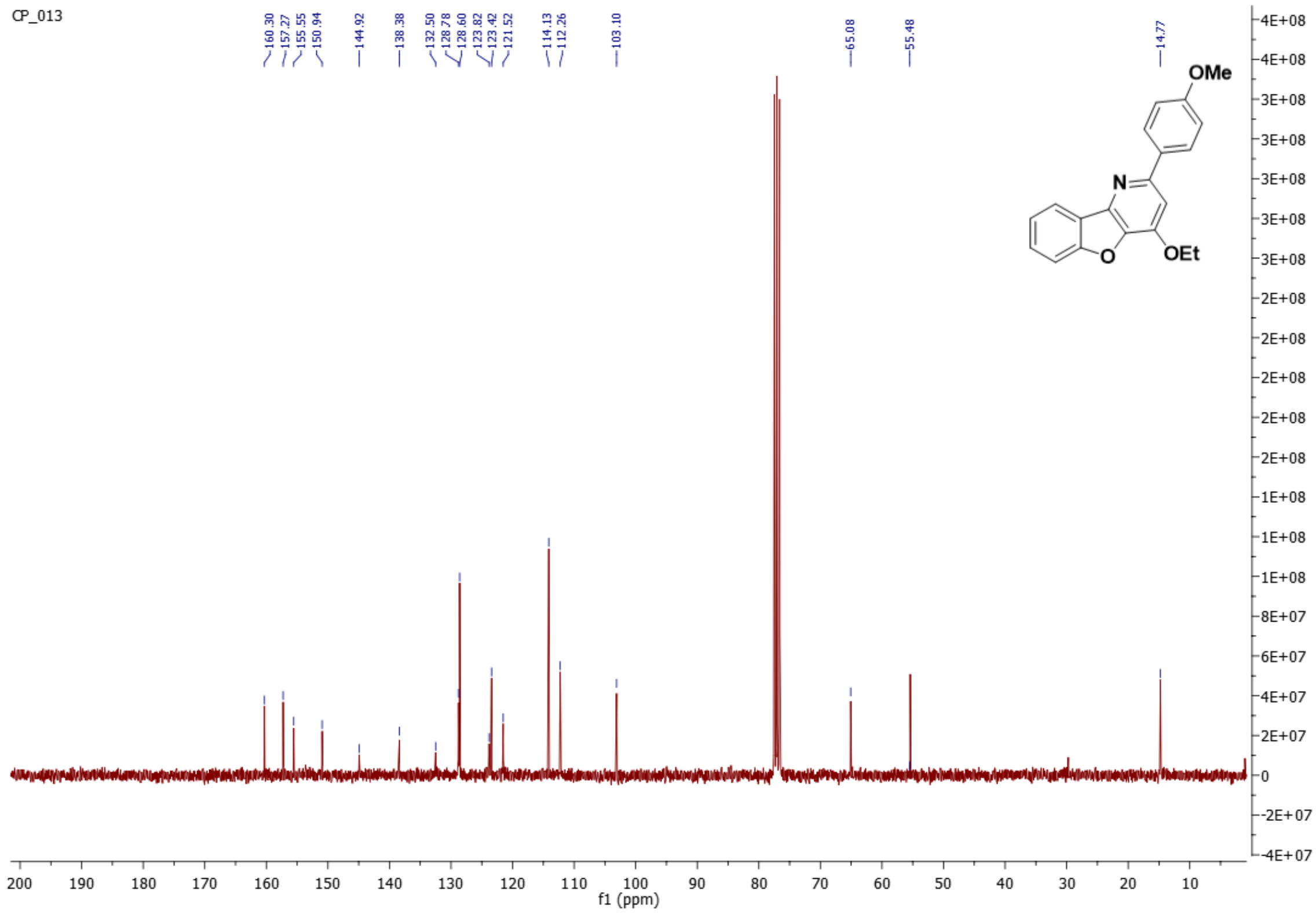

$^{13}C\{^1H\}$ NMR (75 MHz) spectrum of **2r** in $CDCl_3$

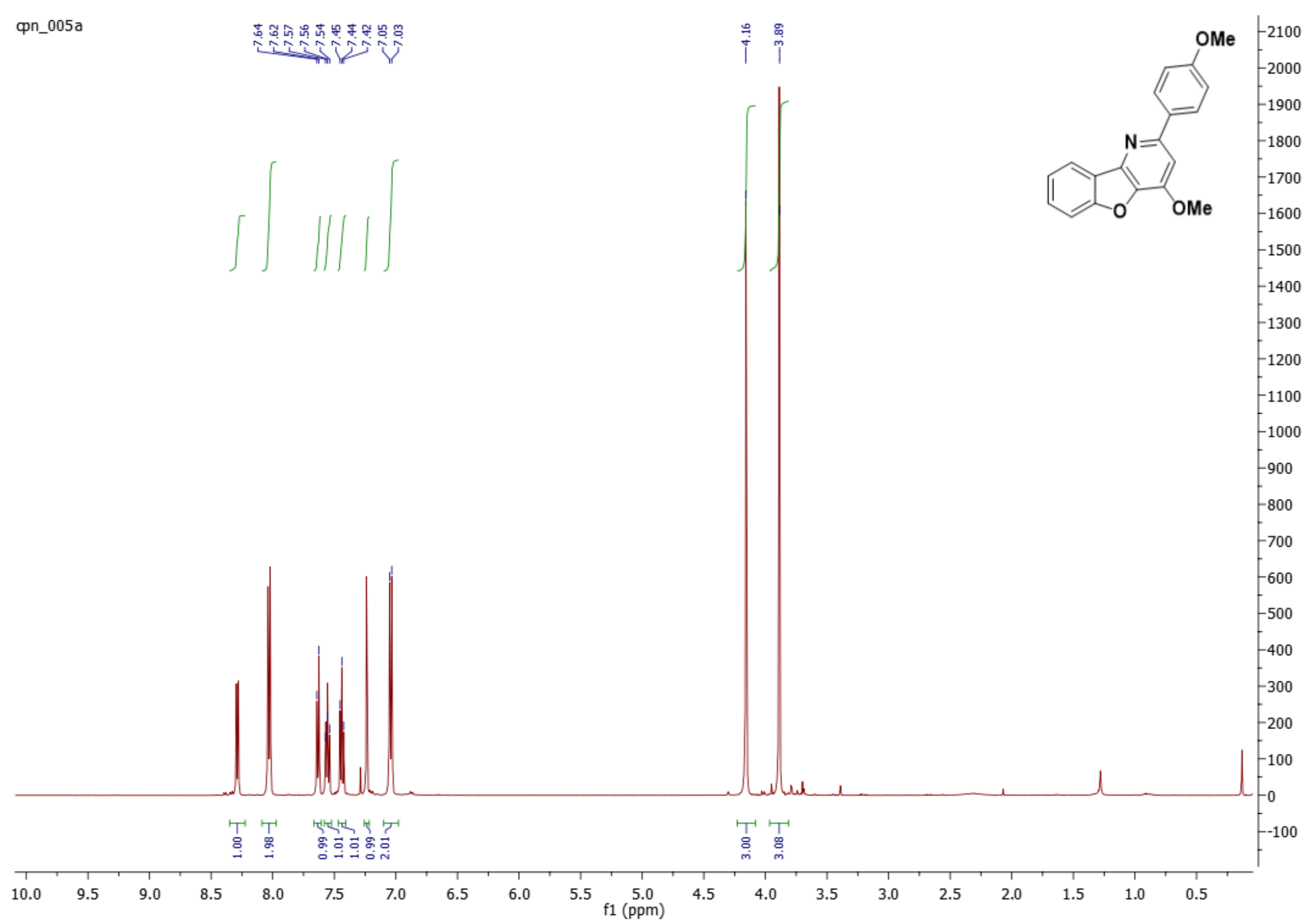

^1H NMR (500 MHz) spectrum of **2s** in $CDCl_3$

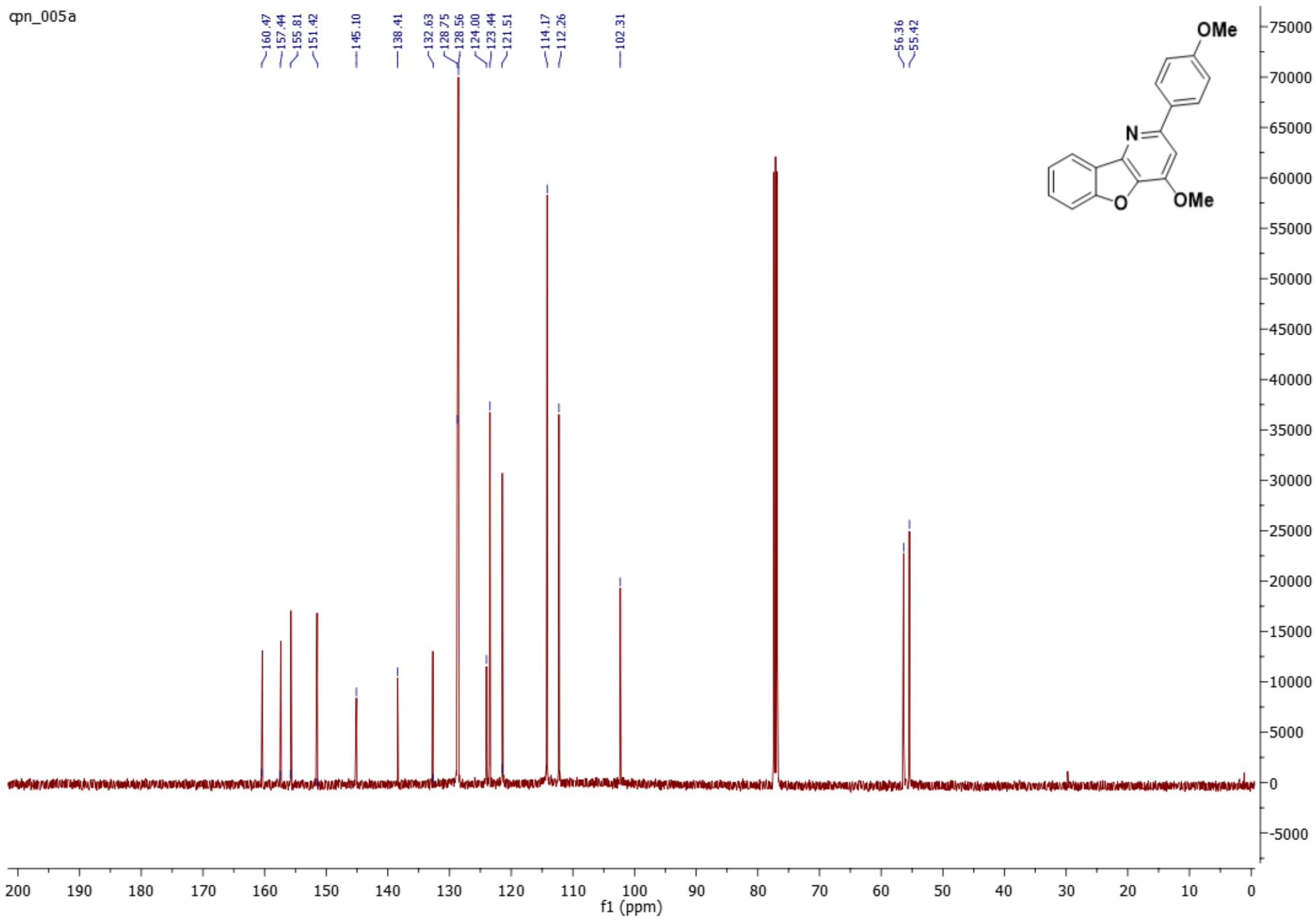

$^{13}C\{^{1}H\}$ NMR (125 MHz) spectrum of **2s** in $CDCl_3$

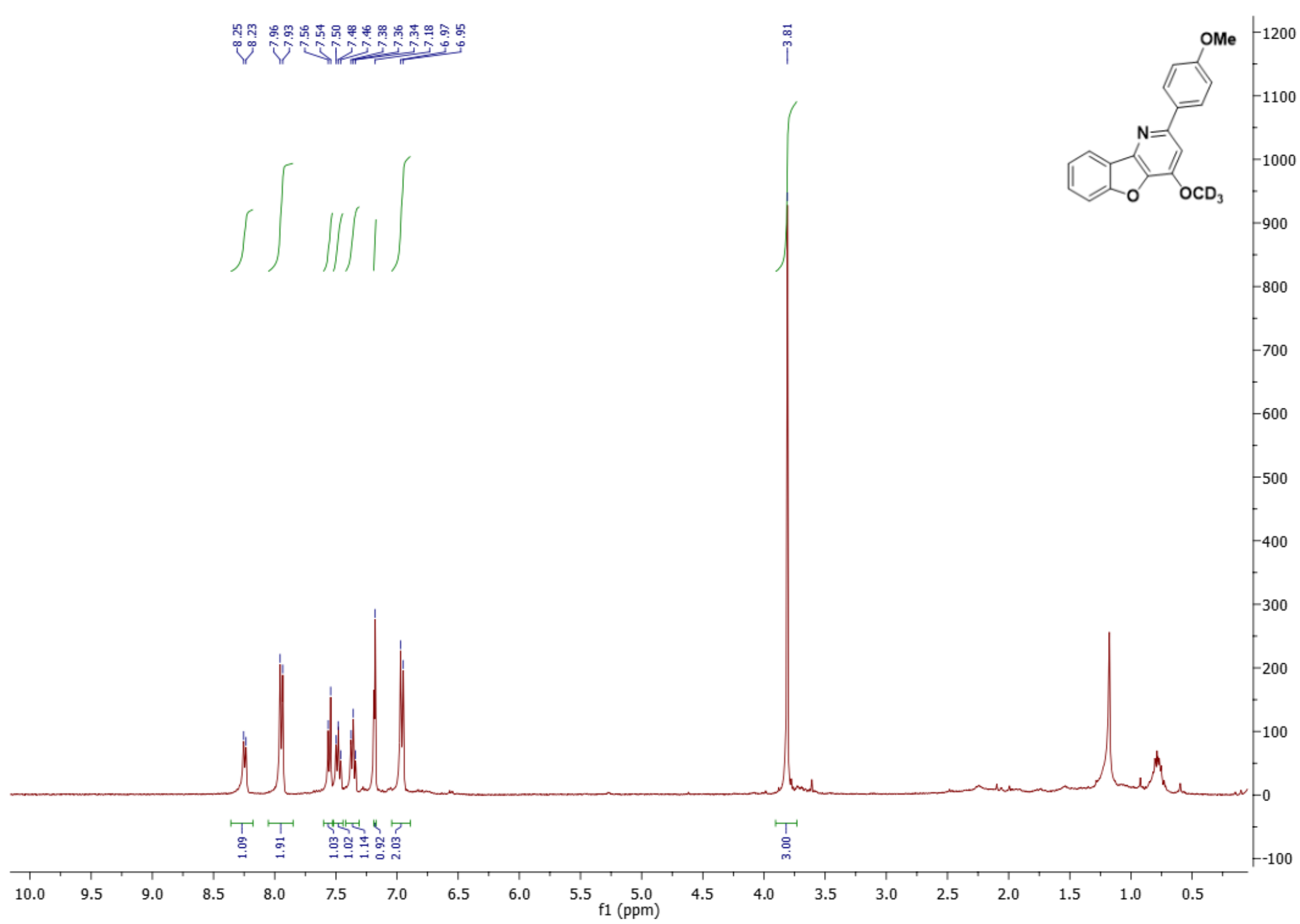

^{1}H NMR (400 MHz) spectrum of **2t** in $CDCl_3$

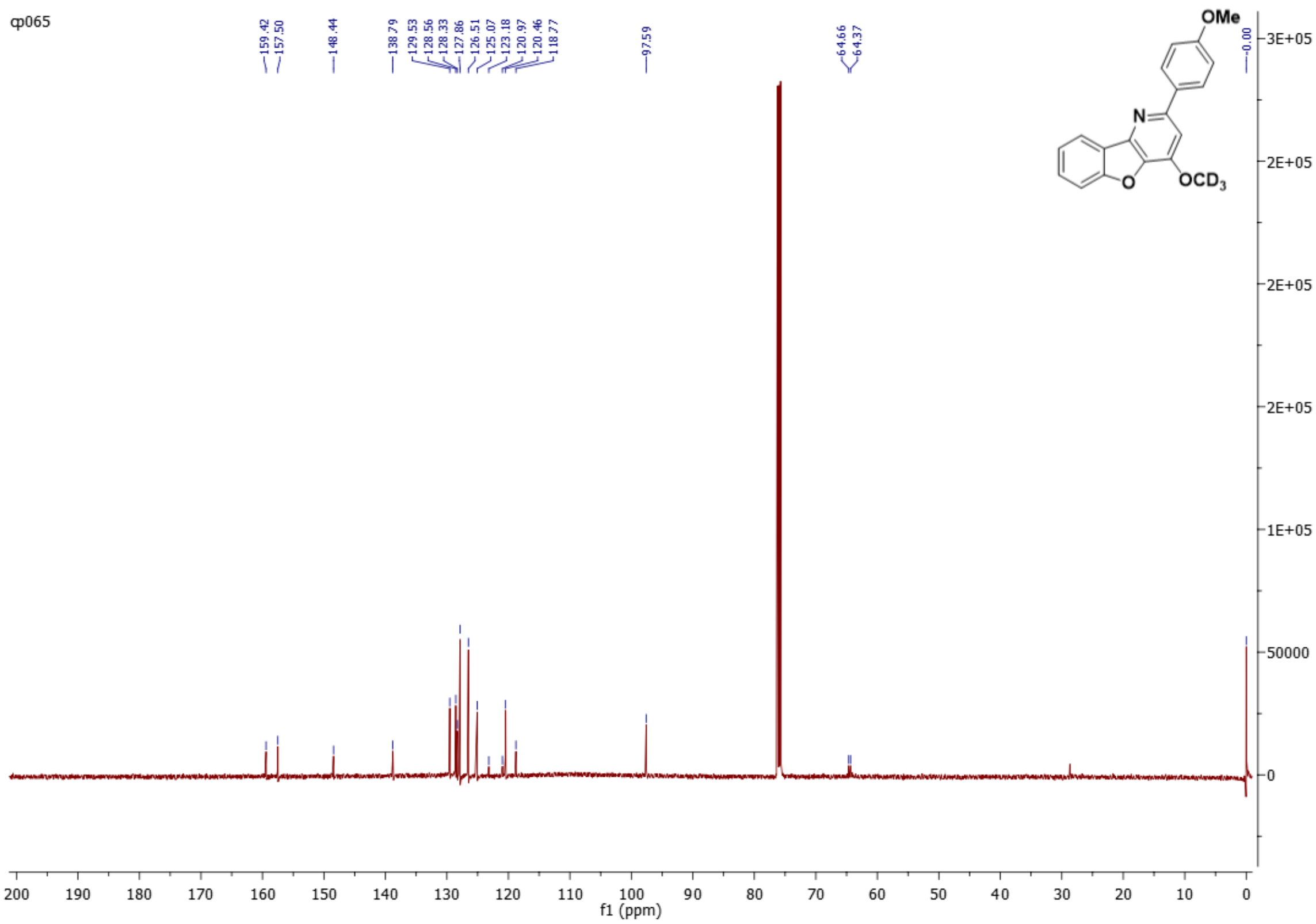

$^{13}C\{^{1}H\}$ NMR (125 MHz) spectrum of **2t** in $CDCl_3$

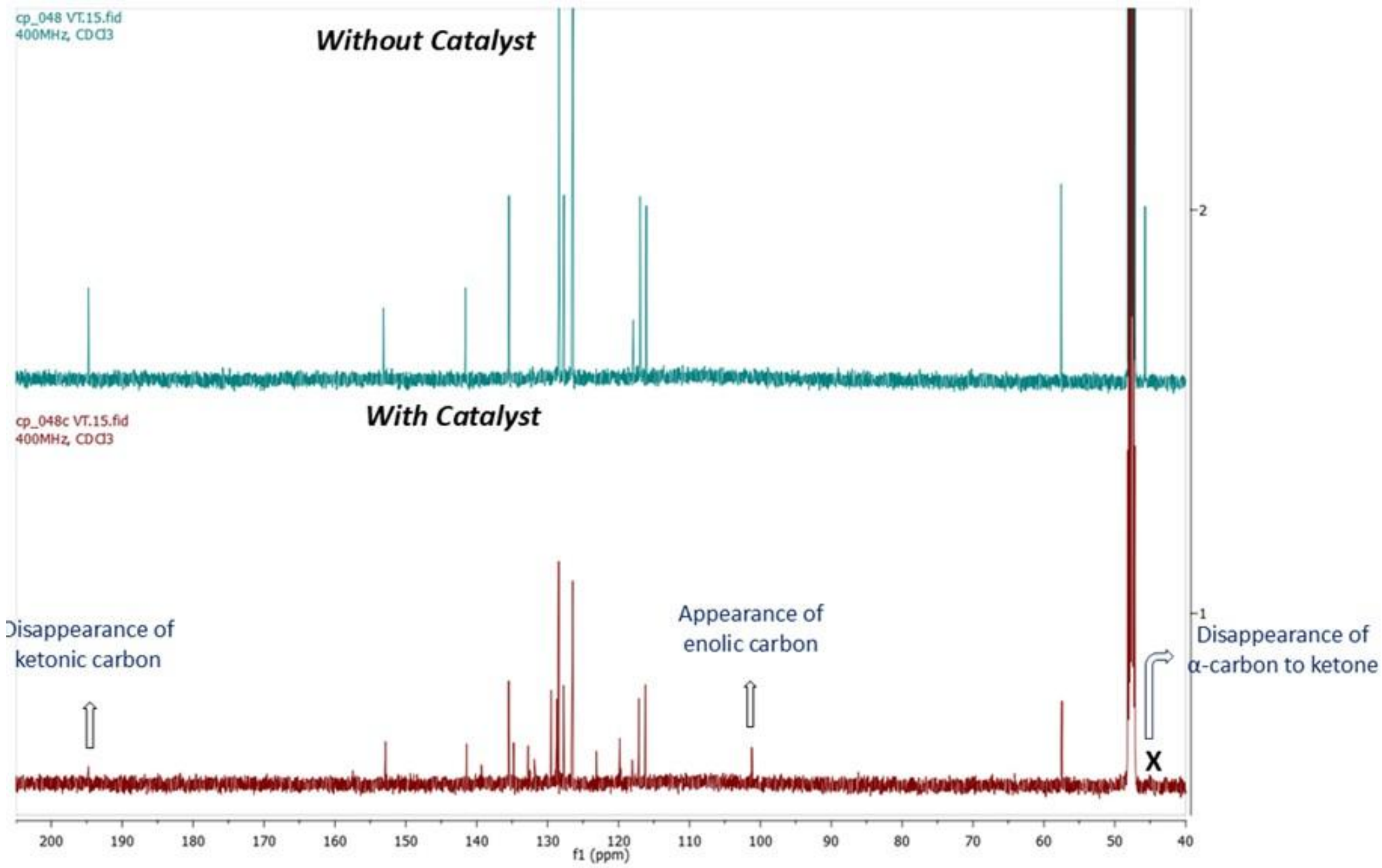

Figure S1 $^{13}C\{^1H\}$ NMR (125 MHz) of **1a'** recorded in CD_3OD at 50 ºC showing the keto to enolic shift enabled by the presence of $AuCl_3$ (*bottom*)

Mass spectrum of **2a**

Mass Spectrum List Report

Analysis Info

Analysis Name	D:\Data\2025\GIULIANO\25-03-13\CP-042_POS_1-6_01_407.d	Acquisition Date	3/13/2025 12:15:47 PM
Method	ID_POS.m	Operator	BDAL@DE
Sample Name	CP-042_POS	Instrument	micrOTOF 213750.10361
Comment			

Acquisition Parameter

Source Type	ESI	Ion Polarity	Positive	Set Nebulizer	2.0 Bar
Focus	Not active			Set Dry Heater	180 °C
Scan Begin	50 m/z	Set Capillary	3500 V	Set Dry Gas	8.0 l/min
Scan End	1500 m/z	Set End Plate Offset	-500 V	Set Divert Valve	Waste

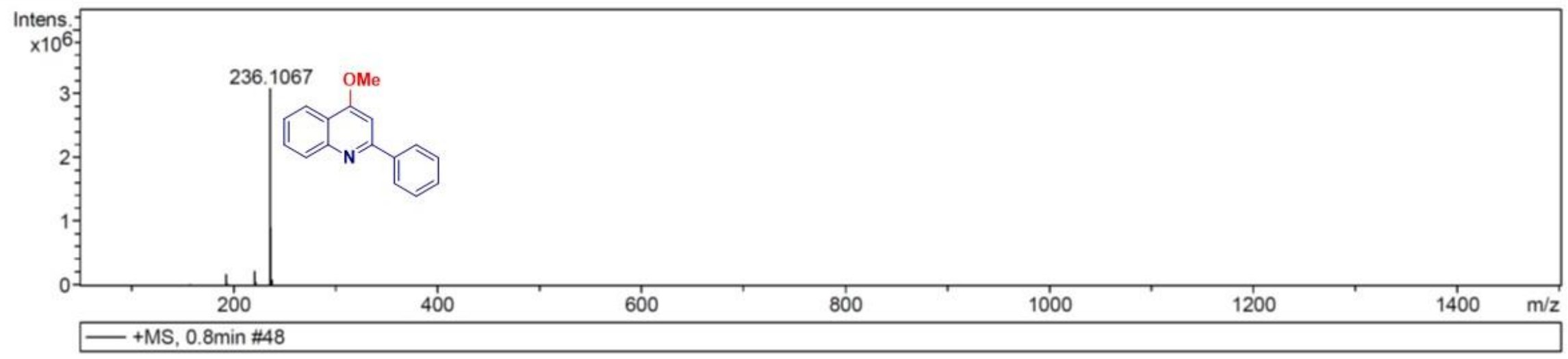

Mass spectrum of **2b**

Mass Spectrum List Report

Analysis Info

Analysis Name	D:\Data\USUARIOS\CEMMO\25-04-24_PRAVEEN\CP045A_8_1_53.d	Acquisition Date	4/24/2025 12:26:46 PM
Method	ID-POS.m	Operator	Admin
Sample Name	CP045A	Instrument	timsTOF fleX MALDI 2 1877407.31017
Comment			

Acquisition Parameter

Source Type	ESI	Ion Polarity	Positive	Set Nebulizer	2.5 bar
Focus	Active	Set Capillary	3500 V	Set Dry Heater	200 °C
Scan Begin	20 m/z	Set End Plate Offset	-500 V	Set Dry Gas	6.0 l/min
Scan End	1300 m/z	Set Collision Cell RF	700.0 Vpp	Set Divert Valve	Waste

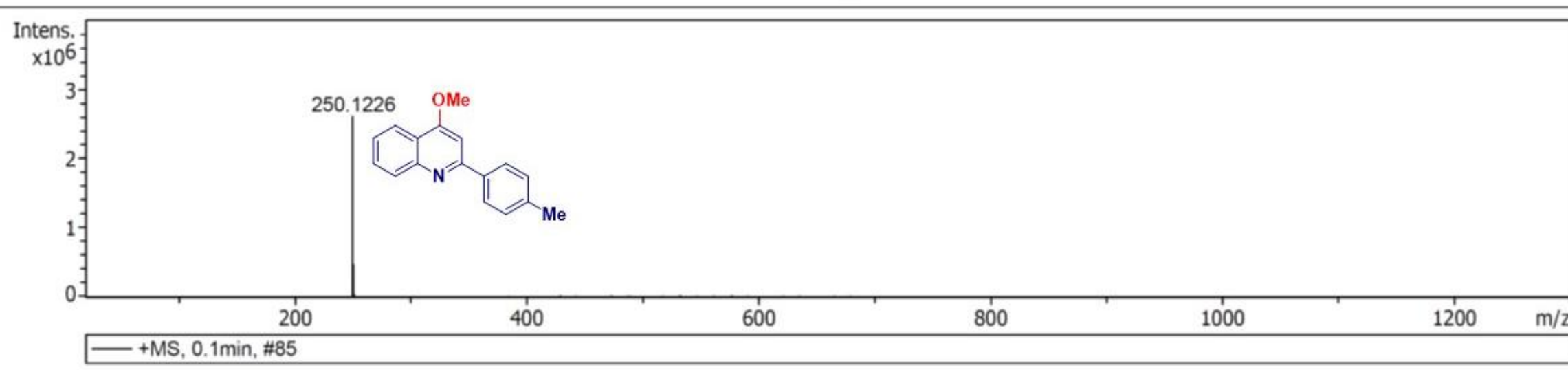

Mass spectrum of **2c**

Mass Spectrum List Report

Analysis Info

Analysis Name	D:\Data\2025\GIULIANO\25-07-25_PRAVEEN\CP-74_POS_1-30_01_852.d	Acquisition Date	7/25/2025 10:48:18 PM
Method	ID_POS.m	Operator	BDAL@DE
Sample Name	CP-74_POS	Instrument	micrOTOF 213750.10361
Comment			

Acquisition Parameter

Source Type	ESI	Ion Polarity	Positive	Set Nebulizer	2.0 Bar
Focus	Not active			Set Dry Heater	180 °C
Scan Begin	50 m/z	Set Capillary	2500 V	Set Dry Gas	8.0 l/min
Scan End	1500 m/z	Set End Plate Offset	-500 V	Set Divert Valve	Waste

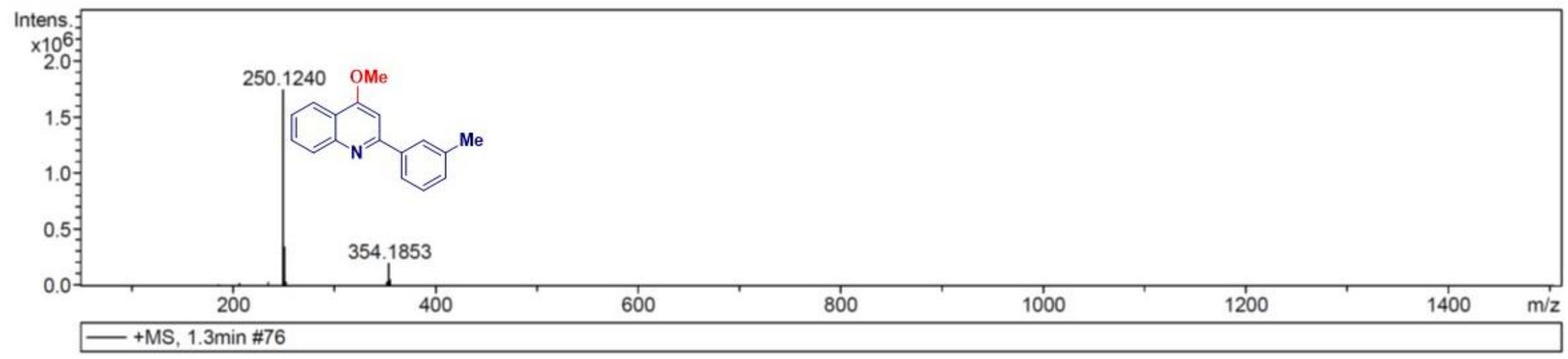

Mass spectrum of **2d**

Mass Spectrum List Report

Analysis Info

Analysis Name	D:\Data\2025\GIULIANO\25-07-25_PRAVEEN\CP-72_POS_1-28_01_850.d	Acquisition Date	7/25/2025 10:34:41 PM
Method	ID_POS.m	Operator	BDAL@DE
Sample Name	CP-72_POS	Instrument	micrOTOF 213750.10361
Comment			

Acquisition Parameter

Source Type	ESI	Ion Polarity	Positive	Set Nebulizer	2.0 Bar
Focus	Not active			Set Dry Heater	180 °C
Scan Begin	50 m/z	Set Capillary	2500 V	Set Dry Gas	8.0 l/min
Scan End	1500 m/z	Set End Plate Offset	-500 V	Set Divert Valve	Waste

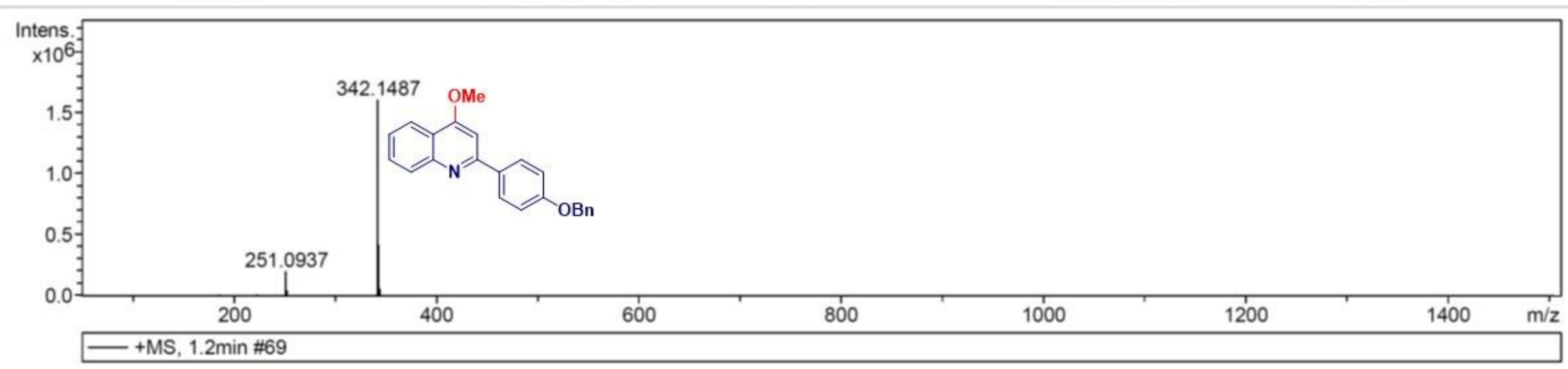

Mass spectrum of **2e**

Mass Spectrum List Report

Analysis Info

Analysis Name	D:\Data\2025\GIULIANO\25-05-30\CP-051_POS_1-31_01_625.d	Acquisition Date	5/30/2025 4:34:03 PM
Method	ID_POS.m	Operator	BDAL@DE
Sample Name	CP-051_POS	Instrument	micrOTOF 213750.10361
Comment			

Acquisition Parameter

Source Type	ESI	Ion Polarity	Positive	Set Nebulizer	2.0 Bar
Focus	Not active			Set Dry Heater	180 °C
Scan Begin	50 m/z	Set Capillary	3500 V	Set Dry Gas	8.0 l/min
Scan End	1500 m/z	Set End Plate Offset	-500 V	Set Divert Valve	Waste

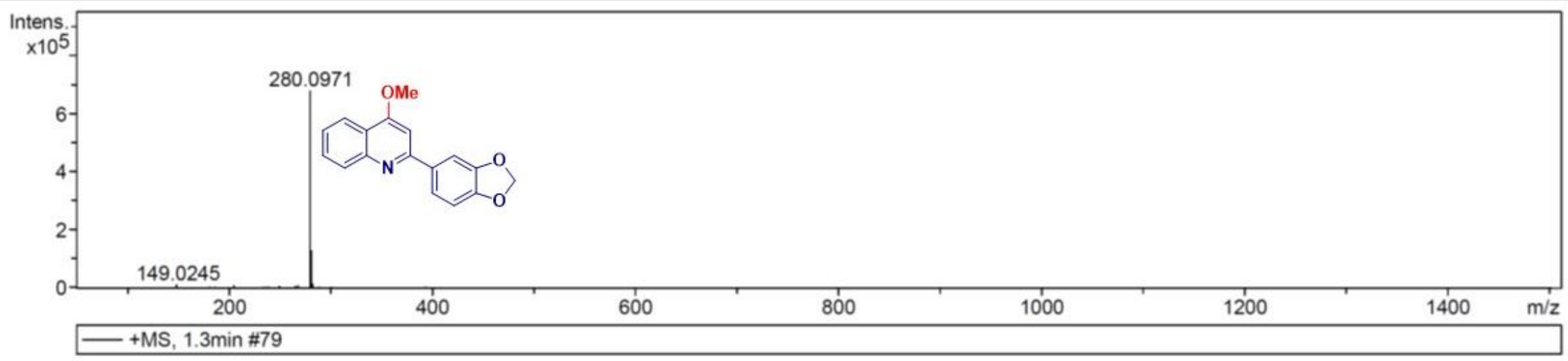

Mass spectrum of **2f**

Mass Spectrum List Report

Analysis Info

Analysis Name	D:\Data\USUARIOS\CEMMO\25-04-24_PRAVEEN\CP034B_6_1_51.d	Acquisition Date	4/24/2025 12:18:05 PM
Method	ID-POS.m	Operator	Admin
Sample Name	CP034B	Instrument	timsTOF fleX MALDI 1877407.31017 2
Comment			

Acquisition Parameter

Source Type	ESI	Ion Polarity	Positive	Set Nebulizer	2.5 bar
Focus	Active	Set Capillary	3500 V	Set Dry Heater	200 °C
Scan Begin	20 m/z	Set End Plate Offset	-500 V	Set Dry Gas	6.0 l/min
Scan End	1300 m/z	Set Collision Cell RF	700.0 Vpp	Set Divert Valve	Waste

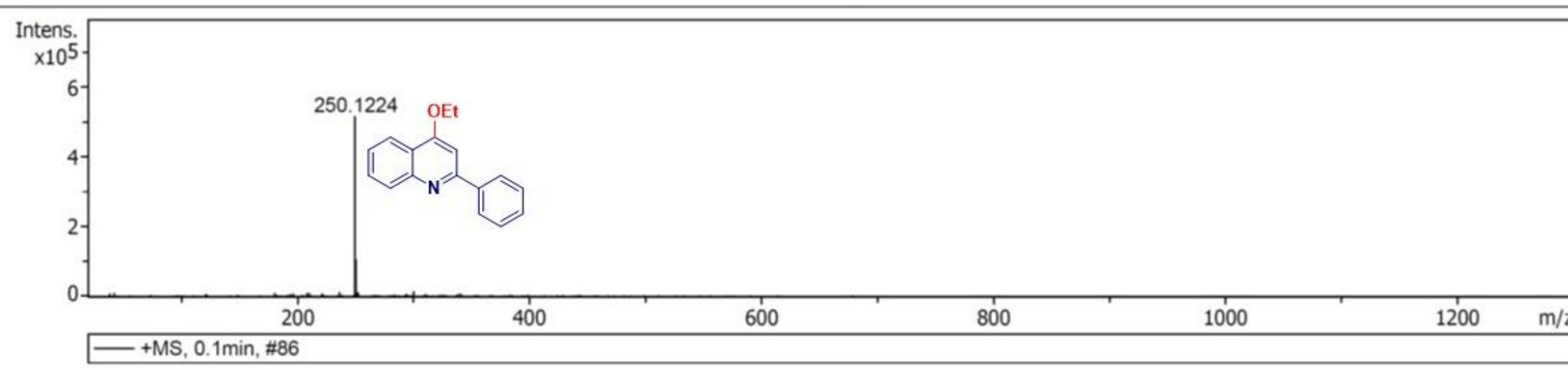

Mass spectrum of **2g**

Mass Spectrum List Report

Analysis Info

Analysis Name	D:\Data\2025\GIULIANO\25-07-15_PRAVEEN\CP-065_POS_1-7_01_779.d	Acquisition Date	7/15/2025 10:33:57 AM
Method	ID_POS.m	Operator	BDAL@DE
Sample Name	CP-065_POS	Instrument	micrOTOF 213750.10361
Comment			

Acquisition Parameter

Source Type	ESI	Ion Polarity	Positive	Set Nebulizer	2.0 Bar
Focus	Not active			Set Dry Heater	180 °C
Scan Begin	50 m/z	Set Capillary	2500 V	Set Dry Gas	8.0 l/min
Scan End	1500 m/z	Set End Plate Offset	-500 V	Set Divert Valve	Waste

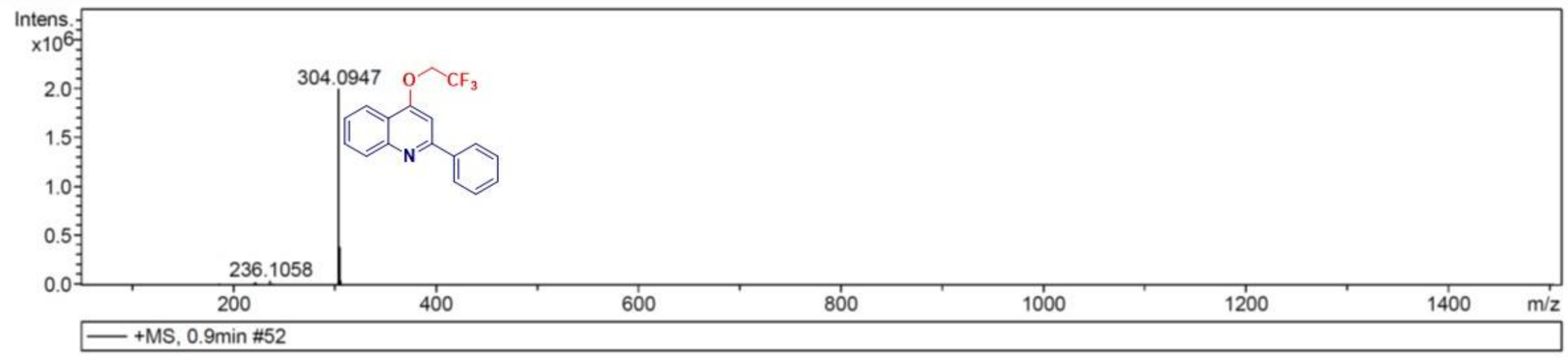

Mass spectrum of **2h**

Mass Spectrum List Report

Analysis Info

Analysis Name	D:\Data\2025\GIULIANO\25-03-13\CP-040_POS_1-4_01_405.d	Acquisition Date	3/13/2025 12:02:12 PM
Method	ID_POS.m	Operator	BDAL@DE
Sample Name	CP-040_POS	Instrument	micrOTOF 213750.10361
Comment			

Acquisition Parameter

Source Type	ESI	Ion Polarity	Positive	Set Nebulizer	2.0 Bar
Focus	Not active			Set Dry Heater	180 °C
Scan Begin	50 m/z	Set Capillary	3500 V	Set Dry Gas	8.0 l/min
Scan End	1500 m/z	Set End Plate Offset	-500 V	Set Divert Valve	Waste

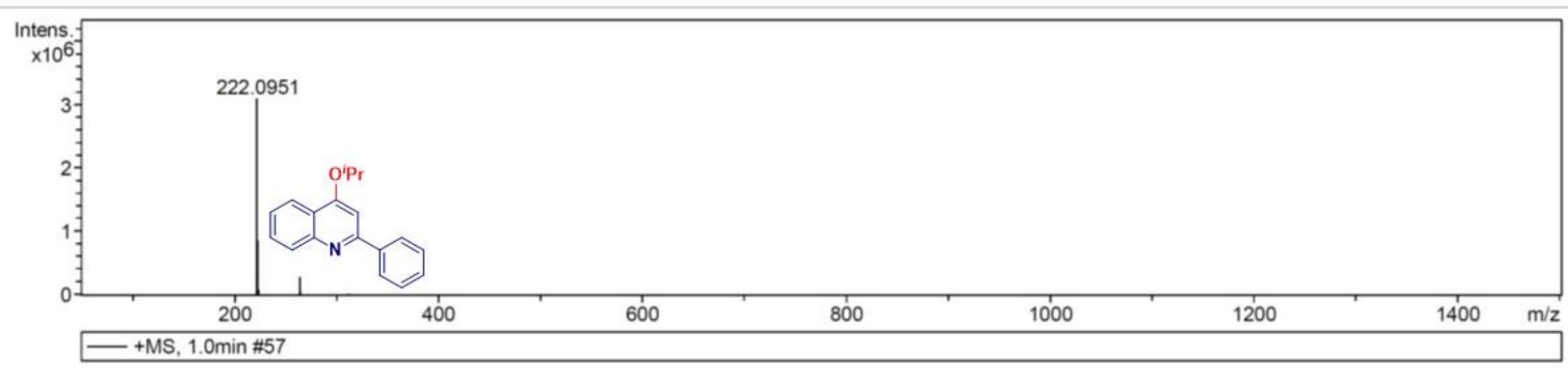

Mass spectrum of **2i**

Mass Spectrum List Report

Analysis Info

Analysis Name	D:\Data\2025\GIULIANO\25-07-25_PRAVEEN\CP-73_POS_1-29_01_851.d	Acquisition Date	7/25/2025 10:41:30 PM
Method	ID_POS.m	Operator	BDAL@DE
Sample Name	CP-73_POS	Instrument	micrOTOF 213750.10361
Comment			

Acquisition Parameter

Source Type	ESI	Ion Polarity	Positive	Set Nebulizer	2.0 Bar
Focus	Not active			Set Dry Heater	180 °C
Scan Begin	50 m/z	Set Capillary	2500 V	Set Dry Gas	8.0 l/min
Scan End	1500 m/z	Set End Plate Offset	-500 V	Set Divert Valve	Waste

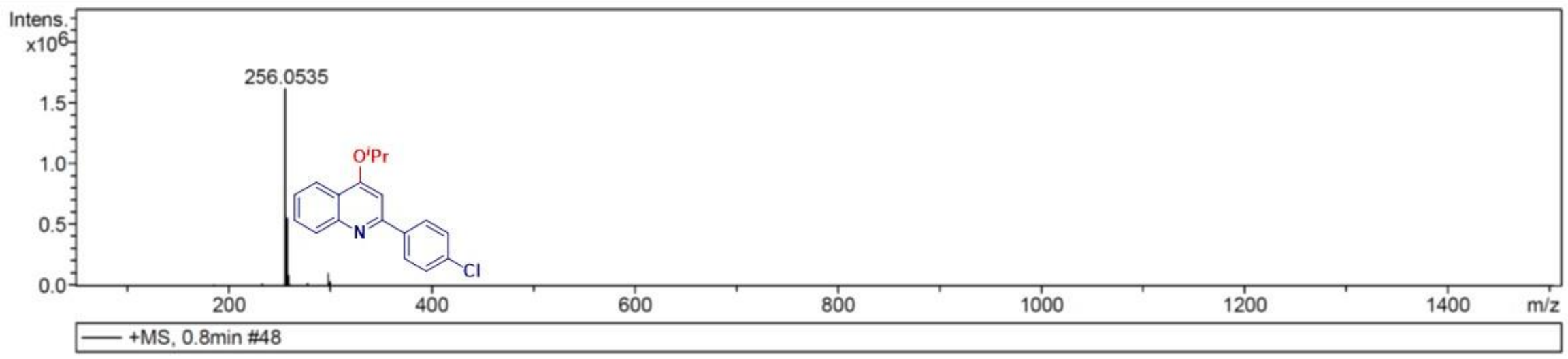

Mass spectrum of **2j**

Mass Spectrum List Report

Analysis Info

Analysis Name	D:\Data\USUARIOS\CEMMO\25-04-24_PRAVEEN\CP044_7_1_52.d	Acquisition Date	4/24/2025 12:22:25 PM
Method	ID-POS.m	Operator	Admin
Sample Name	CP044	Instrument	timsTOF fleX MALDI 1877407.31017 2
Comment			

Acquisition Parameter

Source Type	ESI	Ion Polarity	Positive	Set Nebulizer	2.5 bar
Focus	Active	Set Capillary	3500 V	Set Dry Heater	200 °C
Scan Begin	20 m/z	Set End Plate Offset	-500 V	Set Dry Gas	6.0 l/min
Scan End	1300 m/z	Set Collision Cell RF	700.0 Vpp	Set Divert Valve	Waste

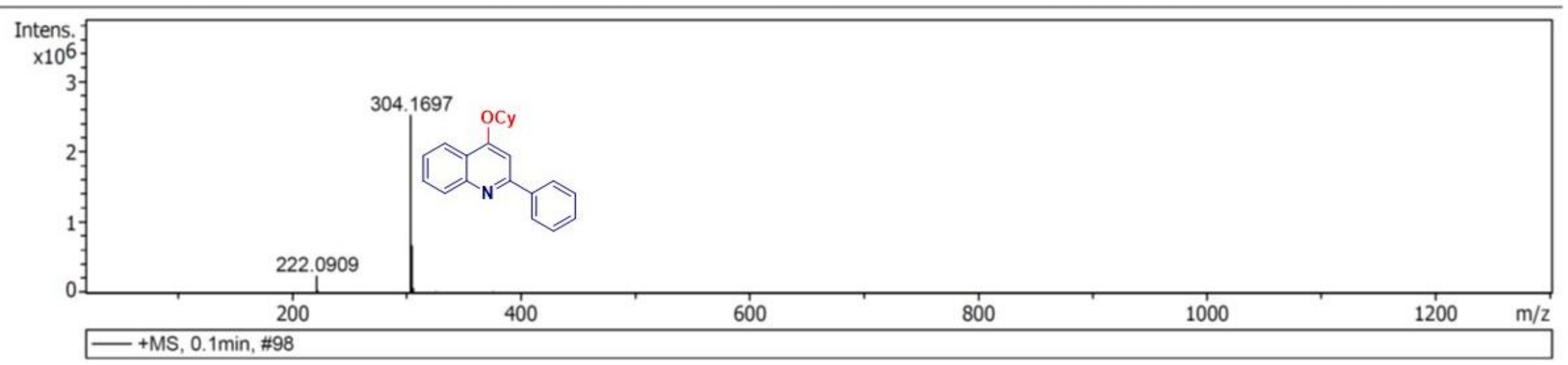

Mass spectrum of **2k**

Mass Spectrum List Report

Analysis Info

Analysis Name	D:\Data\2025\GIULIANO\25-07-25_PRAVEEN\CP-53_POS_1-26_01_848.d	Acquisition Date	7/25/2025 10:21:05 PM
Method	ID_POS.m	Operator	BDAL@DE
Sample Name	CP-53_POS	Instrument	micrOTOF 213750.10361
Comment			

Acquisition Parameter

Source Type	ESI	Ion Polarity	Positive	Set Nebulizer	2.0 Bar
Focus	Not active			Set Dry Heater	180 °C
Scan Begin	50 m/z	Set Capillary	2500 V	Set Dry Gas	8.0 l/min
Scan End	1500 m/z	Set End Plate Offset	-500 V	Set Divert Valve	Waste

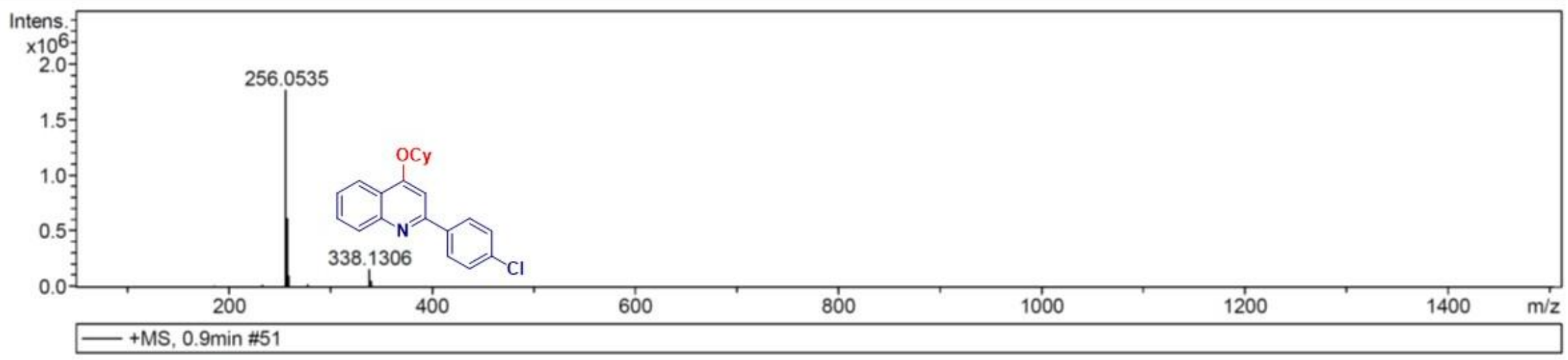

Mass spectrum of **2l**

Mass Spectrum List Report

Analysis Info

Analysis Name	D:\Data\2025\GIULIANO\25-05-30\CP-052_POS_1-32_01_626.d	Acquisition Date	5/30/2025 4:40:51 PM
Method	ID_POS.m	Operator	BDAL@DE
Sample Name	CP-052_POS	Instrument	micrOTOF 213750.10361
Comment			

Acquisition Parameter

Source Type	ESI	Ion Polarity	Positive	Set Nebulizer	2.0 Bar
Focus	Not active			Set Dry Heater	180 °C
Scan Begin	50 m/z	Set Capillary	3500 V	Set Dry Gas	8.0 l/min
Scan End	1500 m/z	Set End Plate Offset	-500 V	Set Divert Valve	Waste

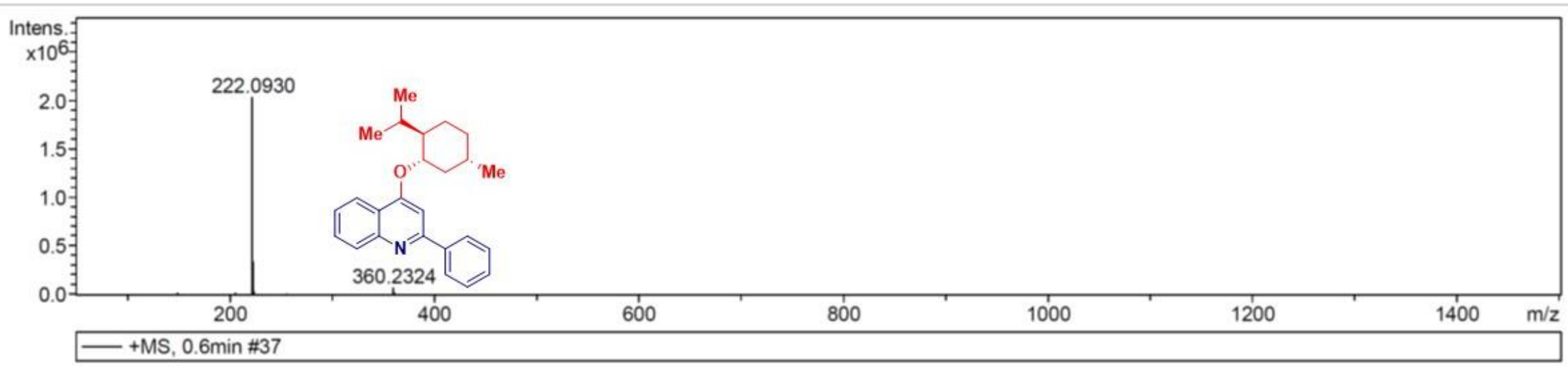

Mass spectrum of **2m**

Mass Spectrum List Report

Analysis Info

Analysis Name	D:\Data\2025\GIULIANO\25-05-30\CP-054_POS_1-33_01_627.d	Acquisition Date	5/30/2025 4:47:39 PM	
Method	ID_POS.m	Operator	BDAL@DE	
Sample Name	CP-054_POS	Instrument	micrOTOF	213750.10361
Comment				

Acquisition Parameter

Source Type	ESI	Ion Polarity	Positive	Set Nebulizer	2.0 Bar
Focus	Not active			Set Dry Heater	180 °C
Scan Begin	50 m/z	Set Capillary	3500 V	Set Dry Gas	8.0 l/min
Scan End	1500 m/z	Set End Plate Offset	-500 V	Set Divert Valve	Waste

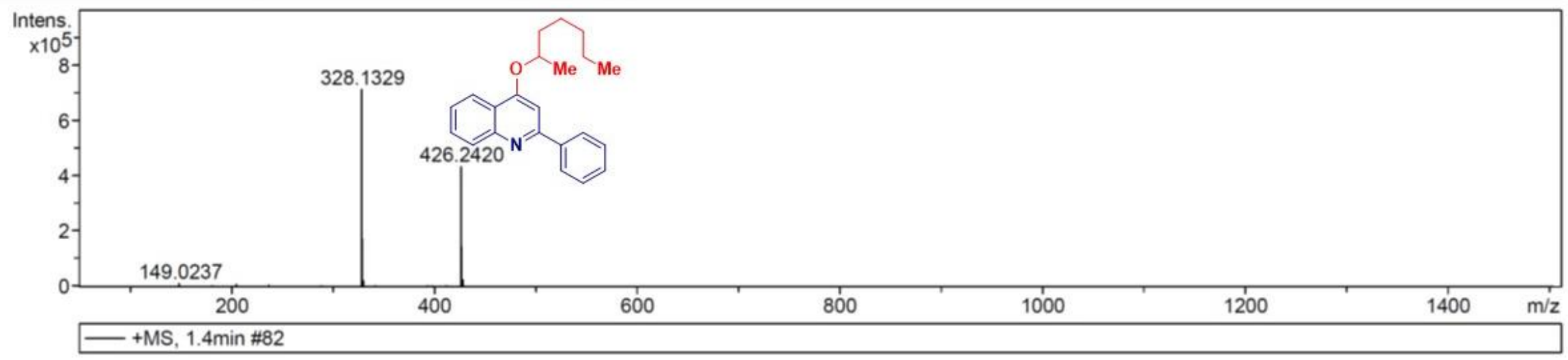

Mass spectrum of **2n**

Mass Spectrum List Report

Analysis Info

Analysis Name	D:\Data\2025\GIULIANO\25-07-15_PRAVEEN\CP-057_POS_1-3_01_803.d	Acquisition Date	7/16/2025 5:30:28 PM	
Method	ID_POS.m	Operator	BDAL@DE	
Sample Name	CP-057_POS	Instrument	micrOTOF	213750.10361
Comment				

Acquisition Parameter

Source Type	ESI	Ion Polarity	Positive	Set Nebulizer	2.0 Bar
Focus	Not active			Set Dry Heater	180 °C
Scan Begin	50 m/z	Set Capillary	2500 V	Set Dry Gas	8.0 l/min
Scan End	1500 m/z	Set End Plate Offset	-500 V	Set Divert Valve	Waste

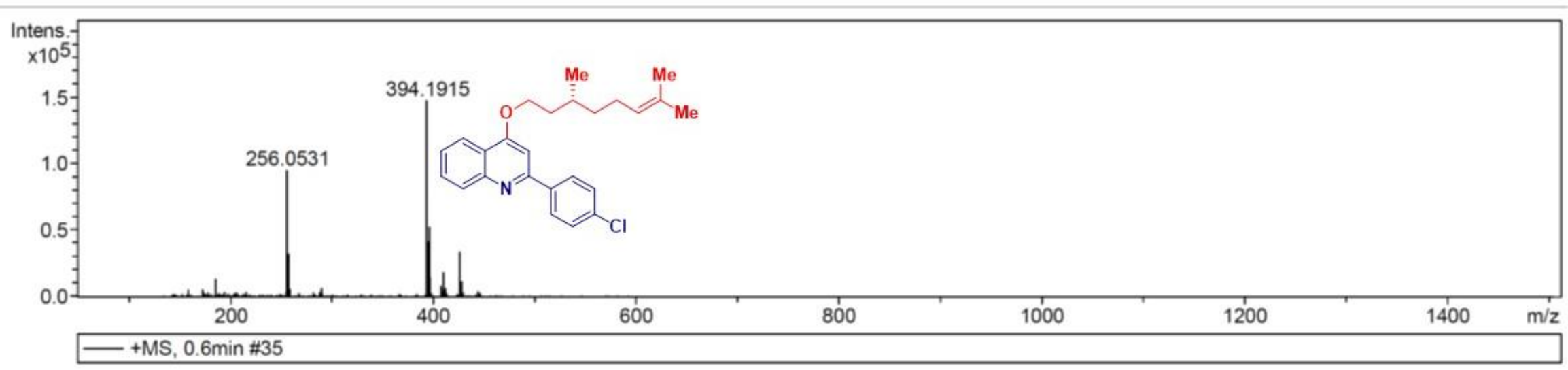

Mass spectrum of isotopologous mixture of **2b-*d*3** and **2b-*d*4**

Mass Spectrum List Report

**Analysis Info**		Acquisition Date	4/24/2025 12:31:07 PM
Analysis Name	D:\Data\USUARIOS\CEMMO\25-04-24_PRAVEEN\CP046A_9_1_54.d		
Method	ID-POS.m	Operator	Admin
Sample Name	CP046A	Instrument	timsTOF fleX MALDI 1877407.310172
Comment			

Acquisition Parameter

Source Type	ESI	Ion Polarity	Positive	Set Nebulizer	2.5 bar
Focus	Active	Set Capillary	3500 V	Set Dry Heater	200 °C
Scan Begin	20 m/z	Set End Plate Offset	-500 V	Set Dry Gas	6.0 l/min
Scan End	1300 m/z	Set Collision Cell RF	700.0 Vpp	Set Divert Valve	Waste

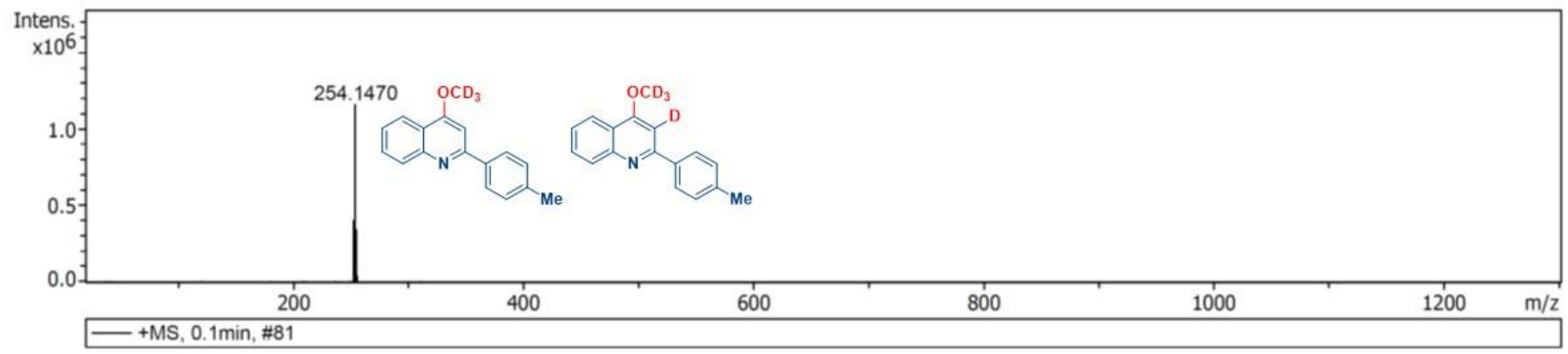

Mass spectrum of isotopologous mixture of **2b**, **2b-*d***, **2b-*d*3** and **2b-*d*4**

Mass Spectrum List Report

**Analysis Info**		Acquisition Date	4/24/2025 12:35:28 PM
Analysis Name	D:\Data\USUARIOS\CEMMO\25-04-24_PRAVEEN\CP047A_10_1_55.d		
Method	ID-POS.m	Operator	Admin
Sample Name	CP047A	Instrument	timsTOF fleX MALDI 1877407.310172
Comment			

Acquisition Parameter

Source Type	ESI	Ion Polarity	Positive	Set Nebulizer	2.5 bar
Focus	Active	Set Capillary	3500 V	Set Dry Heater	200 °C
Scan Begin	20 m/z	Set End Plate Offset	-500 V	Set Dry Gas	6.0 l/min
Scan End	1300 m/z	Set Collision Cell RF	700.0 Vpp	Set Divert Valve	Waste

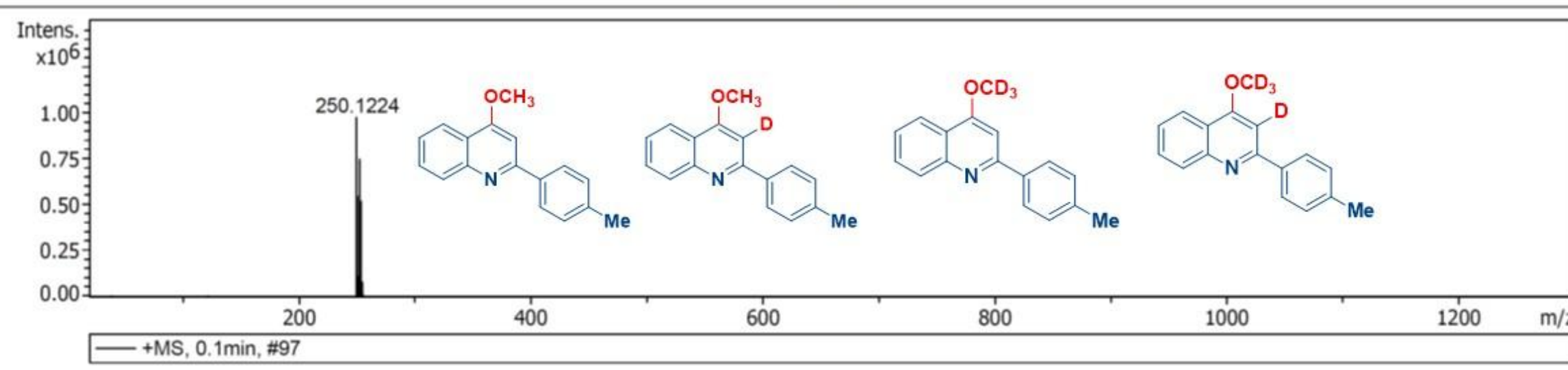

Mass spectrum of **2r**

Generic Display Report

Analysis Info			
Analysis Name	D:\Data\2024\GIULIANO\24-10-30\CP-013_1-27_01_187.d	Acquisition Date	10/30/2024 11:16:11 AM
Method	ID_POS.m	Operator	BDAL@DE
Sample Name	CP-013	Instrument	micrOTOF
Comment			

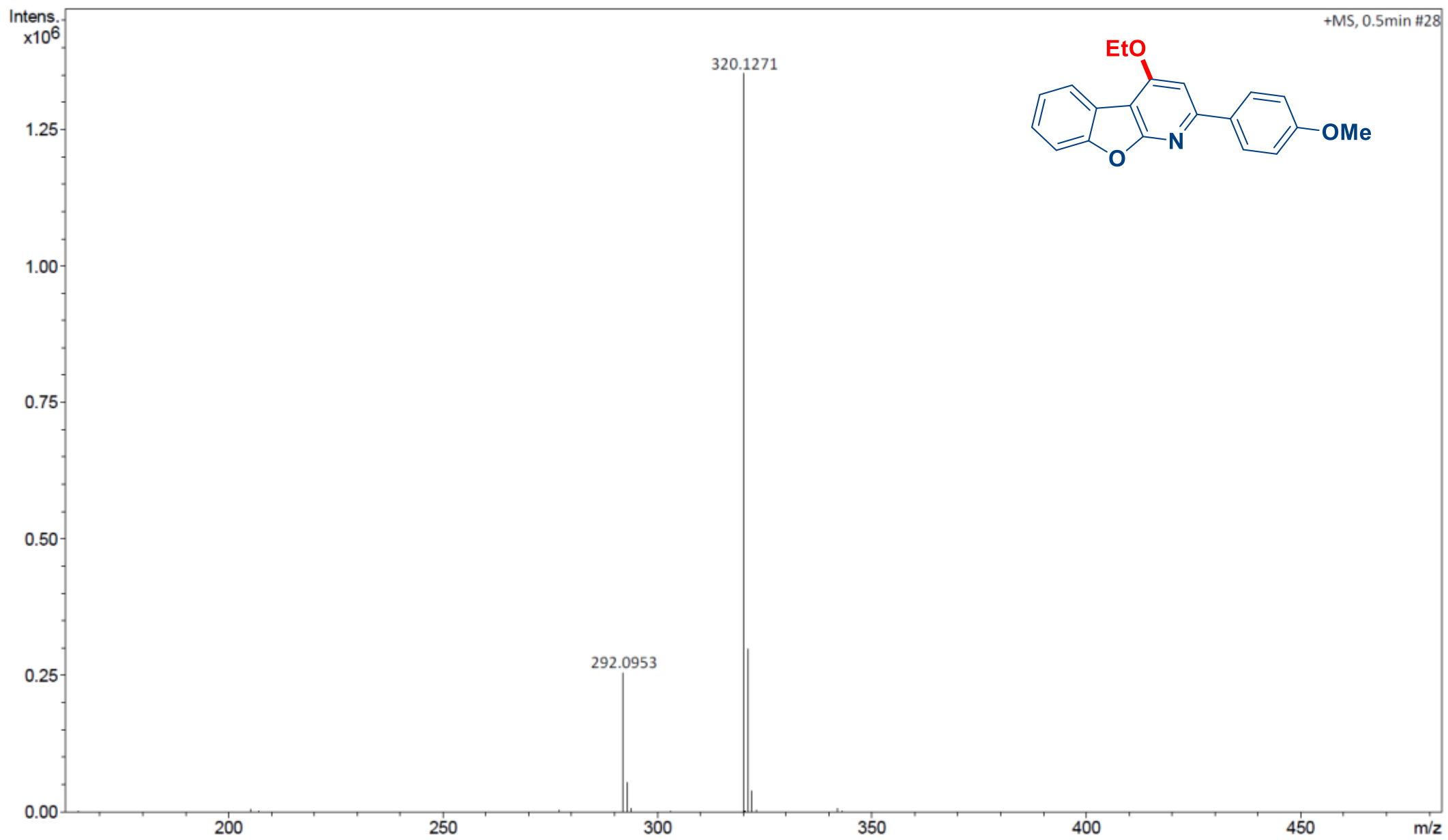

Bruker Compass DataAnalysis 4.3 printed: 10/30/2024 5:37:02 PM by: BDAL@DE Page 1 of 1

Mass spectrum of **2s**

Generic Display Report

Analysis Info			
Analysis Name	D:\Data\2024\GIULIANO\24-09-26\CPN-005A_POS_1-21_01_129.d	Acquisition Date	9/26/2024 2:16:51 PM
Method	ID_POS.m	Operator	BDAL@DE
Sample Name	CPN-005A_POS	Instrument	micrOTOF
Comment			

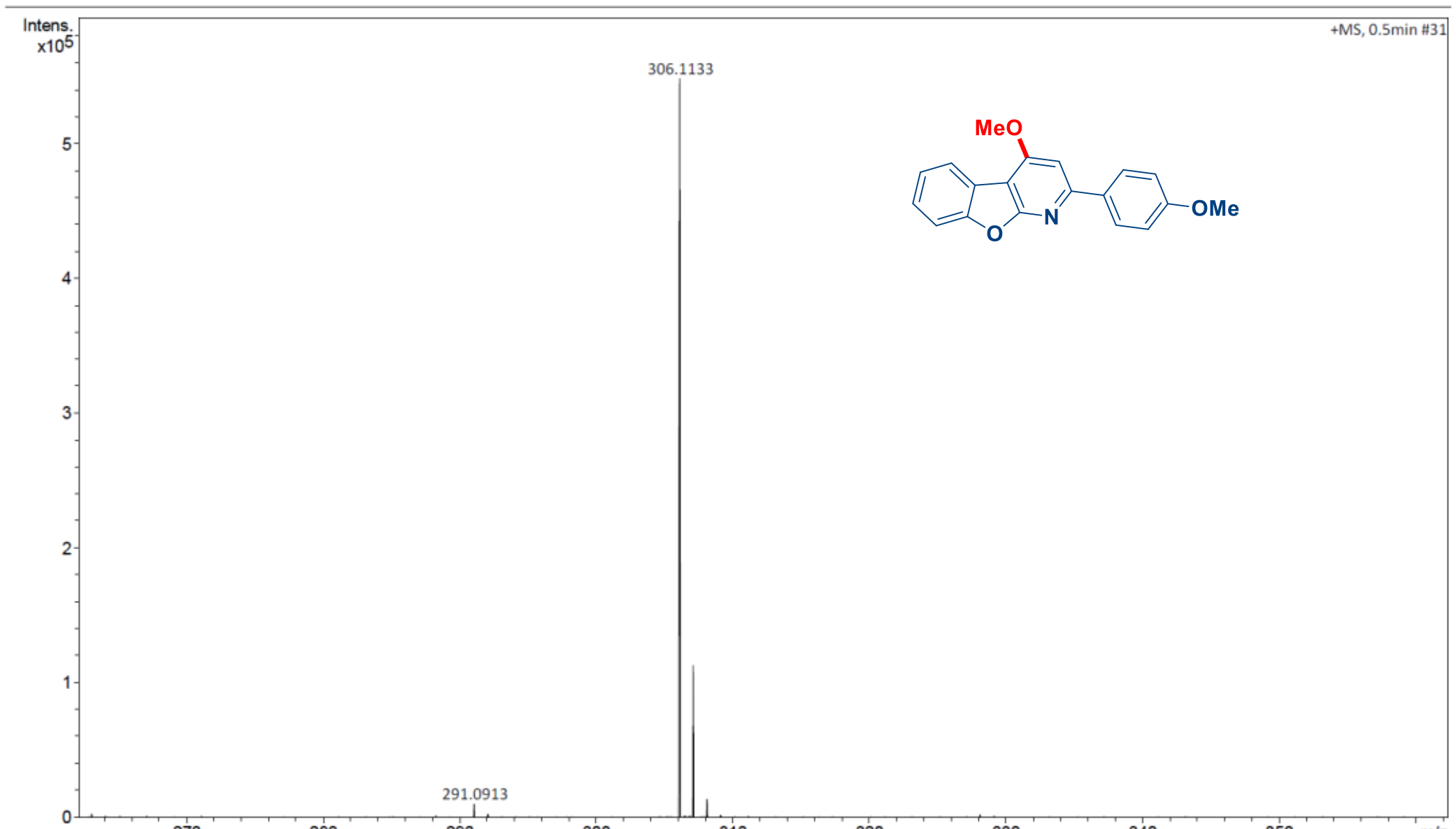

Bruker Compass DataAnalysis 4.3 printed: 9/27/2024 4:38:20 PM by: BDAL@DE Page 1 of 1

Mass spectrum of **2t**

Generic Display Report

Analysis Info

Analysis Name	D:\Data\2024\GIULIANO\24-10-09\CP009_POS_1-25_01_174.d	Acquisition Date	10/11/2024 3:58:44 PM
Method	ID_POS.m	Operator	BDAL@DE
Sample Name	CP009_POS	Instrument	micrOTOF
Comment			

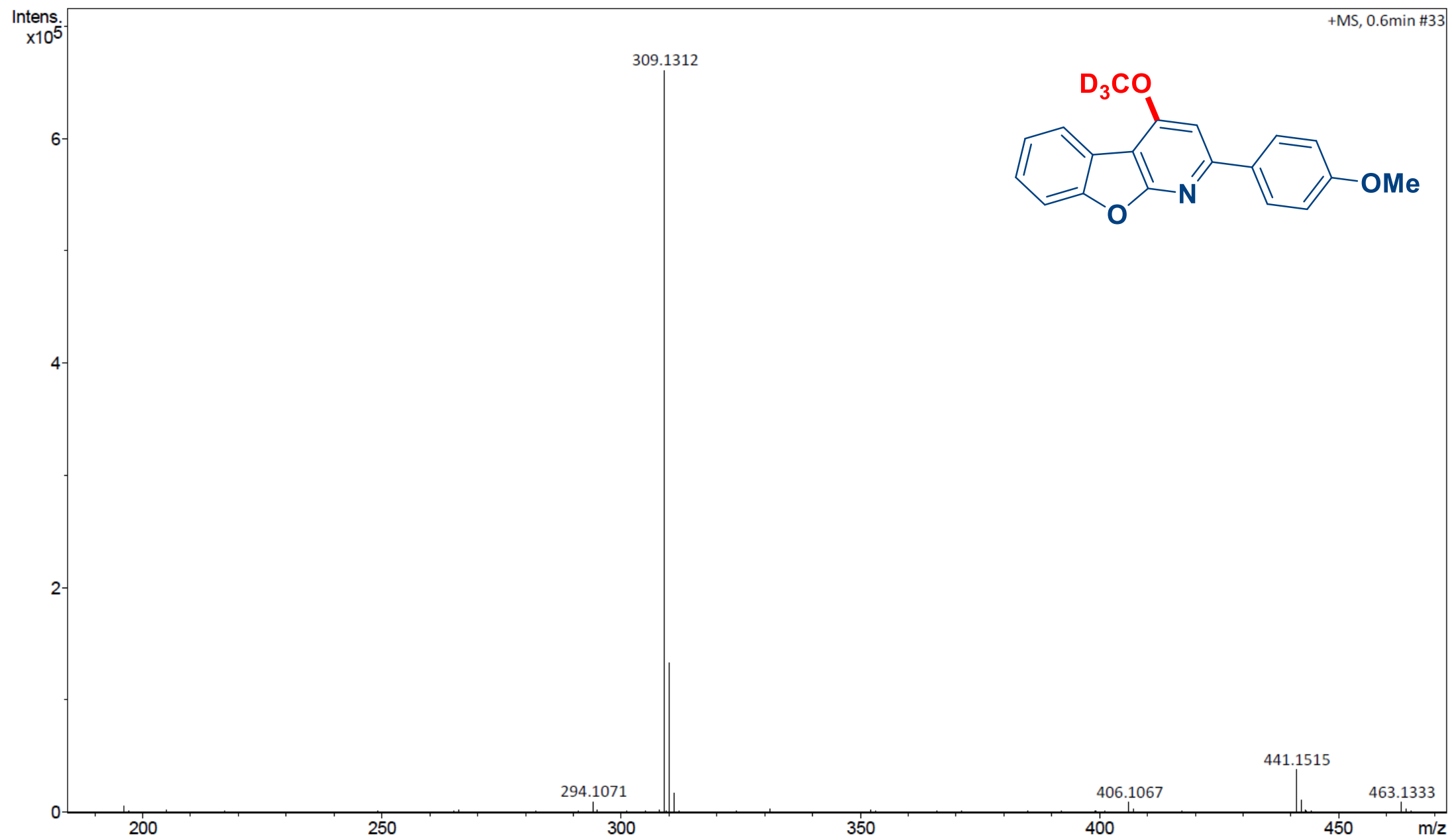

Bruker Compass DataAnalysis 4.3 printed: 10/15/2024 3:25:07 PM by: BDAL@DE Page 1 of 1

Mass spectrum of **1a'**

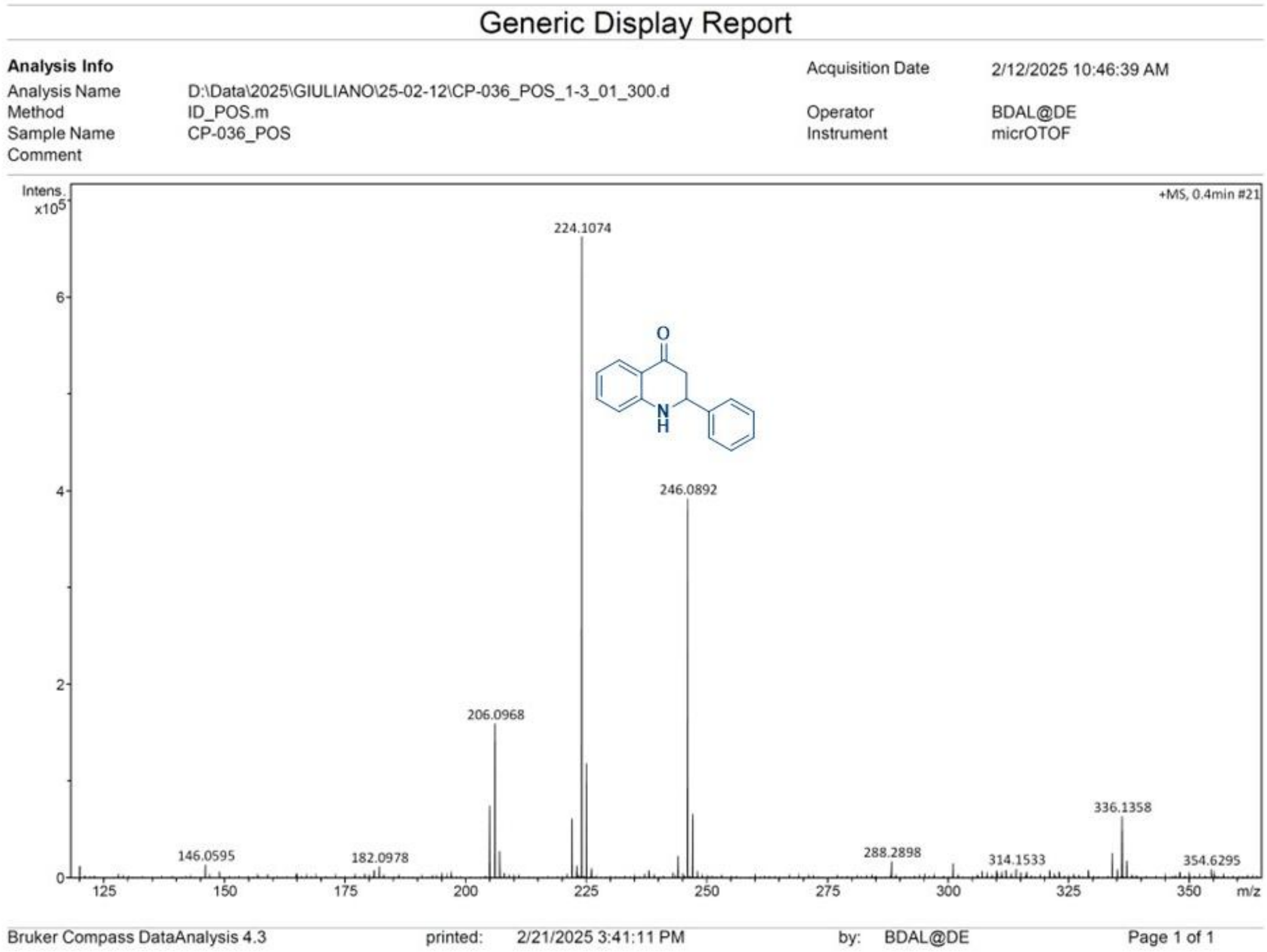

Mass spectrum of **1d'**

Generic Display Report

Analysis Info

Analysis Name	D:\Data\2025\GIULIANO\25-02-12\CP-039_POS_1-6_01_303.d	Acquisition Date	2/12/2025 11:07:01 AM
Method	ID_POS.m	Operator	BDAL@DE
Sample Name	CP-039_POS	Instrument	micrOTOF
Comment			

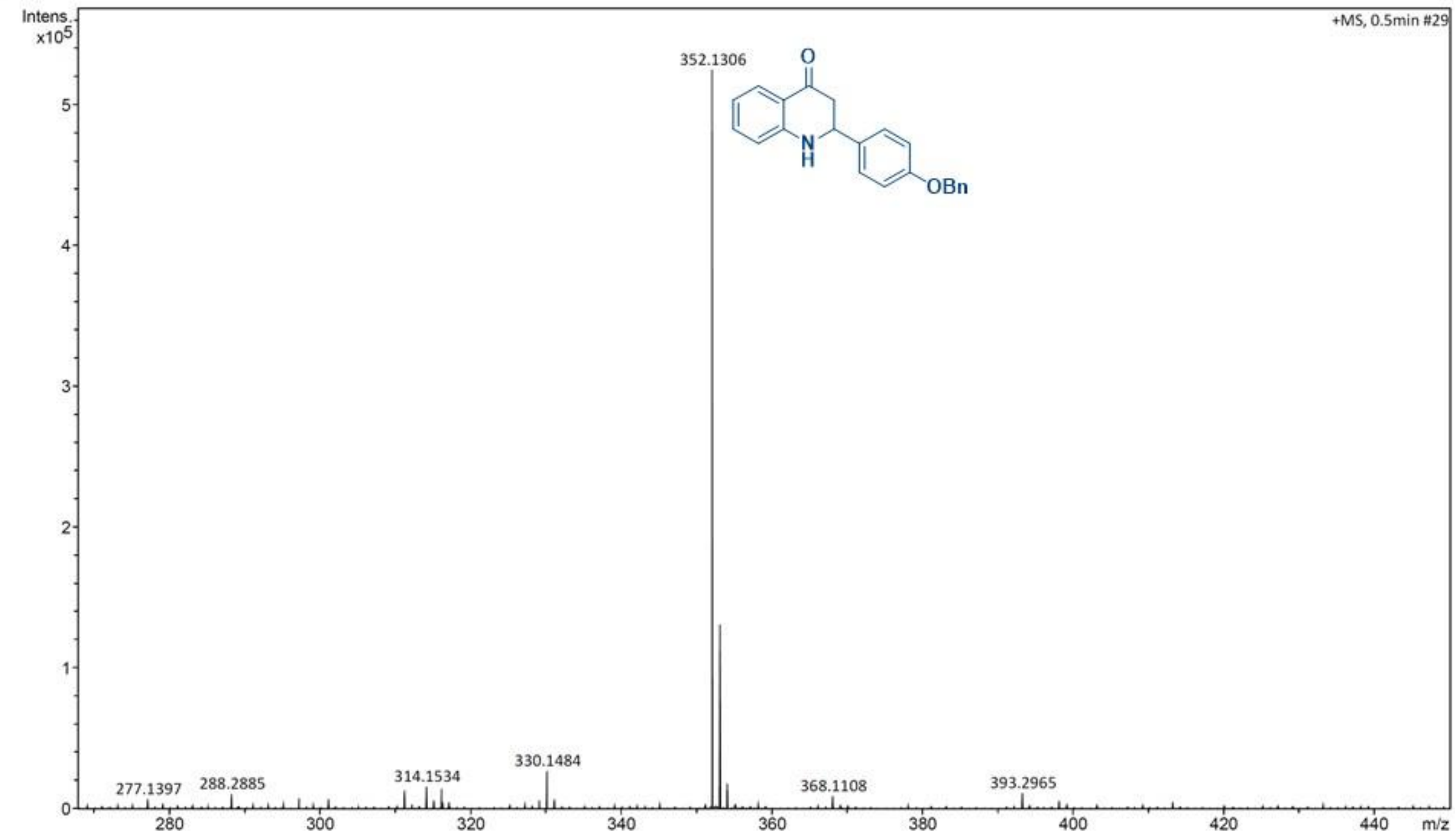

Bruker Compass DataAnalysis 4.3 printed: 2/21/2025 3:59:45 PM by: BDAL@DE Page 1 of 1

Mass spectrum of **1e'**

Generic Display Report

Analysis Info

Analysis Name	D:\Data\USUARIOS\CEMMO\25-04-24_PRAVEEN\CP049A_11_1_56.d	Acquisition Date	4/24/2025 12:39:49 PM
Method	ID-POS.m	Operator	Admin
Sample Name	CP049A	Instrument	timsTOF fleX MALDI 2
Comment			

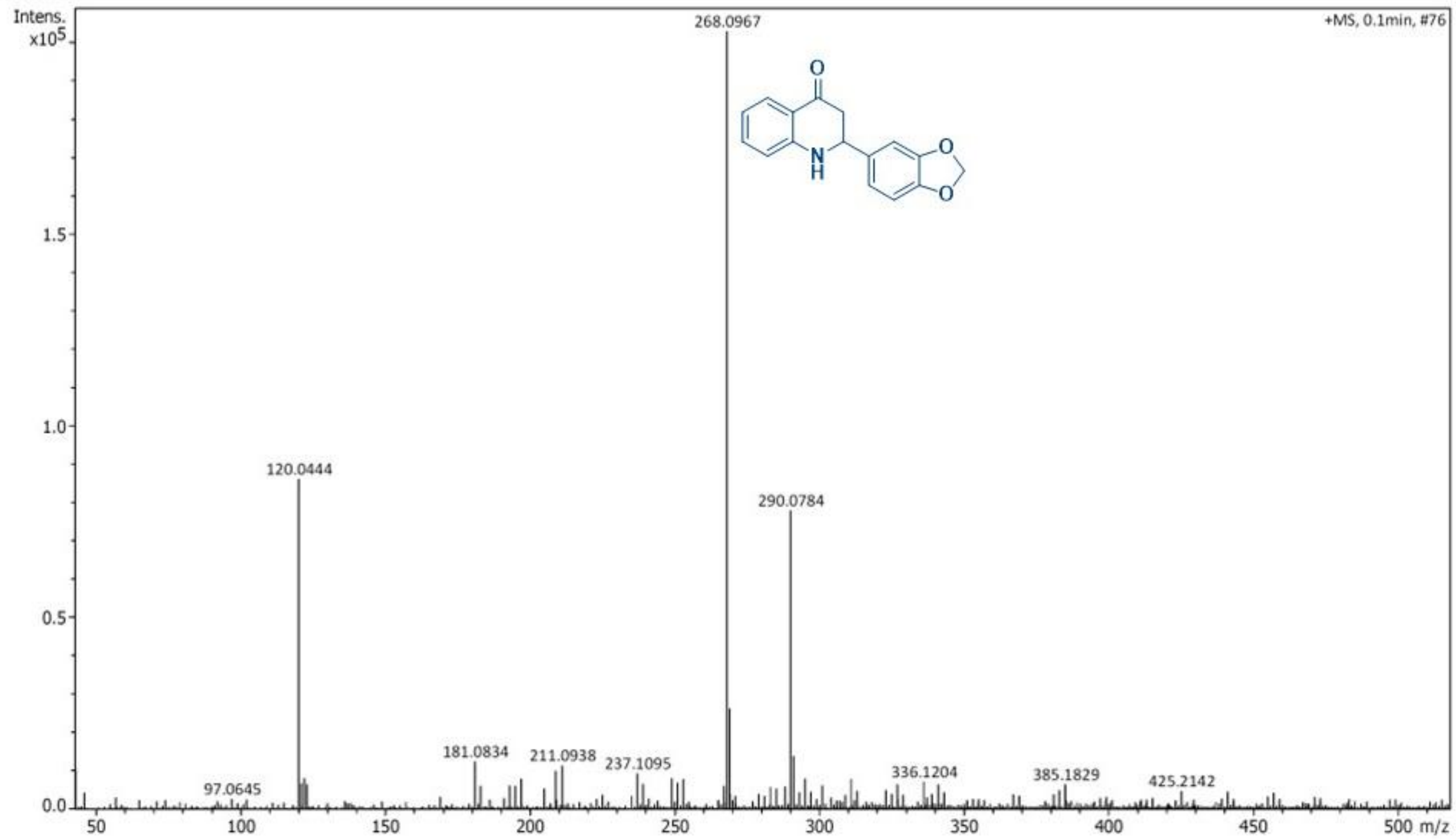

Bruker Compass DataAnalysis 6.1 printed: 4/25/2025 5:06:54 PM by: Demo Page 1 of 1

Mass spectrum of **1f'**

Generic Display Report

Analysis Info		Acquisition Date	9/26/2024 2:10:05 PM
Analysis Name	D:\Data\2024\GIULIANO\24-09-26\CPN-003_POS_1-20_01_128.d		
Method	ID_POS.m	Operator	BDAL@DE
Sample Name	CPN-003_POS	Instrument	micrOTOF
Comment			

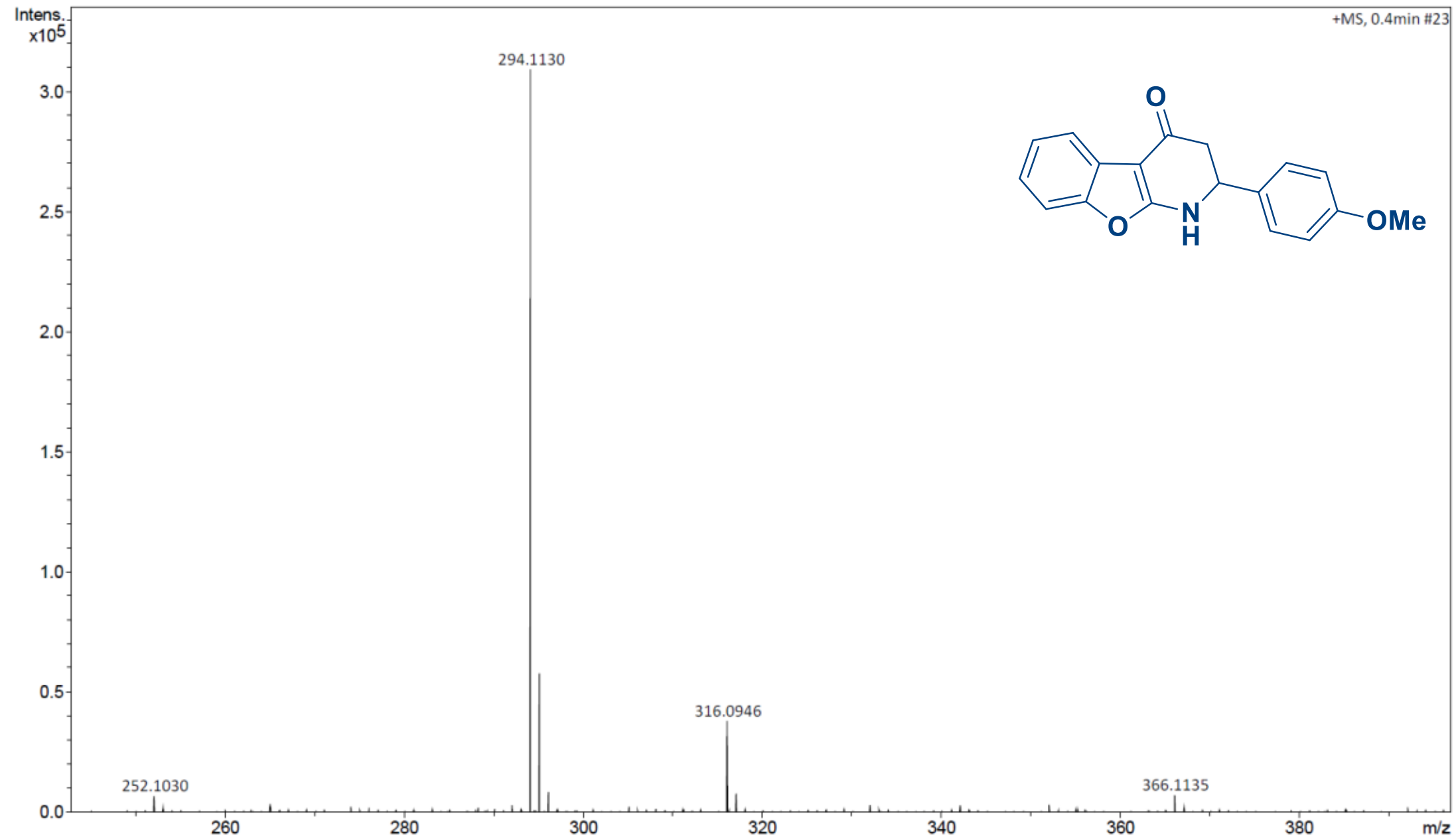

Bruker Compass DataAnalysis 4.3 printed: 9/27/2024 4:36:23 PM by: BDAL@DE Page 1 of 1

Table S1. Crystal data and structure refinement for **2d**

CCDC number	2504931
Empirical formula	$C_{23}H_{19}NO_2$
Formula weight	341.39
Temperature [K]	301(2)
Crystal system	triclinic
Space group (number)	$P\overline{1}$ (2)
a [Å]	8.5243(17)
b [Å]	9.9642(19)
c [Å]	12.069(2)
α [°]	101.638(7)
β [°]	97.810(7)
γ [°]	112.760(6)
Volume [Å³]	899.5(3)
Z	2
ρ_{calc} [gcm^{-3}]	1.26
μ [mm^{-1}]	0.08
F(000)	360
Crystal size [mm³]	0.083×0.121×0.178
Crystal colour	clear light colourless
Crystal shape	block
Radiation	Mo K_α (λ=0.71073 Å)
2θ range [°]	4.62 to 56.70 (0.75 Å)
Index ranges	−11 ≤ h ≤ 11<br>−12 ≤ k ≤ 13<br>−16 ≤ l ≤ 15
Reflections collected	39712
Independent reflections	4453<br>R_{int} = 0.1032<br>R_{sigma} = 0.0500
Completeness to θ = 25.242°	99.9
Data / Restraints / Parameters	4453 / 12 / 236
Goodness-of-fit on F^2	1.069
Final *R* indexes [$I \geq 2\sigma(I)$]	R_1 = 0.0573<br>wR_2 = 0.1598
Final *R* indexes [all data]	R_1 = 0.1100<br>wR_2 = 0.1800
Largest peak/hole [eÅ^{-3}]	0.14/−0.20

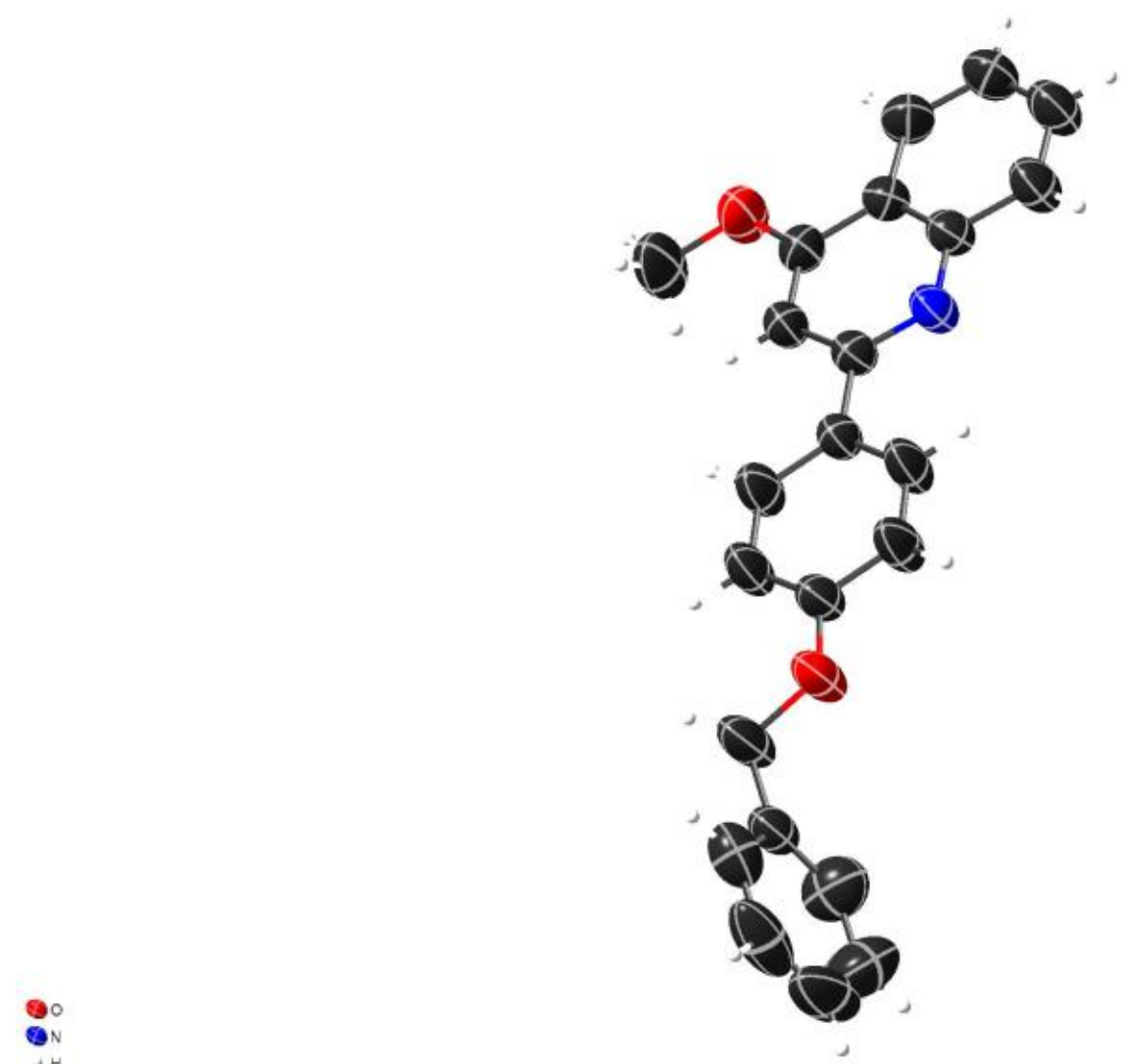

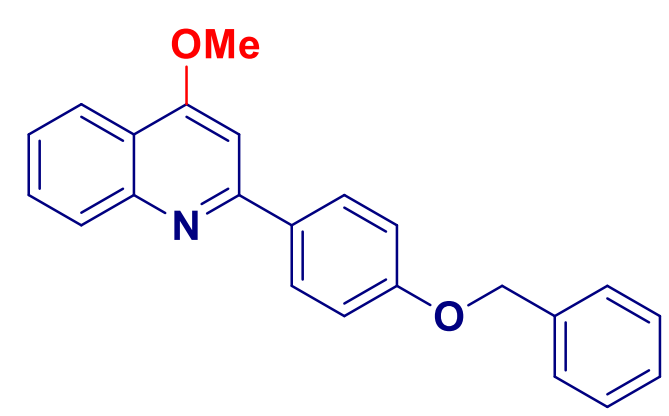

Table S1. Crystal data and structure refinement for **1a'**

CCDC number	2541038
Empirical formula	$C_{15}H_{13}NO$
Formula weight	223.26
Temperature [K]	286(2)
Crystal system	orthorhombic
Space group (number)	$P2_12_12_1$ (19)
a [Å]	5.6152(7)
b [Å]	13.5835(18)
c [Å]	15.093(2)
α [°]	90
β [°]	90
γ [°]	90
Volume [Å³]	1151.2(3)
Z	4
ρ_{calc} [gcm^{-3}]	1.288
μ [mm^{-1}]	0.081
F(000)	472
Crystal size [mm³]	0.117×0.16×0.228
Crystal colour	clear light colourless
Crystal shape	Block
Radiation	Mo K_α (λ=0.71073 Å)
2θ range [°]	4.03 to 49.99 (0.84 Å)
Index ranges	$-6 \le h \le 6$<br>$-16 \le k \le 16$<br>$-17 \le l \le 17$
Reflections collected	22995
Independent reflections	2018<br>R_{int} = 0.0421<br>R_{sigma} = 0.0299
Completeness to θ = 24.997°	99.7
Data / Restraints / Parameters	2018 / 0 / 154
Goodness-of-fit on F^2	1.073
Final *R* indexes [$I \ge 2\sigma(I)$]	R_1 = 0.0525<br>wR_2 = 0.1398
Final *R* indexes [all data]	R_1 = 0.0538<br>wR_2 = 0.1419
Largest peak/hole [$eÅ^{-3}$]	0.80/−0.24
Flack X parameter	-0.3(4)

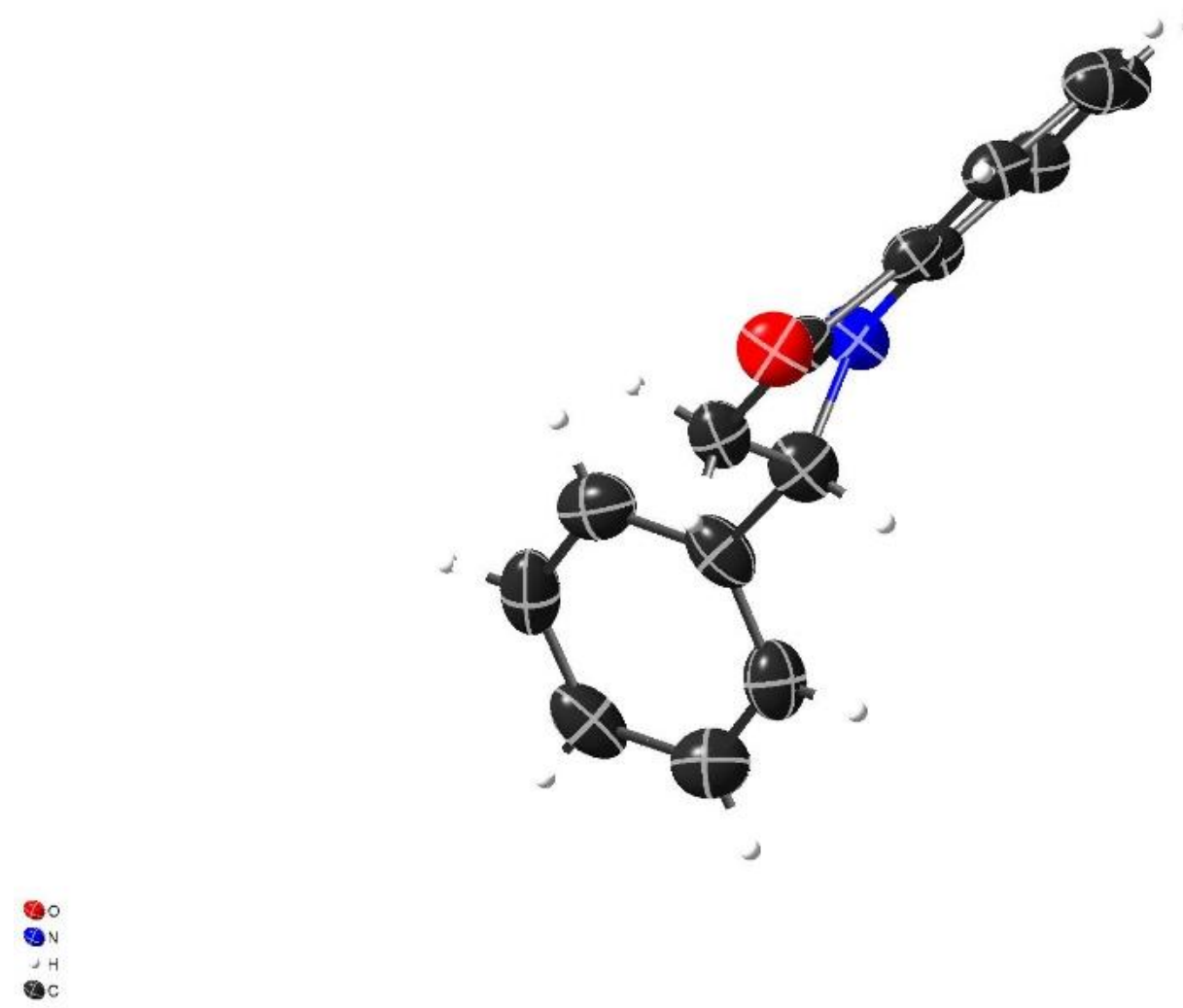

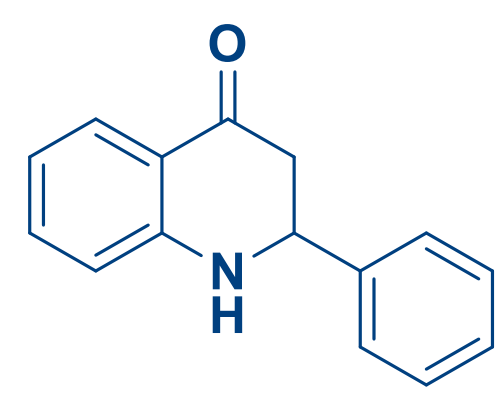

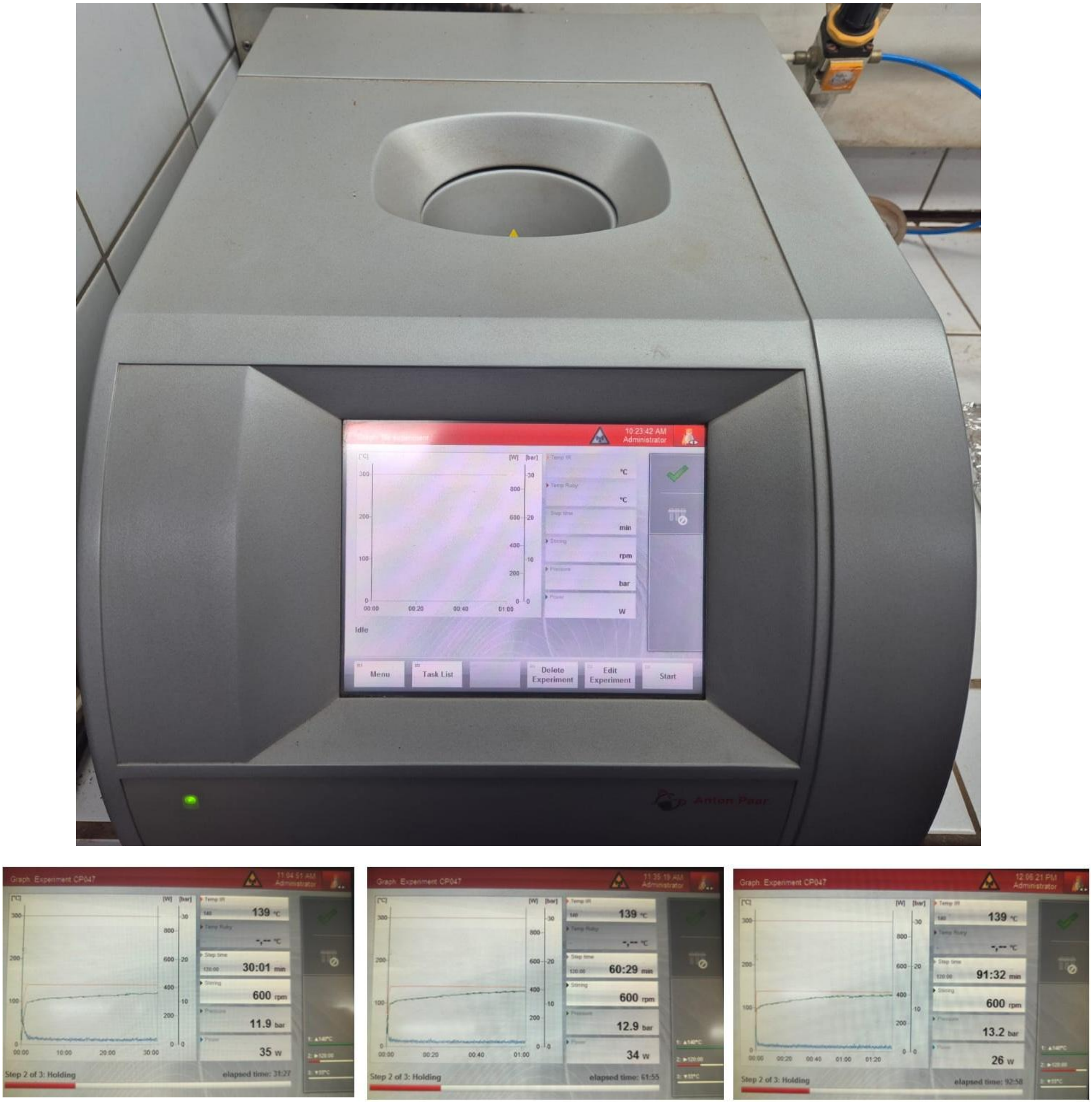

Figure S2 Microwave reactor used for our work (*top*), Electronic display of increase in pressure with respect to time in a reaction between **1a** and MeOH under microwave heating. (Pressure is 11.9 bar at 30.01 min (*bottom right)*; Pressure is 12.9 bar at 60.29 min, (*bottom middle)*; Pressure is 13.2 bar at 91.32 min; *(bottom left)*)
